# Aromaticity of substituted 8-hydroxyquinolines in their free or bidentate states in tricarbonyl rhenium(i) complexes †

**DOI:** 10.1039/d5ra02589c

**Published:** 2025-07-10

**Authors:** Sławomir Ostrowski, Małgorzata Jarończyk, Jan Cz. Dobrowolski

**Affiliations:** a Institute of Nuclear Chemistry and Technology 16 Dorodna-Street 03-195 Warsaw Poland j.dobrowolski@nil.gov.pl; b National Medicines Institute 30/34 Chełmska Str. 00-725 Warsaw Poland

## Abstract

Some 8-hydroxyquinolines (8-HQs) and some of their metal complexes are bioactive. Thus, knowing whether the ligand properties are transferred to the complex is vital. Herein, the aromaticity of the pyridine and phenolic rings of substituted 8-HQs, free with and without intramolecular H-bond and chelated in Re(i) complexes, was studied using the geometrical HOMA and magnetic INICS indices. The 8-HQs were substituted in every position with BH_2_, CN, C≡CH, Cl, and NH_2_ groups of diversified σ- and π-electron donor–acceptor properties. In the H-bonded 8-HQs, the BH_2_ and NH_2_ groups stabilize different positions. The stabilization pattern is similar for the non-H-bonded 8-HQs but quite different in the Re(i) complex. The HOMA and INICS values of the pyridine and phenolic rings show that they are geometrically and magnetically aromatic irrespective of the substituent type, substituent location, H-bond presence, or complex formation. Forming or breaking the O–H⋯N intramolecular H-bond does not essentially affect the two types of aromaticity. In contrast, chelating the Re(i) system with 8-HQ changes both so that the aromaticity of the H-bonded and chelated systems does not correlate. Hence, the aromaticity of the 8-HQ rings is not transferred from the free ligand to the complex with Re(i). Additionally, for H-bonded 8-HQs, the geometrical and magnetic aromaticity do not correlate, but a weak correlation trend can be observed for the complex.

## Abstract 2

The HOMA and INICS indices show that the pyridine and phenolic rings of 8-HQs are aromatic irrespective of the substituent type and H-bond or complex formation. They do not correlate for H-bond systems, but a weak trend exists for the complexes.

## Graphical abstract

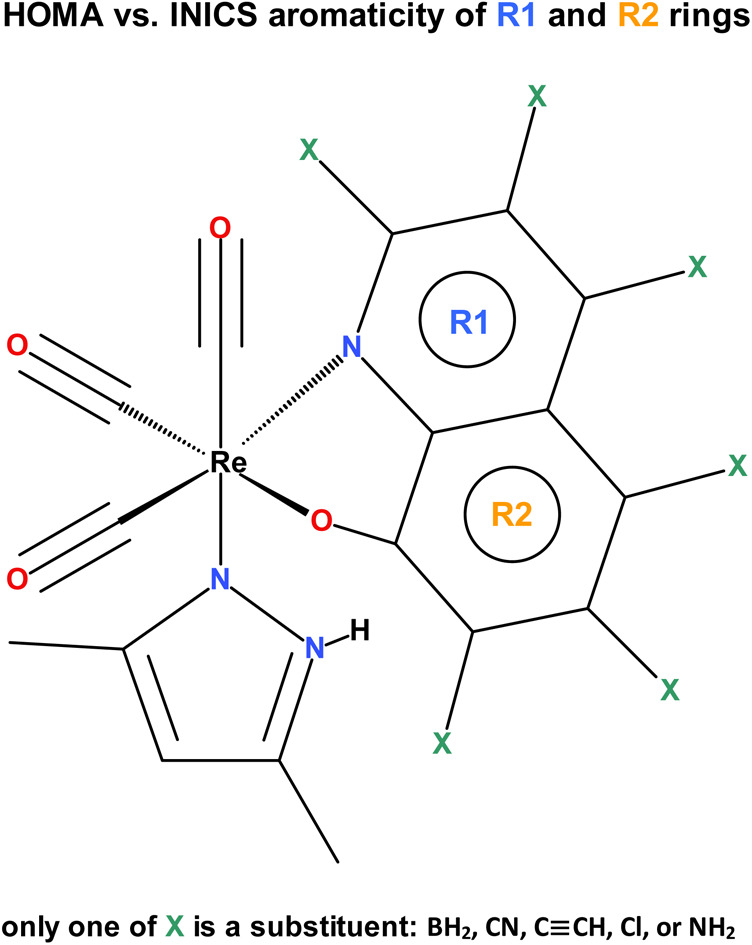

## Introduction

Several 8-hydroxyquinolines (8-HQs) are used as medicines. For example, the antibacterial and antifungal clioquinol (5-chloro-8-hydroxy-7-iodoquinoline) is used for skin infection treatment and exhibits anti-Alzheimer activity; the antibacterial and anticancer nitroxoline (8-hydroxy-5-nitroquinoline) and antiamebial iodoquinol (5,7-diiodo-8-hydroxyquinoline) are also used.^1–3^ However, tricarbonyl rhenium(i) complexes bidentate with 8-hydroxyquinolines also exhibit antibiotic and anticancer activities.^4^^and ref. therein^ Among the 12 new [Re(CO)_3_(L_N,O_)L_N_] complexes (L_N,O_ = 8-hydroxyquinoline and its 2-methyl- and 5-chloro-derivatives; L_N_ = imidazole, 2-methylimidazole, dimethylpyrazole, and 3-phenylpyrazole)^4^ synthesized and characterized by single-crystal X-ray diffraction, and molecular spectroscopy, most showed antibacterial action lower than that of the corresponding free ligand. The 8-hydroxyquinolines exhibited antibacterial potency against *E. coli*, *S. aureus*, and *E. faecalis*, showing the highest inhibitory effect for 5-chloro-8-hydroxyquinoline but not against *P. aeruginosa*. However, the complex with 2-methyl-quinoline and imidazole was 4-fold more active against *P. aeruginosa*. The cytotoxicity of the complexes was evaluated against human acute promyelocytic leukemia (HL-60) and ovarian (SKOV-3), prostate (PC-3), and breast (MCF-7) cancer cell lines, and non-cancerous breast cells (MCF-10A). Although only 8-hydroxyquinoline and 5-chloro-8-hydroxyquinoline free ligands exhibited activity against solid tumor cells, only complexes with 2-methyl-8-hydroxyquinolinato ligand showed activity towards all cell lines higher than the pure ligands. Significant results were obtained for the HL-60 leukemia cells, which exhibited high sensitivity to all complexes (IC_50_ = 1.5–14 μM) and even better to free HQ (IC_50_ = 2 μM) and ClHQ (IC_50_ < 2 μM) ligands.

However, compound bioactivity is determined by numerous factors^5–9^ modeled with abundant molecular descriptors used in QSAR analyses.^10,11^ A sharp understanding of the descriptors is desirable for comprehending QSAR correlations. Meanwhile, the activity of a free ligand is often supposed to justify the activity of metal complexes with this ligand. Aromaticity is one of the central molecular properties, which, although ambiguously defined, cannot be undervalued when considering molecular activity and bioactivity.^12–19^ However, the variation of the ligand aromaticity upon the formation of the metal complex has been insignificantly considered.^20–29^

In 2007, the aromaticity of cyclopentadienyl ligands in organoaluminum complexes was studied with X-ray diffraction supported by DFT calculations using the HOMA geometrical aromaticity index.^20^ The HOMA values of the ligand diversely bound to the aluminum center vary from 0.8 to −0.5, indicating that its character changes from being aromatic, as in the uncomplexed cyclopentadienyl anion, to an anti-aromatic cyclopentadiene structure. The aromaticity of molecules with different cyclopentadiene moiety fused with naphthalene systems was considered model ligands of Li in computational studies and ligands of Fe in X-ray investigations.^21^ It appeared that the cyclopentadienyl moiety is always more aromatic in terms of the NICS(0)_*zz*_ index in the uncomplexed than in the Li-complexed system. This is due to the formation of C–C bonds with an increased local double-bond character in the complex. However, the aromaticity of the terminal benzene rings usually slightly increases in metal complexes.

The aromaticity of the 8-hydroxyquinoline anion, neutral molecule, zwitterion, cation, and its Mg and Al complexes was studied in 2012.^22^ Using geometrical HOMA, magnetic NICS(0) and NICS(1), electronic PDI and FLU, and energetic ASE aromaticity indices, a relatively consistent image of aromaticity changes was obtained although some deviations appeared. The aromaticity of the pyridine moiety was high but varied erratically with the index applied. The aromaticity of the phenolic increased monotonically in the following order: anion < zwitterion < metal complex < neutral < cation. Finally, the total aromaticity increased in the following order: anion < zwitterion < neutral < cation < metal complex. The differences in the indices' indications were explained by the multidimensional character of the aromaticity phenomenon, where in one dimension of an “aromaticity space”, a molecule can be more aromatic than another.

The effect of the central ion on the aromaticity of the chelated acetylacetonate was studied in 2016.^23^ The aromaticity of such a ring is less evident than that of the cyclopentadienyl's π-electron structure directly interacting with the central metal ion. The acetylacetonate ligand is formed with a central metal atom, a ring with six π-electrons, and can be considered aromatic. In the studied systems, metal complexes interacted with the *para*-substituted benzene ring. An atypical aromaticity descriptor was used: the benzene's C–H bond angle with the acetylacetonate plane. The C–H bond was directed to the center of the plane of the acetylacetate, chelating the Be, Cu, Ni, Fe, Ru, Cd, and Pt ions. Interestingly, the angle varied linearly with Taft's σ_R_-value^24^ of the benzene's *para*-substituent. In 2017, for a series of Au(iii) complexes with N-heterocyclic ligands, it was shown that the aromatic character of the nitrogen-containing ring in gold(iii) complexes (as measured by HOMA, NICS and NCI indices) is reduced compared to the same ring of uncoordinated ligands.^25^

A unique problem of the coexistence of two types of aromaticity in bicyclic M_2_L_2_ systems, where M denotes Cr, Mo, or W, and L denotes an amidinate ligand, was investigated in 2021.^26^ A Hückel aromaticity connected with four ligand π electrons and two metal–metal δ-electrons present in a single ring exist together with a Craig–Möbius aromaticity through the delocalization of π electrons of both ligands and metal orbitals with 10π electrons and a double twist. The metal–metal bond order and the π and δ electron conjugation increase down the group; thus, the Hückel and Möbius aromaticity also increase down the group. Osmapentalyne, osmapentalene, and osmapyridinium complexes were studied with DFT calculations for bonding and adaptive aromaticity.^27–29^ The species display aromaticity (determined with ΔBL, NICS, HOMA, and ACID indices) in the ground S_0_ state. However, in the first triplet T_1_ state, they could be aromatic, nonaromatic, or antiaromatic depending on the excitation mode and probably exceptional antiaromatic pentalene-type ligands. Such behavior was proposed to be termed adaptive aromaticity.^29^ Porphyrinoid Pd(ii) complexes exhibiting Hückel aromaticity have recently been studied.^30^ Owing to their 26 π electrons, the complexes exhibit tunable aromaticity and NIR absorption of up to 1060 nm.

Recently, the effect of the spin state of the metal center in spin crossover compounds on the aromaticity of ligands has been studied for model iron(ii) complexes.^31^ It was found that the change in the spin state of the central iron atom did not affect the aromaticity of bipyridine ligands, but the aromaticity of formazanate ligands decreased in the high-spin state. This change in aromaticity owing to the change in the spin state can be considered a clue for non-innocent ligands.

In this study, we examine the aromaticity of the rings of substituted 8-hydroxyquinoline free and bound in the tricarbonyl rhenium(i) complexes using the HOMA^32–39^ and INICS^40–49^ aromaticity indices. The HOMA geometrical aromaticity index is based on bond distances and has been defined using experimental values.^32–35^ The index has a simple and precise meaning because its mathematical formula satisfies the axioms of a similarity function between the examined and the archetypal aromatic benzene ring.^39^ If a molecule is geometrically similar to benzene, it is also chemically similar to it and thus is aromatic. The NICS index was first defined as the opposite value of the absolute magnetic shielding calculated for the probe point at the ring center, NICS(0).^40^ Shortly after, NICS(1), calculated at 1 Å over the ring center, appeared to be more robust.^41^ After several subsequent modifications, the NICS scan became preferred to provide a new indication of diamagnetic and paramagnetic ring currents.^43^ In 2019, Stanger proposed the integral NICS aromaticity index,^44^ which, as shown by Berger and Dimitrova *et al.* in 2022,^45,46^ is physically justified by its relation to the ring current *via* Ampère–Maxwell's law—one of the laws on which classical electromagnetism is grounded. INICS is more justified than NICS(0) because the middle of the ring is strongly influenced by the σ-electron system of the rings. In contrast, the INICS index is dominated by the NICS-scan branches determined by the long-range π-electron system of the ring. Integrating the NICS scan releases the popular NICS(1) index from the arbitrariness of applying values taken at 1 angstrom above or below the ring face, rationalized mainly by early computational experiments.^41^ Moreover, for non-planar rings, as in fullerenes or chiral aromatics, INICS yields one value. NICS(1) is split into two, characterizing two ring faces rather than the ring itself.^47,48^ The relation between INICS and any NICS taken at a point can thus be compared to the integral and in-point intensity of an absorption spectroscopy band, of which only the former has a physical sense. In 2022, we demonstrated that the integral NICS, INICS, is the most robust and indicative in evaluating the aromaticity of the aromatic amino acids.^48^ We recently studied the ring's aromaticity in the pyridine[*m*,*n*]diazepines based on the integral INICS index.^49^ The six-membered pyrido rings appear to have negative INICS_*ZZ*_ values and can be aromatic only if not protonated at the N-atom. In contrast, all protonated pyrido rings exhibit meaningful positive INICS_*ZZ*_ values and can be considered as antiaromatic.

This study differs from the so-far researched aromaticity of metal complexes in the following aspects: (i) we contrasted the analysis using the geometrical and magnetic aspects of aromaticity; (ii) we combined investigation of the aromaticity with the systematic scan of the substituent effect in its both aspects influence on π- and σ-electron systems of 8-hydroxyquinoline; (iii) we placed the investigation to the context of the change in the property of a free ligand on the change in this property of the metal complexes where both the ligand and the complex can gain or can lose its biological activity after forming or leaving the complex.

## Results and discussion

**Scheme 1:**
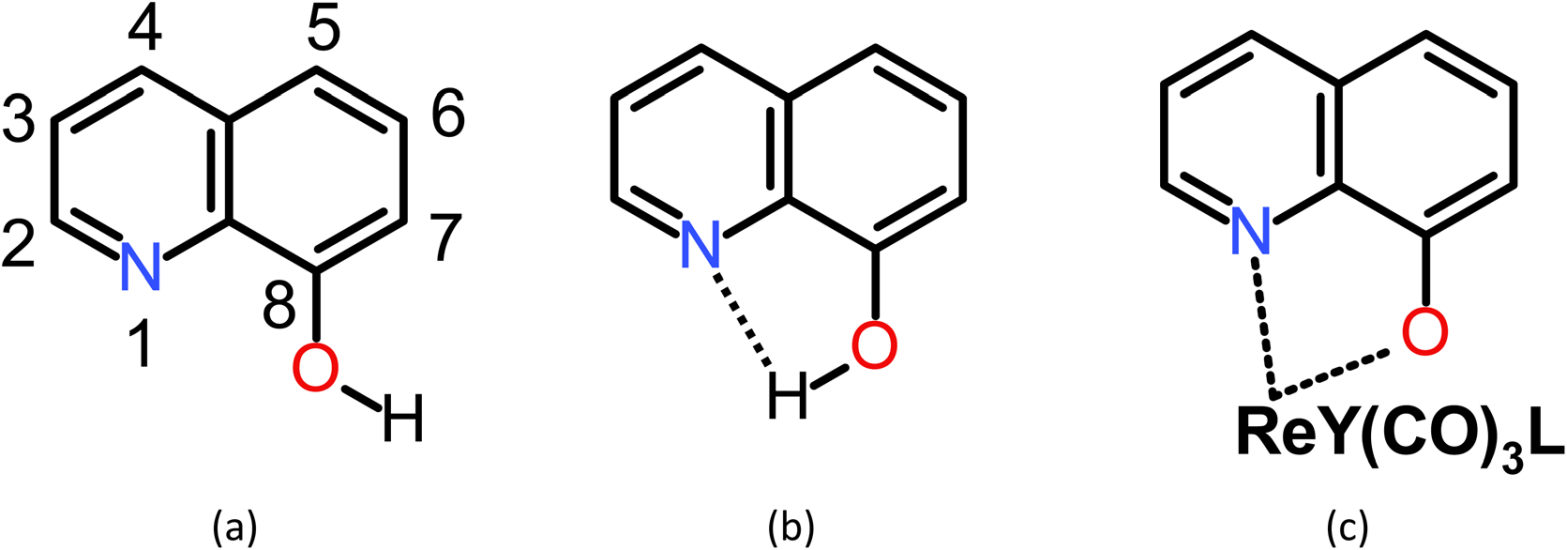
8-Hydroxyquinoline with (a) a broken intramolecular H-bond, (b) formed intramolecular H-bond, and (c) in complex with a Re(i) ion. Y and L denote a counterion and a (monodentate) ligand, respectively.

**Scheme 2:**
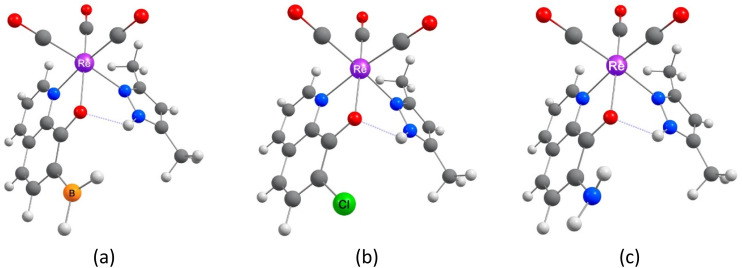
Three example structures of the optimized ReY(CO)_3_L complexes (Y = BH_2_-, Cl-, and NH_2_-substituted 8-HQ at position 7, (a), (b), and (c), respectively, and Y = dimethylpyrazole). Images of all optimized structures can be found in Scheme S1.†

Quinoline comprises fused pyridine and benzene rings in such a way that the pyridine's N-atom is adjacent to the bond in common for the two rings.^50^ In the 8-hydroxyquinoline (8-HQ), the OH group is attached to the benzene ring adjacent to the bond in common, so forming an intramolecular OH⋯N hydrogen bond with the pyridine's N-atom is possible. However, the H-bond may break, the hydroxyl H-atom may dissociate, and a metal ion may replace the H-bonded H-atom and coordinate with the N and O atoms (Scheme 1). In Scheme 2, we show three example structures of the optimized ReY(CO)_3_L complexes, where Y = BH_2_-, Cl-, and NH_2_-substituted 8-HQ at position 7 and Y = dimethyl pyrazole. All optimized structures can be found in Scheme S1.†

### Energetics and the substituent effect

The properties of 8-hydroxyquinoline can be modified by substitutions at position 2–7, where position 2–4 defines the C atoms in the pyridine ring and position 5–7 in the phenolic ring (Scheme 1). We consider substitution in every one of these positions with five substituents of diversified σ- and π-electron donor–acceptor properties: BH_2_, CN, C≡CH, Cl, and NH_2_. The sEDA substituent effect descriptors,^51,52^ reflecting the group electronegativity, are as follows: 0.173, −0.157, −0.161, −0.264, and −0.451, which means that BH_2_ donates 0.173 of the electrons charges to the σ-electron structure of 8-HQ, while the other substituents withdraw the respective amounts of charge from this very structure. The pEDA substituent descriptors,^51,52^ reflecting the resonance effect, are as follows: −0.142, −0.035, −0.010, 0.062, and 0.145, which means that BH_2_, CN, and C≡CH withdraw electron charge from the 8-HQ's π-electron structure, while the Cl and NH_2_ groups supply the respective amounts of charge to it.

**Fig. 1:**
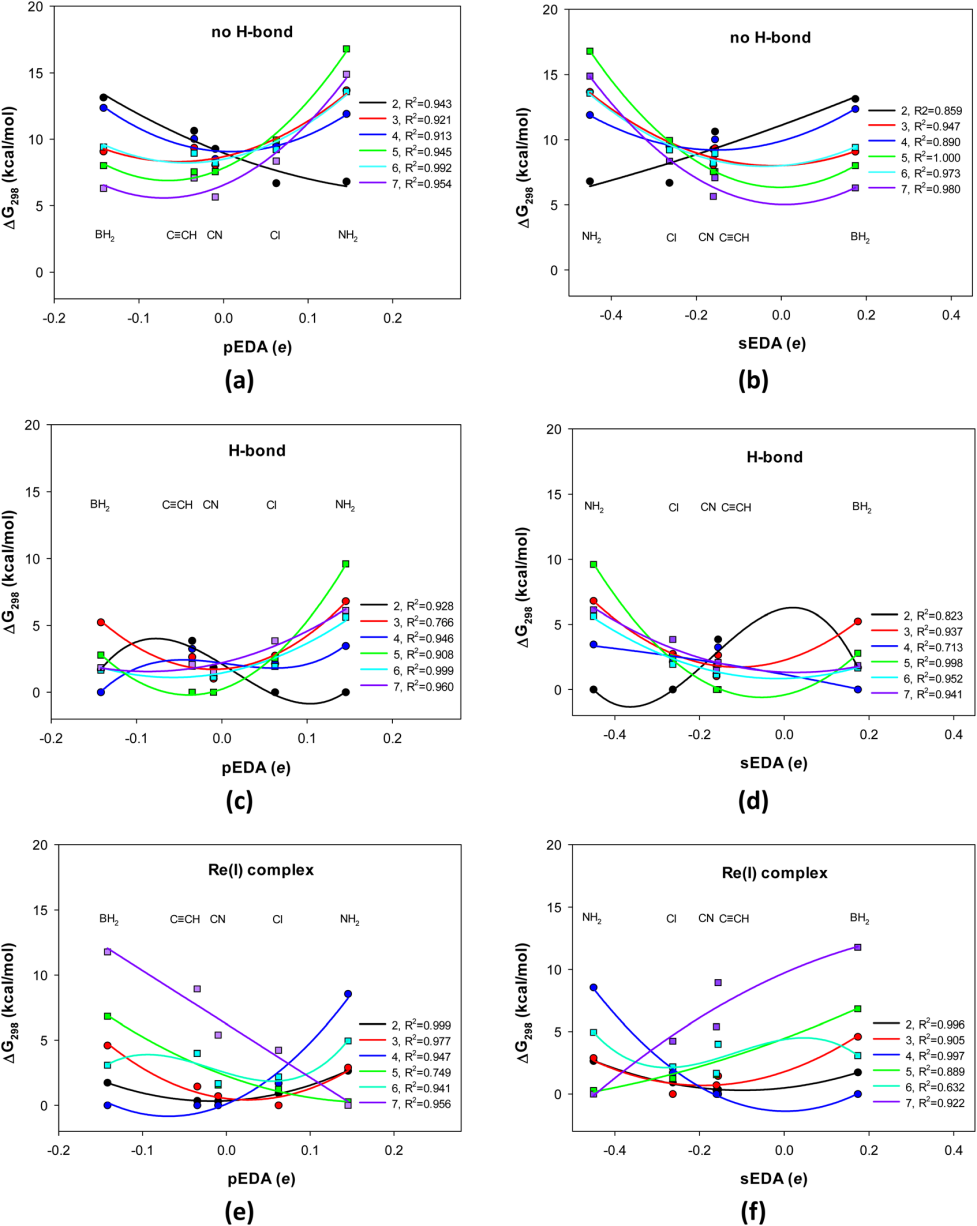
Change in the 8-hydroxyquinoline's Gibbs free energy differences (298 K) with the pEDA and sEDA substituent effect descriptors: (a) and (b) molecule with a broken intramolecular H-bond, (c) and (d) molecule with a formed intramolecular H-bond, and (e) and (f) molecule as a bidentate ligand in the Re(i)(CO)_3_L complex, where Y and L denote a counterion and a (monodentate) ligand, respectively. Circles correspond to substitutions in the pyridine positions 2 (black), 3 (red), and 4 (blue), while squares correspond to substitutions in phenolic positions 5 (green), 6 (light blue), and 7 (violet). The pEDA descriptor increases, while sEDA decreases, in the BH_2_, CN, C≡CH, Cl, and NH_2_ series.

Thus, the BH_2_ group is σ-electron donating but π-electron withdrawing, CN and C≡CH are σ- and π-electron withdrawing, and Cl and NH_2_ are σ-electron withdrawing but π-electron donating. Additionally, the effect on the σ−electron structure is local and limited to the atoms neighboring the substituted one, while the impact on the π-electron structure is pronounced on the entire quinoline π-electron system. Notably, for the BH_2_, CN, C≡CH, Cl, and NH_2_ series, the sEDA values decrease, whereas the pEDA ones increase. In general, although the descriptors of substituent effects on σ- and π-electron systems (group electronegativity and resonance effect descriptors) are linearly independent and do not correlate, here, the substituents are specifically chosen, and the greater the former, the lower the latter. This can be observed in the approximate symmetry of the curves presented in (Fig. 1a, c and e*vs.*1b, d and f, respectively). Finally, note that the N-atom incorporated in the pyridine ring withdraws σ- and π-electrons from the ring (sEDA(II) = −0.368, pEDA(II) = −0.120),^53^ and the OH group withdraws electrons from the σ-skeleton but donates electrons to the π-system of the phenolic ring (sEDA = −0.623, pEDA = 0.121).^51,54^

Here, we relate the energy or Gibbs free energy in 298 K (Fig. 1 and S1, Tables S1–S5†) to the X-substituted 8-HQ with the lowest energy, X = BH_2_, CN, C≡CH, Cl, and NH_2_. For the systems with and without intramolecular OH⋯N hydrogen bond and in a complex with the Re(i)-ion, the most stable form is taken the same to expose significant stabilization of the one with the H-bond formed (a matter of *ca.* 7 ± 3 kcal mol^−1^, Fig. 1a*vs.*1c and 1b*vs.*1d).

The relative stability depends on the substituent electron donor–acceptor properties and its position in the molecule (abscissae at pEDA and sEDA coordinates and the curve color, respectively, Fig. 1). It also depends on whether the system has an intramolecular H-bond and whether it is in a complex with the Re(i)-ion (Fig. 1). Notice that the curves in Fig. 1 play only an indicative and an approximative role because, with five substituents, one cannot precisely follow the substituent effects. However, increasing the number of substituents (for three systems and six substituent locations) was not possible in this project.

We can examine the substituent effect from two perspectives: (i) the substituents that stabilize or destabilize a given substituent position and (ii) how the stabilization of a given substituent position varies with the substituent properties. In the first, we analyze changes at a single abscissa, while in the second, the variation of the graph of a particular color is examined (Fig. 1).

Consider the H-bonded 8-HQs (Fig. 1c and d). The π-electron withdrawing and σ-electron donating BH_2_ group stabilizes position 4 the most, *para* to the pyridine N-atom and position 3 the least, *meta* to it. In contrast, the π-electron donating and σ-electron withdrawing NH_2_ group stabilizes most position 2, *ortho* to the pyridine N-atom, and the least stabilizes position 5, *para* to the OH group. In significant stabilization of position 2, an N–H⋯N close contact could also contribute. Moreover, the less π-electron donating and σ-electron withdrawing Cl group stabilizes this position, too. The moderately π- and σ-electron withdrawing C≡CH and CN groups stabilize position 5 *para* to the OH group, and the least stabilize position 2, *ortho* to the N-atom. The stabilization sequence is somewhat similar for the non-H-bonded 8-HQs (Fig. 1a and b).

The stabilization pattern varies for the complex with Re(i) (Fig. 1e and f). The BH_2_ group still stabilizes the 4 position *para* to the N-atom the most but stabilizes the 7 position *ortho* to the OH group the least. However, the NH_2_ group stabilizes the most the 7 and 5 positions *ortho* and *para* to the OH group and the least the 4 position *para* to the N-atom. Similar to BH_2_, the C≡CH and CN groups stabilize the 4 and 2 positions *para* and *ortho* to the N-atom the most and the 7 position *ortho* to the OH group the least. The Cl group stabilizes the most 3, *meta* position to the N-atom the most and the least the 7 position *ortho* to the OH group.

The substituent effect works monotonically at only a few substituent positions, while for the others, it is quadratic or more complex (Fig. 1). In the case of 8-HQs without hydrogen bonds, this is position 2 *ortho* to the N atom. In this case, stabilization increases from the BH_2_ to the NH_2_ substituent as the pEDA parameter increases and sEDA decreases. In the case of H-bonded systems, it is position 4, *para* to the N atom. Stability in this position increases from the NH_2_ to the BH_2_ group. In contrast, monotonic variation in the system's stability in the complex occurs for substitutions of the phenolic ring in the *ortho* and *para* positions relative to the hydroxyl group and practically for position 2, *ortho* to the N-atom. For the former two, the stability increases from the BH_2_ to the NH_2_ group, while for the latter in the opposite direction.

### Geometrical and magnetic aromaticity and the substituent effect

**Fig. 2:**
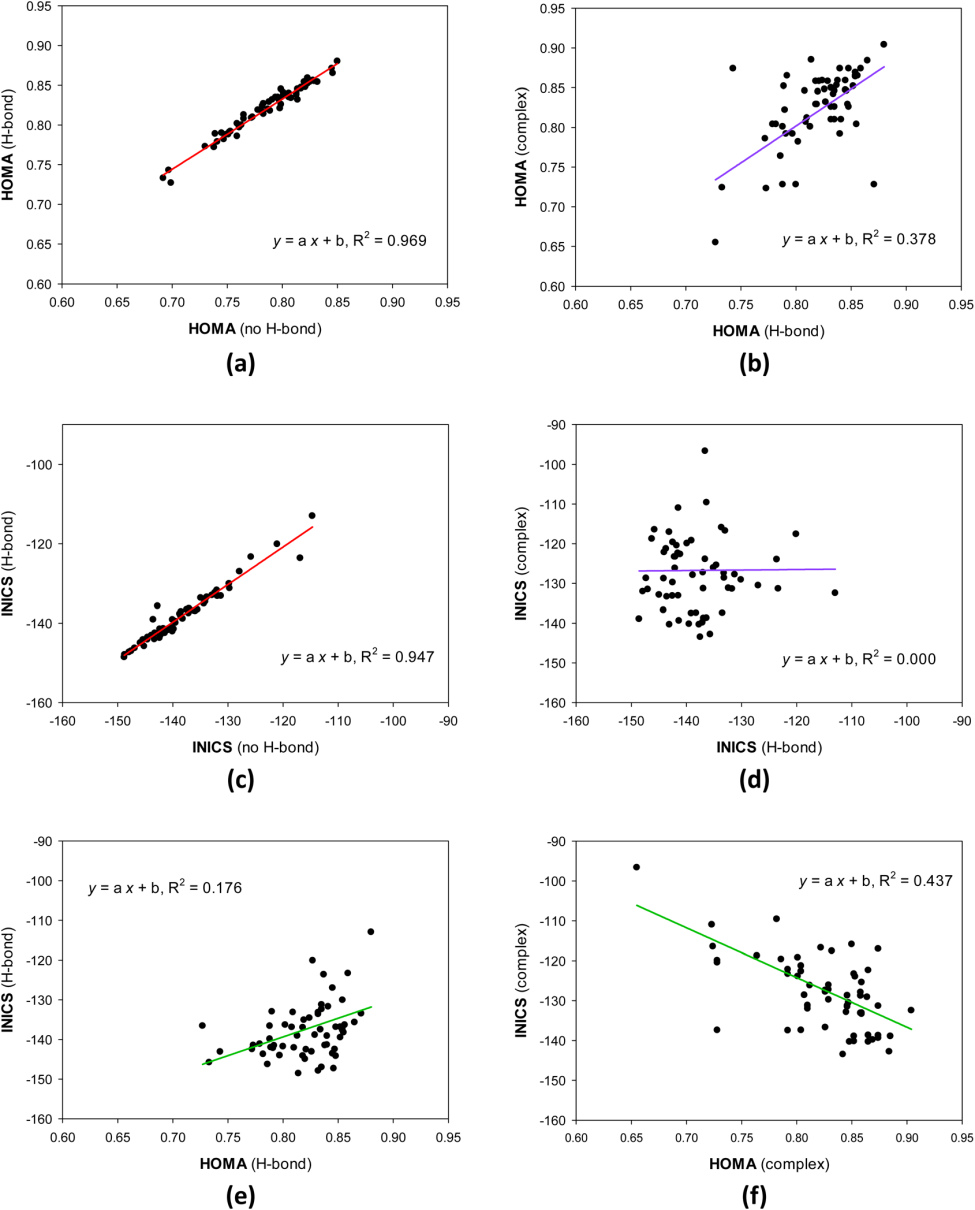
Similarities and differences between geometrical HOMA (a and b) and magnetic INICS (c and d) aromaticity of diverse 8-hydroxyquinoline forms, and aromaticity estimated using the two contrasted HOMA and INICS aromaticity indices for H-bonded (e) and complexed (f) 8-hydroxyquinolines.

**Fig. 3:**
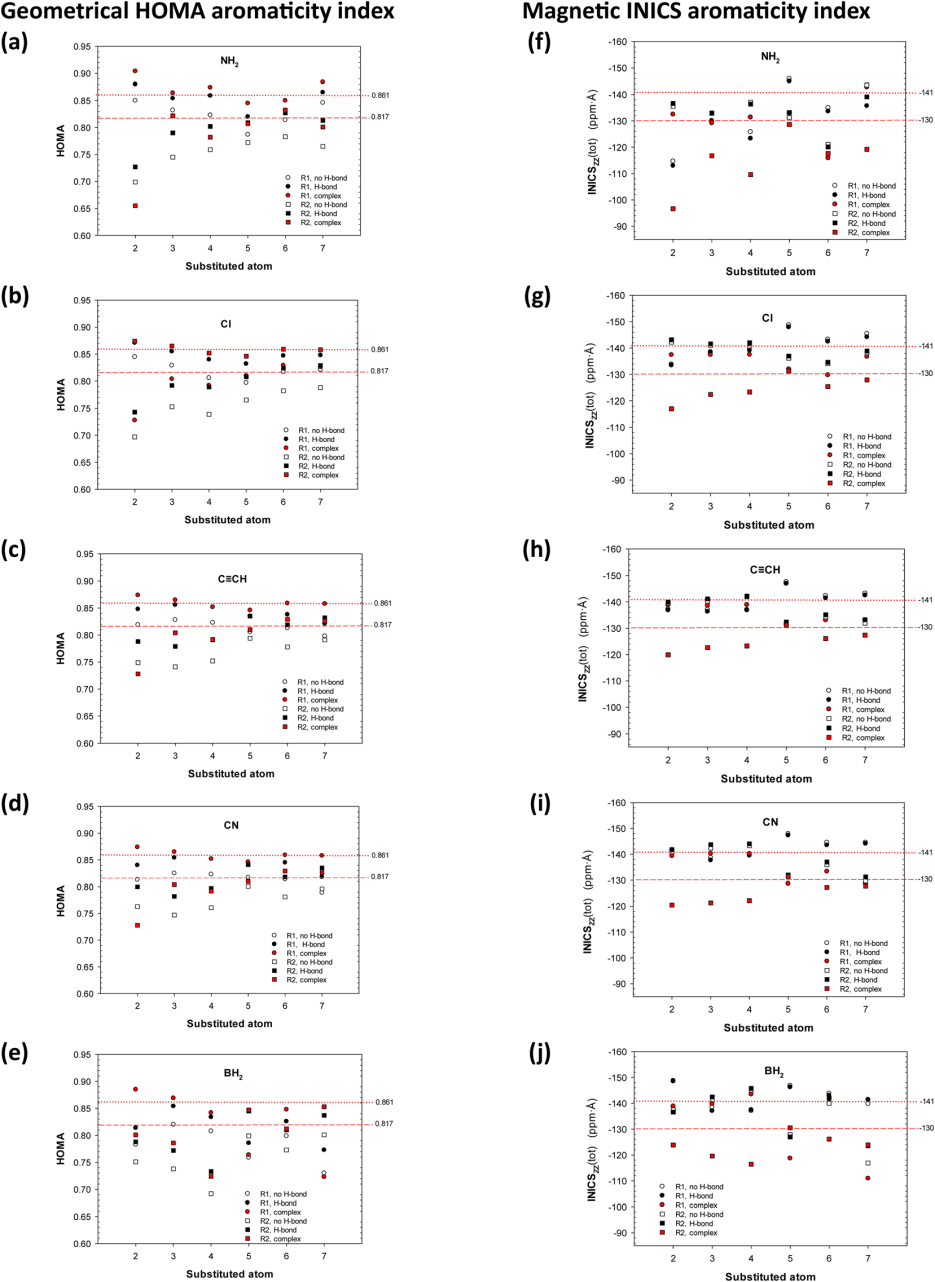
Variations in the HOMA geometrical (a–e) and integral magnetic INICS (f–j) aromaticity indices of the pyridine (R1, circles) and phenolic (R2, squares) rings in 8-hydroxyquinolines substituted with the (a and f) NH_2_, (b and g) Cl, (c and h) C≡CH, (d and i) CN, and (e and j) BH_2_ groups. Points corresponding to systems with a broken intramolecular H-bond, a formed intramolecular H-bond, and in the bidentate Re(i)(CO)_3_L complex are empty, black, and red, respectively. The dotted and dashed red straight lines correspond to the aromaticity of the pyridine and phenol rings, respectively, in complex with unsubstituted 8-hydroxyquinoline.

The geometrical HOMA and magnetic INICS aromaticity indices determined for all 8-hydroxy-quinolines are presented in Tables S6 and S7† and Fig. 2 and 3. Fig. 2 depicts the similarities and differences between the aromaticity of the three 8-HQ forms estimated using two contrasted aromaticity indices. It is striking that forming or breaking the O–H⋯N intramolecular H-bond does not essentially affect the geometrical (Fig. 2a) or magnetic INICS (Fig. 2c) aromaticity. In contrast, chelating the Re(i) system with 8-HQ changes the two, so there is no correlation between the aromaticity of the H-bonded and chelated systems (Fig. 2b and d). Interestingly, for the H-bonded 8-HQ, the geometrical and magnetic aromaticity also do not correlate (Fig. 2e), but for the complex, a weak correlation trend can be observed (Fig. 2f).

It is essential to understand why there is no correlation between the HOMA and INICS indices of 8-hydroquinolines with internal hydrogen bonds and ligands in complexes with Re(i). Or does one or both indices incorrectly show the order of aromaticity of 8-hydroquinolines? This is related to the multidimensionality of the aromaticity phenomenon.^55^ HOMA measures geometric similarity to the benzene ring based on bond lengths influenced by the ring's σ- and π-electron structure. In contrast, the INICS index depends predominantly on the π-electron structure of the ring. Moreover, the σ- and π-electron structures are influenced by both substituent effect components of the group attached to the 8-hydroxyquinoline system (estimated by sEDA and pEDA descriptors). In contrast, the π-electron structure, which predominantly determines INICSs, is affected only by the component specified by the pEDA descriptor. All these factors mean that there may be no or only a weak correlation between the indices (Fig. 2).

Each image in Fig. 3 shows in a condensed form the influence of a specific substituent on aromaticity (ordinate) at a given substituent location (abscissa) for the pyridine (R1, circles) and phenolic (R2, squares) rings in 8-HQs without or with intramolecular H-bond or in the Re(i)(CO)_3_L complex: empty, black, and red points, respectively. Thus, the black square in Fig. 3a at an abscissa equal to 2 displays the aromaticity of the phenolic ring R2 when the H-bonded system is substituted by the NH_2_ group in position 2. Analogously, the red circle in Fig. 3a at an abscissa equal to 5 shows the aromaticity of the pyridine ring R1 when the system in the complex is substituted by the NH_2_ group in position 5. The points of the same type are not connected by a function line because the fractional values of the abscissa have no chemical sense.

First, note that the HOMA values of the pyridine and phenolic rings in the unsubstituted quinoline are 0.999 and 1.0, respectively. In the unsubstituted 8-hydroxyquinoline without the intramolecular H-bond, they are 0.771 and 0.820, respectively, while with the H-bond formed, they increase slightly and are 0.813 and 0.848, respectively. In principle, all HOMA values in Fig. 3a–f range from 0.7 to 0.9, and a few cases when HOMA is close to or below 0.7 involve the most strongly acting NH_2_ and BH_2_ groups (Fig. 3a and e). Hence, the two rings remain (geometrically) aromatic irrespective of the substituent kind, substituent location, H-bond presence, or complex formation. Second, observe that for the NH_2_, Cl, C≡CH, and CN groups, the most significant gap between the geometrical HOMA aromaticity of the two rings occurs for the substitution at position 2, *ortho* to pyridine's N-atom. In contrast, the BH_2_ group produces a significant gap when located at position 4, *para* to the N-atom, and 7, *ortho* to the OH group. Interestingly, in the latter case, the aromaticity of the phenolic R2 becomes greater than that of the R1 ring, *i.e.*, opposite that for the NH_2_ group (Fig. 3e*vs.*3a).

Third, for the NH_2_ substitution in the three systems, HOMA indicates that the aromaticity of pyridine is greater than that of the phenolic ring for all substituent positions except 5 and 6. Such regularity also occurs for the C≡CH and CN groups attached to the pyridine ring (positions 2–4) and the BH_2_ group in positions 3 and 4. For the Cl group, the pattern is different (Fig. 2c): for each substituent location, the phenolic ring in the system without the H-bond always has the lowest aromaticity, whereas the phenolic ring in the system in the complex always has the highest aromaticity. The difference ranges from Δ(HOMA) ≈ 0.17 to Δ(HOMA) ≈ 0.10 for positions 2 and 7, respectively. For the C≡CH and CN groups, the least aromatic system is the same (except at position 2), but the highest aromaticity is exhibited by the pyridine ring in the system in the complex. The BH_2_ group affects aromaticity specifically for each substituent position (Fig. 3e).

The order established by magnetic aromaticity according to the INICS index differs from that displayed by HOMA (Fig. 3f–j*vs.*a–e). The INICS values of the pyridine and phenolic rings in the unsubstituted quinoline are −140 and −147 (ppm Å), respectively; in the unsubstituted 8-hydroxyquinoline without the intramolecular H-bond, they are −144 and 147, respectively, while with the H-bond formed, they increase slightly and are −145 and −146, respectively. The ordinate INICS scale is inverted to facilitate comparison with the HOMA index variations, and the most aromatic systems with the lowest INICS are at the top of the images. First, almost always, the phenolic ring R2 of the 8-HQs in the Re(i) complex has the lowest magnetic aromaticity. The BH_2_-substituted systems in positions 5 and 7, *para* and *ortho* to the OH group, are exceptions in which the pyridine ring R1 of the system in the Re(i) complex has the least aromaticity. In complexes 5-substituted by the NH_2_, Cl, and C≡CH groups and complex 6-substituted by the NH_2_ group, the INICS values of the R1 and R2 rings are essentially the same. Second, in the system substituted in the pyridine ring (positions 2–4), the gap between the aromaticity of the R1 and R2 rings is usually much wider for the Re(i) complexes (red points, Fig. 3) than for the free molecules.

## Conclusions

Some 8-hydroxyquinolines (8-HQs) are used as medicines, and some of their metal complexes also exhibit antibiotic and anticancer activity.^4^ In the design of a metallodrug, whether ligand properties are transferred to the complex or lost after ligand chelating appears repeatedly. Herein, we examined the aromaticity of the pyridine and phenolic rings of substituted 8-HQs free with and without intramolecular H-bond and chelated in the tricarbonyl rhenium(i) complexes. The geometrical and magnetic aspects of aromaticity were estimated using the HOMA and integral NICS (INICS) indices, respectively. We considered substitution in every position in the three 8-HQ forms with five substituents of diversified σ- and π-electron donor–acceptor properties: BH_2_, CN, C≡CH, Cl, and NH_2_.

In the H-bonded 8-HQs, we found that the most π-electron withdrawing and σ-electron donating BH_2_ group stabilizes the most position *para* to the pyridine N-atom and the least position *meta*. In contrast, the most π-electron donating and σ-electron withdrawing NH_2_ group stabilizes most positions *ortho* to the pyridine N-atom, and the least stabilizes position *para* to the OH group. The stabilization sequence is similar for the non-H-bonded 8-HQs, but in the complex with Re(i), the BH_2_ group still stabilizes the position *para* to the N-atom the most but the least the position *ortho* to the OH group. However, the NH_2_ group stabilizes the most *ortho* and *para* positions to the OH group and the least *ortho* and *para* positions to the N-atom.

The analysis of the geometrical and magnetic aromaticity of all 8-hydroxyquinolines shows that forming or breaking the O–H⋯N intramolecular H-bond does not essentially affect the two kinds of aromaticity. In contrast, chelating the Re(i) system with 8-HQ changes the two, so there is no correlation between the aromaticity of the H-bonded and chelated systems. Hence, the aromaticity of the 8-hydroxyquinoline pyridine and phenolic rings is not transferred to the complex after ligand chelating. Additionally, the geometrical and magnetic aromaticity do not correlate for H-bonded 8-HQs, but a weak correlation trend can be observed for the complex.

The HOMA and INICS values of the pyridine and phenolic rings show that they are geometrically and magnetically aromatic irrespective of the substituent kind, substituent location, H-bond presence, or complex formation. According to the HOMA index, amongst the NH_2_, Cl, C≡CH, and CN groups, the most significant gap between the aromaticity of the two rings occurs for the NH_2_ group located *ortho* to pyridine's N-atom. In contrast, the BH_2_ group produces a significant gap when located *para* to the N-atom and when attached at position *ortho* to the OH group. HOMA indicates that for the NH_2_ substitution, the aromaticity of the pyridine is greater than that of the phenolic ring for most substituent positions. This also holds for the C≡CH and CN groups attached to the pyridine ring and the BH_2_ group in positions *meta* and *para*. The BH_2_ group affects aromaticity specifically in each position.

The order established by the INICS index differs from that displayed by the HOMA. Almost always, the phenolic ring in the Re(i) complex has the lowest magnetic aromaticity. To understand this fact, we must recognize the role of the pyridine N-atom lone electron pair and the valence electrons of the phenolic O-atom on the π-electron system of the pyridine and phenol rings. In the complexes, the Re-ion withdraws electrons from these two electron-donor centers. However, the N-atom lone electron pair is a part of the σ-electron system of the pyridine ring.^56^ In contrast, the O-atom valence electrons are involved in the π-electron system of the phenol ring. Although in the non-perfectly symmetrical systems, the σ- and π-electron systems are not entirely independent and can be described as “interacting”, *i.e.*, display hyperconjugation effects. Thus, for the pyridine ring, the Re-ion mostly withdraws electrons from the σ-electron system, while for the phenol ring, it mostly withdraws electrons from the π-electron system. Thus, the π-electron system of pyridine is less depleted by Re-ion than that of phenol. Therefore, the magnetic aromaticity of the phenolic ring is remarkably reduced compared to that of the pyridine moiety. The HOMA index measures the cumulative effect on both rings. Thus, for some substituents, such as Cl, the geometrical aromaticity of the phenolic ring is larger than that of the pyridine moiety. In the system substituted in the pyridine ring, the gap between the aromaticity of the two rings is usually much wider for the Re(i) complexes than for the free 8-HQ.

Finally, we must mention a vital reviewer's question about the role of the spin–orbit effect on the aromaticity of ligand rings. Indeed, there are papers showing that if a heavy atom is incorporated into a π-electron system of carbon atoms, the effect on the aromaticity of the whole system can be colossal.^57,58^ Sometimes, a system considered aromatic without the spin–orbit impact considered turns out to be anti-aromatic when this effect is included. Therefore, we calculated the spin–orbit influence on the aromaticity of the rings of the unsubstituted 8-hydroxyquinoline with the tricarbonyl rhenium complex (Fig. S2, Table S8†). We found that in contrast to systems whose heavy metal atom is incorporated into a π-electron carbon atom system and the effect of the spin–orbit on the aromaticity is very large when the rhenium atom is only coordinated with the ligand, the scalar relativistic correction through the pseudopotential on Re is sufficient, and the spin–orbit impact is negligible (Fig. S2, Table S8†).

## Computational

DFT calculations were performed using the Gaussian 16 program package.^59^ The optimizations were performed with the hybrid B3LYP functional,^60,61^ and Dunning's aug-cc-pVTZ basis set^62,63^ was used for all atoms except Re, for which Ahlrichs's def2-TZVPP with pseudopotential was applied.^64,65^ Cartesian *xyz* coordinates for optimized molecules are shown in Table S9.† All optimized structures reached the potential energy minima, confirmed only by real harmonic frequencies. The calculations of the INICS index were possible owing to the ARONICS program written by our group.^66^ ARONICS generates a Gaussian input file from the earlier-optimized molecule's Gaussian log file. The input contains dummy atoms for the NMR shielding constant calculations and for plotting the NICS_*zz*_ scan. The program automatically finds rings in the input structure. ARONICS determines a least-squares-fitted plane for each ring based on the coordinates of the ring-heavy atoms and the vector normal to the plane at the ring center. The step size of the probe points along the normal straight line can vary. We used 0.1 Å for 〈−5; 5〉 and 0.3 Å for 〈−9.8, −5.0〉∪〈5.0, 9.8〉 steps (limits are distances from the ring center). From the NMR calculation outputs, ARONICS returns the NICS values at all probe points (including 0, −1, +1), at the left and right maxima of the scan branches, at the scan minimum, and the NICS_*zz*_ scan plot with its integral INICS, as well as integrals of the left and right scan branches.^48^ In the summer of 2025, a new program, AroCalc by Oliwier Misztal,^67^ replaced ARONICS. An early version of AroCalc is available upon request. The correlations were performed using the commercial SigmaPlot program for Windows ver. 14.^68^

## Author contributions

Conceptualization, J. Cz. D.; methodology, J. Cz. D.; investigation, S. O., M. J., and J. Cz. D.; resources, S. O., M. J., and J. Cz. D.; formal analysis, J. Cz. D.; data curation, S. O. and J. Cz. D.; writing—original draft preparation, S. O., M. J. and J. Cz. D.; writing—review and editing, J. Cz. D.; visualization, S. O., M. J. and J. Cz. D.; project administration, J. Cz. D. All authors have read and agreed to the published version of the manuscript.

## Conflicts of interest

There are no conflicts to declare.

## Supplementary Material

RA-015-D5RA02589C-s001

## Data Availability

All quantum chemical DFT calculations were performed using the commercially available Gaussian 12 software.^53^ The functional^54,55^ and Dunning's aug-cc-pVTZ basis sets^56,57^ were used for all atoms except Re, for which Ahlrichs's def2-TZVPP with pseudopotential was applied.^58,59^ These are accessible directly in the Gaussian program. Cartesian xyz coordinates for optimized molecules are shown in Table S8.† Calculations of the HOMA indices and their components were performed using the commercial Microsoft Excel program, while correlations were performed using the commercial SigmaPlot program for Windows ver. 14.61 All used data are available in the ESI.†
