# Supplementary material for: Aromaticity of substituted 8-hydroxyquinolines in their free or bidentate states in tricarbonyl rhenium(i) complexes
Source: RSC Adv. 2025 Jul 10;15(29):24019–30. doi: 10.1039/d5ra02589c (PMC12242775; doi:10.1039/d5ra02589c)
Supplement: RA-015-D5RA02589C-s001 [file RA-015-D5RA02589C-s001.pdf]

Supplementary Information (SI) for RSC Advances.
This journal is © The Royal Society of Chemistry 2025

Supplementary Information files to

On aromaticity of substituted 8-hydroxyquinolines free or bidentated in tricarbonyl rhenium(I) complex-es

Sławomir Ostrowski,[a] Małgorzata Jarończyk[b] and Jan Cz. Dobrowolski*[b]

[a] Institute of Nuclear Chemistry and Technology, 16 Dorodna-Street, 03-195 Warsaw, Poland

[b] National Medicines Institute, 30/34 Chełmska Str., 00-725 Warsaw, Poland

Table of contents:

#	Title	page
1	**Scheme S1.** The 8-hydroxyquinolines with (a) broken intramolecular H-bond, (b) formed intramolecular H-bond, and (c) in complex with a Re(I) ion.	S2
2	**Fig. S1.** Change of the 8-hydroxyquinoline's energy differences (298 K) with the pEDA and sEDA substituent effect descriptors: (a) and (b) molecule with the broken intramolecular H-bond, (c) and (d) molecule with formed intramolecular H-bond, (e) and (f) molecule as a bidentate ligand in the Re(I)(CO)$_3$L complex, where Y and L stand for a counterion and a (monodentate) ligand. Circles correspond to substitutions in pyridine's positions 2 (black), 3 (red), and 4 (blue), while squares correspond to substitutions in phenolic's positions 5 (green), 6 (light blue), and 7 (violet). The pEDA descriptor increases while sEDA decreases in the BH$_2$, CN, C≡CH, Cl, and NH$_2$ series.	S4
3	**Table S1.** Total energies E (Hartree) and Gibbs free enthalpies in 298 K G_{298} (Hartree) and ΔE and Δ G_{298} differences (kcal/mol) concerning the most stable isomer and isomer population in a hypothetical isomer equilibrium mixture aaccording to ΔE and Δ G_{298} pop_E and pop_{G298} (%) of the substituted 8HQ without OH...N internal hydrogen bond calculated at the B3LYP/cc-pVTZ level.	S5
4	**Table S2.** Total energies E (Hartree) and Gibbs free enthalpies in 298 K G_{298} (Hartree) and ΔE and Δ G_{298} differences (kcal/mol) concerning the most stable isomer and isomer population in a hypothetical isomer equilibrium mixture according to ΔE and Δ G_{298} pop_E and pop_{G298} (%) of the substituted 8HQ with OH...N internal hydrogen bond calculated at the B3LYP/cc-pVTZ level.	S6
5	**Table S3.** Total energies E (Hartree) and Gibbs free enthalpies in 298 K G_{298} (Hartree) and ΔE and Δ G_{298} differences (kcal/mol) concerning the most stable isomer and isomer population in a hypothetical isomer equilibrium mixture according to ΔE and Δ G_{298} pop_E and pop_{G298} (%) of the substituted 8HQ in the Re(I) complex calculated at the B3LYP/cc-pVTZ level.	S7
6	**Table S4** The sEDA and pEDA substituent effect descriptors relate ΔE_{298} for the 8-: molecule with the broken intramolecular H-bond, molecule with formed intramolecular H-bond, molecule as a bidentate ligand in the Re(I)(CO)$_3$L complex	S8
7	**Table S5** The sEDA and pEDA substituent effect descriptors relate ΔG_{298} for the 8-hydroxyquinoline: molecule with the broken intramolecular H-bond, molecule with formed intramolecular H-bond, molecule as a bidentate ligand in the Re(I)(CO)$_3$L complex	S9
8	**Table S6.** Variations of the HOMA geometrical for pyridine (R1) and phenolic (R2) rings in 8-hydroxyquinolines and complex, substituted with the isomer group (Scheme 1) corresponding to the systems with the broken intramolecular H-bond, formed intramolecular H-bond, and in the bidentate Re(I)(CO)$_3$L complex.	S10
9	**Table S7** Different NICS aromaticity indices of ring in 8-hydroxyquinoline (a) no intramolecular H-bond, (b) formed intrtamolecular H-bond and (c) complex with a Re(I) ion (Scheme 1), S - Substituent type, Ring - pyridine (R1) and phenolic (R2) rings in 8-HQs, isomer - substitution in the positions, I – integral NICS (INICS. ppm·Å), I- – integral NICS for the distances below the plane (ppm·Å), I+ – integral NICS for the distances below the plane (ppm·Å), ΔI=I(-) – I(+)(ppm·Å); NICS(0), NICS(1). NICS(-1), and ΔNICS(-1,1)= NICS(-1) - NICS(1) (ppm), MIN1 and MIN2 are minima of the NICS function for distances below and above the ring plane, respectively, and NICS(MIN1) and NICS(MIN2) are the NICS function values in MIN1 and MIN2.	S11
10	**Fig. S2.** The NICSZZ scans for the 8-hydroxyquinoline ligand complexed with the tricarbonyl Rhe-nium moiety calculated at the B3LYP/TZ2p-j level with and without spin-orbit effect included (see legend). Although, the spin-orbit effect on the aromaticity of the system is colossal (Table S8), in the case when the Rhenium atom is only coordinated with the ligand, the spin-orbit im-pact on the magnetic aromaticity of the 8-hydroxyquinoline ligand rings is negligible.	S16
11	**Table S8.** The ZZ elements of the shielding tensors (ppm) for the probe points calculated for the 8-hydroxyquinoline ligand complexed with tricarbonyl Rhenium moiety calculated at the B3LYP/TZ2p-j level with and without spin-orbit effect included.	S17
12	**Table S8** Cartesian xyz coordinates for optimized molecules for: No H-bond, H-bond and complex with a Re(I) ion using B3LYP/aug-cc-pVTZ empiricaldispersion=GD3.	S20

Scheme S1a. The 8-hydroxyquinolines with broken intramolecular H-bond

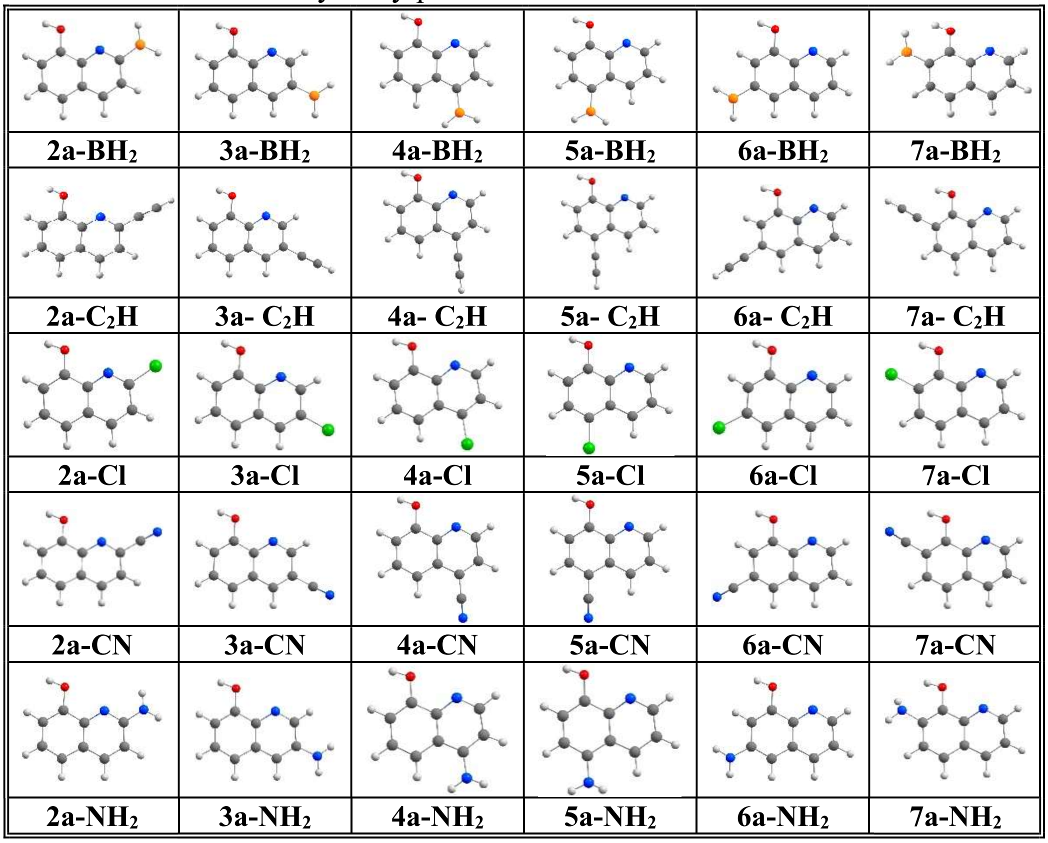

**2a-BH_2**	**3a-BH_2**	**4a-BH_2**	**5a-BH_2**	**6a-BH_2**	**7a-BH_2**
**2a-C_2H**	**3a- C_2H**	**4a- C_2H**	**5a- C_2H**	**6a- C_2H**	**7a- C_2H**
**2a-Cl**	**3a-Cl**	**4a-Cl**	**5a-Cl**	**6a-Cl**	**7a-Cl**
**2a-CN**	**3a-CN**	**4a-CN**	**5a-CN**	**6a-CN**	**7a-CN**
**2a-NH_2**	**3a-NH_2**	**4a-NH_2**	**5a-NH_2**	**6a-NH_2**	**7a-NH_2**

Scheme S1b. The 8-hydroxyquinolines with formed intramolecular H-bond

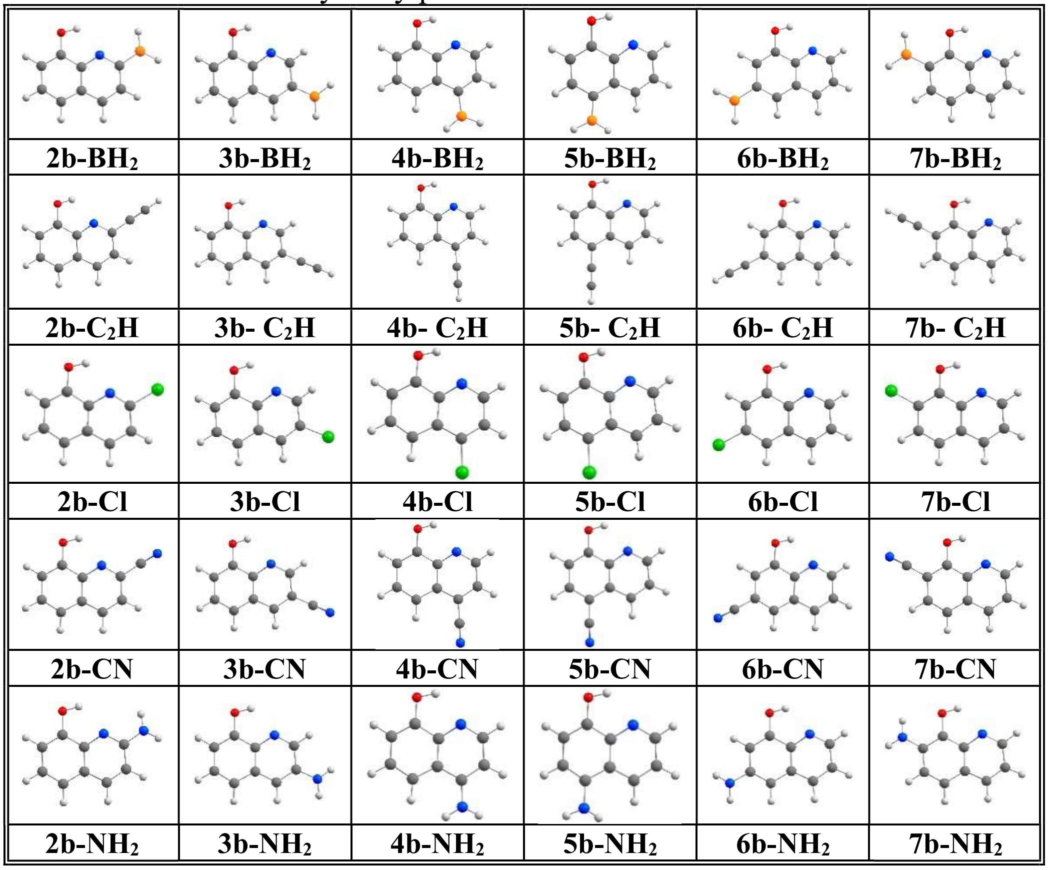

**2b-BH_2**	**3b-BH_2**	**4b-BH_2**	**5b-BH_2**	**6b-BH_2**	**7b-BH_2**
**2b-C_2H**	**3b- C_2H**	**4b- C_2H**	**5b- C_2H**	**6b- C_2H**	**7b- C_2H**
**2b-Cl**	**3b-Cl**	**4b-Cl**	**5b-Cl**	**6b-Cl**	**7b-Cl**
**2b-CN**	**3b-CN**	**4b-CN**	**5b-CN**	**6b-CN**	**7b-CN**
**2b-NH_2**	**3b-NH_2**	**4b-NH_2**	**5b-NH_2**	**6b-NH_2**	**7b-NH_2**

Scheme S1c. The 8-hydroxyquinolines in complex with a Re(I) ion.

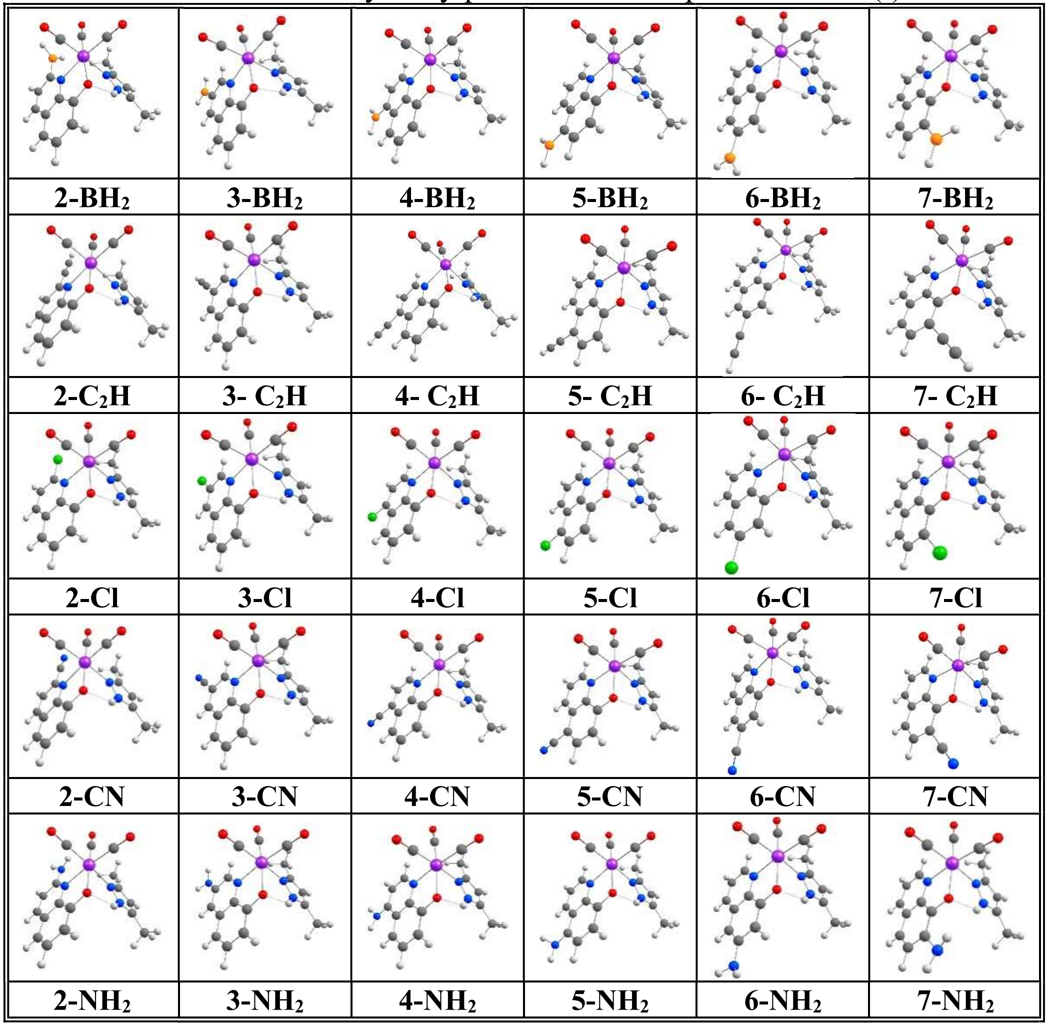

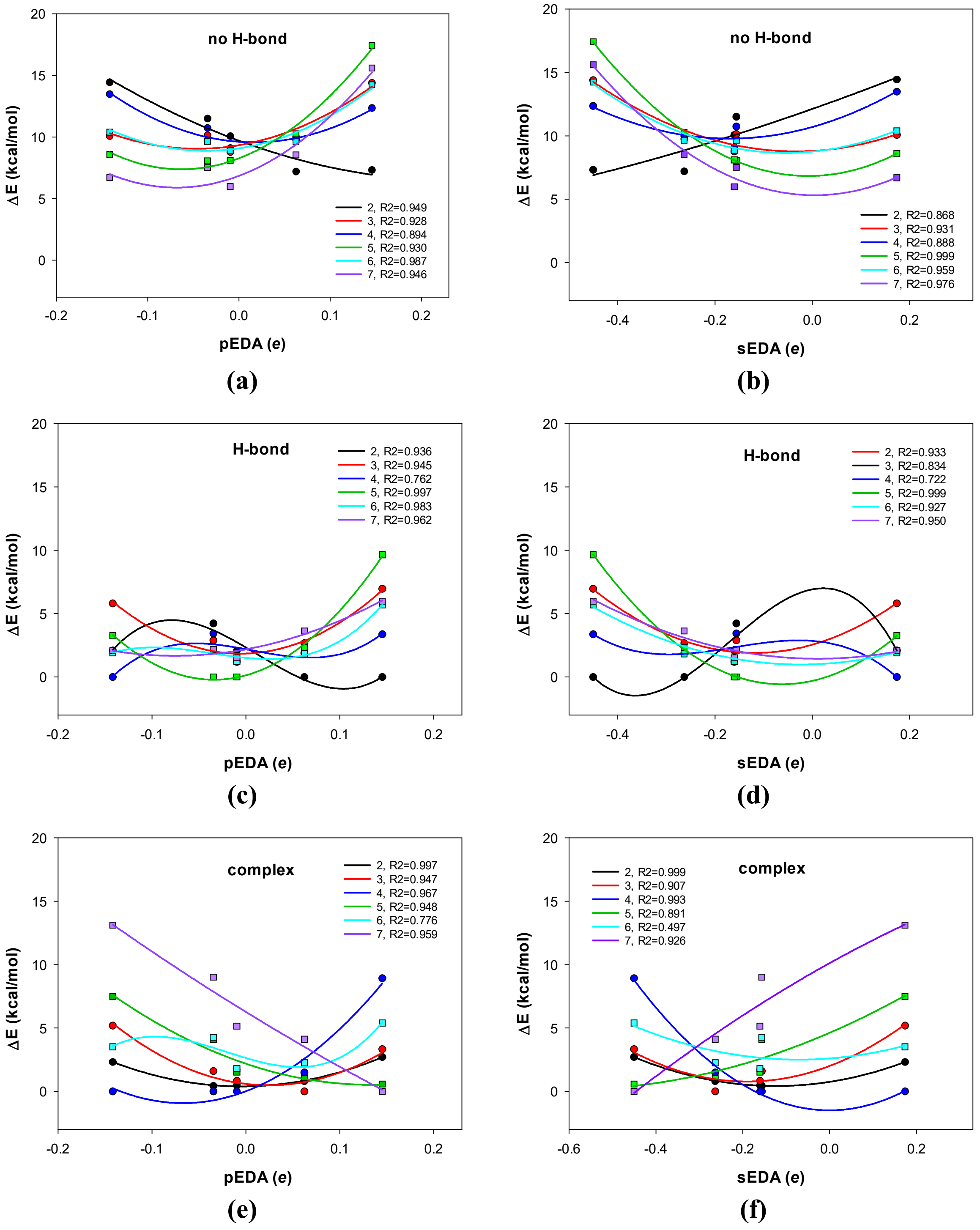

Fig. S1. Change of the 8-hydroxyquinoline's energy differences (298 K) with the pEDA and sEDA substituent effect descriptors: (a) and (b) molecule with the broken intramolecular H-bond, (c) and (d) molecule with formed intramolecular H-bond, (e) and (f) molecule as a bidentate ligand in the Re(I)$(CO)_3$L complex, where Y and L stand for a counterion and a (monodentate) ligand. Circles correspond to substitutions in pyridine's positions 2 (black), 3 (red), and 4 (blue), while squares correspond to substitutions in phenolic's positions 5 (green), 6 (light blue), and 7 (violet). The pEDA descriptor increases while sEDA decreases in the BH_2, CN, C≡CH, Cl, and NH_2 series.

Table S1. Total energies E (Hartree) and Gibbs free enthalpies in 298 K G_{298} (Hartree) and ΔE and Δ G_{298} differences (kcal/mol) concerning the most stable isomer and isomer population in a hypothetical isomer equilibrium mixture according to ΔE and Δ G_{298} pop_E and pop_{G298} (%) of the substituted 8HQ without OH...N internal hydrogen bond calculated at the B3LYP/cc-pVTZ level.

Substituent	isomer	pop_E	pop_{G298}	ΔE	Δ G_{298}	E	G_{298}
BH2	2-BH_2	0.00	0.00	14.45	13.15	-502.777807	-502.662013
	3-BH_2	0.00	0.00	10.09	9.09	-502.784764	-502.668474
	4-BH_2	0.00	0.00	13.49	12.37	-502.779343	-502.663247
	5-BH_2	0.00	0.00	8.61	8.02	-502.787123	-502.670177
	6-BH_2	0.00	0.00	10.40	9.42	-502.784270	-502.667953
	7-BH_2	0.00	0.00	6.70	6.30	-502.790167	-502.672918
C_2H	2-C_2H	0.00	0.00	10.08	9.28	-553.507639	-553.394703
	3-C_2H	0.00	0.00	8.79	8.06	-553.509699	-553.396637
	4-C_2H	0.00	0.00	9.11	8.51	-553.509184	-553.395920
	5-C_2H	0.00	0.00	8.11	7.56	-553.510780	-553.397438
	6-C_2H	0.00	0.00	8.88	8.21	-553.509549	-553.396404
	7-C_2H	0.00	0.00	5.99	5.65	-553.514165	-553.400492
CN	2-CN	0.00	0.00	11.51	10.64	-569.606802	-569.504077
	3-CN	0.00	0.00	10.15	9.36	-569.608972	-569.506102
	4-CN	0.00	0.00	10.75	10.03	-569.608011	-569.505034
	5-CN	0.00	0.00	8.08	7.55	-569.612265	-569.508992
	6-CN	0.00	0.00	9.66	8.95	-569.609747	-569.506764
	7-CN	0.00	0.00	7.53	7.08	-569.613146	-569.509738
NH_2	2-NH_2	0.00	0.00	7.33	6.81	-532.729606	-532.607383
	3-NH_2	0.00	0.00	14.40	13.68	-532.718340	-532.596437
	4-NH_2	0.00	0.00	12.36	11.91	-532.721585	-532.599266
	5-NH_2	0.00	0.00	17.43	16.79	-532.713507	-532.591477
	6-NH_2	0.00	0.00	14.25	13.59	-532.718587	-532.596580
	7-NH_2	0.00	0.00	15.62	14.90	-532.716402	-532.594488
Cl	2-Cl	0.00	0.00	7.21	6.69	-936.971696	-936.876514
	3-Cl	0.00	0.00	10.00	9.54	-936.967240	-936.871977
	4-Cl	0.00	0.00	9.77	9.47	-936.967606	-936.872079
	5-Cl	0.00	0.00	10.27	9.95	-936.966822	-936.871327
	6-Cl	0.00	0.00	9.66	9.23	-936.967785	-936.872465
	7-Cl	0.00	0.00	8.56	8.36	-936.969545	-936.873859

Table S2. Total energies E (Hartree) and Gibbs free enthalpies in 298 K G_{298} (Hartree) and ΔE and Δ G_{298} differences (kcal/mol) concerning the most stable isomer and isomer population in a hypothetical isomer equilibrium mixture according to ΔE and Δ G_{298} pop_E and pop_{G298} (%) of the substituted 8HQ with OH...N internal hydrogen bond calculated at the B3LYP/cc-pVTZ level.

Substituent	isomer	pop_E	pop_{G298}	ΔE	Δ G_{298}	E	G_{298}
BH_2	2-BH_2	0.00	0.01	5.93	5.25	-502.791389	-502.674594
	3-BH_2	2.67	5.08	2.10	1.69	-502.797488	-502.680266
	4-BH_2	0.01	0.01	5.81	5.24	-502.791586	-502.674615
	5-BH_2	93.23	88.69	0.00	0.00	-502.800840	-502.682964
	6-BH_2	0.38	0.80	3.26	2.79	-502.795646	-502.678524
	7-BH_2	3.71	5.41	1.91	1.66	-502.797799	-502.680326
C_2H	2-C_2H	2.04	2.84	2.07	1.84	-553.520405	-553.406563
	3-C_2H	9.08	11.24	1.19	1.02	-553.521813	-553.407862
	4-C_2H	8.25	8.24	1.24	1.20	-553.521722	-553.407569
	5-C_2H	67.37	63.10	0.00	0.00	-553.523703	-553.409489
	6-C_2H	8.09	9.43	1.25	1.13	-553.521704	-553.407696
	7-C_2H	5.17	5.16	1.52	1.48	-553.521282	-553.407127
CN	2-CN	0.07	0.13	4.23	3.86	-569.618401	-569.514867
	3-CN	0.70	1.07	2.90	2.64	-569.620527	-569.516819
	4-CN	0.28	0.38	3.44	3.25	-569.619653	-569.515849
	5-CN	94.01	92.29	0.00	0.00	-569.625142	-569.521025
	6-CN	2.56	3.43	2.13	1.95	-569.621743	-569.517919
	7-CN	2.38	2.70	2.18	2.09	-569.621675	-569.517693
NH_2	2-NH_2	99.65	99.70	0.00	0.00	-532.741289	-532.618240
	3-NH_2	0.00	0.00	6.97	6.80	-532.730176	-532.607398
	4-NH_2	0.34	0.28	3.37	3.47	-532.735923	-532.612715
	5-NH_2	0.00	0.00	9.65	9.60	-532.725909	-532.602941
	6-NH_2	0.01	0.01	5.68	5.62	-532.732240	-532.609289
	7-NH_2	0.00	0.00	5.99	6.11	-532.731744	-532.608511
Cl	2-Cl	89.26	91.85	0.00	0.00	-936.983182	-936.887176
	3-Cl	0.96	0.88	2.68	2.75	-936.978905	-936.882797
	4-Cl	3.75	2.48	1.88	2.14	-936.980191	-936.883768
	5-Cl	1.77	1.21	2.32	2.56	-936.979486	-936.883093
	6-Cl	4.07	3.44	1.83	1.94	-936.980269	-936.884078
	7-Cl	0.19	0.14	3.64	3.85	-936.977384	-936.881038

Table S3. Total energies E (Hartree) and Gibbs free enthalpies in 298 K G_{298} (Hartree) and ΔE and Δ G_{298} differences (kcal/mol) concerning the most stable isomer and isomer population in a hypothetical isomer equilibrium mixture according to ΔE and Δ G_{298} pop_E and pop_{G298} (%) of the substituted 8HQ in the Re(I) complex calculated at the B3LYP/cc-pVTZ level.

Substituent	isomer	pop_E	pop_{G298}	ΔE	Δ G_{298}	E	G_{298}
BH_2	2-BH_2	0.00%	0.00%	13.12	11.78	-1225.770996	-1225.534319
	3-BH_2	0.26%	0.51%	3.52	3.09	-1225.786291	-1225.548164
	4-BH_2	0.00%	0.00%	7.50	6.83	-1225.779942	-1225.542197
	5-BH_2	97.78%	94.42%	0.00	0.00	-1225.791901	-1225.553085
	6-BH_2	0.02%	0.04%	5.19	4.60	-1225.783636	-1225.545762
	7-BH_2	1.95%	5.02%	2.32	1.74	-1225.788210	-1225.550318
C_2H	2-C_2H	0.01%	0.01%	5.16	5.4	-1276.497033	-1276.262226
	3-C_2H	2.66%	2.96%	1.80	1.67	-1276.502392	-1276.268183
	4-C_2H	4.15%	3.53%	1.53	1.56	-1276.502813	-1276.268348
	5-C_2H	55.19%	49.42%	0.00	0.00	-1276.505253	-1276.270838
	6-C_2H	13.31%	14.91%	0.84	0.71	-1276.503911	-1276.269708
	7-C_2H	24.69%	29.18%	0.48	0.31	-1276.504494	-1276.270341
CN	2-CN	0.00%	0.00%	9.02	8.94	-1292.593397	-1292.36822
	3-CN	0.05%	0.07%	4.28	3.99	-1292.600948	-1292.376111
	4-CN	0.06%	0.07%	4.10	4.01	-1292.601238	-1292.37608
	5-CN	64.78%	60.75%	0.00	0.00	-1292.607775	-1292.382470
	6-CN	4.31%	5.27%	1.60	1.45	-1292.605218	-1292.380165
	7-CN	30.80%	33.83%	0.44	0.35	-1292.607074	-1292.381918
NH_2	2-NH_2	72.12%	60.90%	0.00	0.00	-1255.727564	-1255.483109
	3-NH_2	0.01%	0.01%	5.40	4.95	-1255.718960	-1255.475228
	4-NH_2	26.87%	37.95%	0.58	0.28	-1255.726633	-1255.482663
	5-NH_2	0.00%	0.00%	8.93	8.55	-1255.713333	-1255.469481
	6-NH_2	0.26%	0.46%	3.33	2.90	-1255.722251	-1255.478493
	7-NH_2	0.74%	0.68%	2.71	2.66	-1255.723250	-1255.478864
Cl	2-Cl	0.06%	0.06%	4.11	4.24	-1659.963512	-1659.746313
	3-Cl	1.44%	1.76%	2.26	2.19	-1659.966465	-1659.749578
	4-Cl	11.48%	8.67%	1.03	1.24	-1659.968425	-1659.751082
	5-Cl	5.24%	4.03%	1.49	1.70	-1659.967687	-1659.75036
	6-Cl	65.47%	70.77%	0.00	0.00	-1659.970068	-1659.753062
	7-Cl	16.31%	14.70%	0.82	0.93	-1659.968757	-1659.75158

Table S4. The sEDA and pEDA substituent effect descriptors ΔE_{298} for the 8-: molecule with the broken intramolecular H-bond, molecule with formed intramolecular H-bond, molecule as a bidentate ligand in the $Re(I)(CO)_3L$ complex

substituent	*s*EDA	*p*EDA	Isomer position					
			2	3	4	5	6	7
**(a) No H-bond**								
**BH_2**	0.17	-0.14	14.45	10.09	13.49	8.61	10.40	6.70
**CN**	-0.16	-0.04	11.51	10.15	10.75	8.08	9.66	7.53
**C_2H**	-0.16	-0.01	10.08	8.79	9.11	8.11	8.88	5.99
**Cl**	-0.26	0.06	7.21	10.00	9.77	10.27	9.66	8.56
**NH_2**	-0.45	0.15	7.33	14.40	12.36	17.43	14.25	15.62
**(b) H-bond**								
**BH_2**	0.17	-0.14	2.10	5.81	0.00	3.26	1.91	2.07
**CN**	-0.16	-0.04	4.23	2.90	3.44	0.00	2.13	2.18
**C_2H**	-0.16	-0.01	2.07	1.19	1.24	0.00	1.25	1.52
**Cl**	-0.26	0.06	0.00	2.68	1.88	2.32	1.83	3.64
**NH_2**	-0.45	0.15	0.00	6.97	3.37	9.65	5.68	5.99
**(c) complex**								
**BH_2**	0.17	-0.14	2.32	5.19	0.00	7.50	3.52	13.12
**CN**	-0.16	-0.04	0.44	1.60	0.00	4.10	4.28	9.02
**C_2H**	-0.16	-0.01	0.48	0.84	0.00	1.53	1.80	5.16
**Cl**	-0.26	0.06	0.82	0.00	1.49	1.03	2.26	4.11
**NH_2**	-0.45	0.15	2.71	3.33	8.93	0.58	5.40	0.00

Table S5. The sEDA and pEDA substituent effect descriptors relate ΔG_{298} for the 8-hydroxyquinoline: molecule with the broken intramolecular H-bond, molecule with formed intramolecular H-bond, molecule as a bidentate ligand in the $Re(I)(CO)_3L$ complex

substituent	*s*EDA	*p*EDA	Isomer position 2	3	4	5	6	7
**(a) No H-bond**								
**BH_2**	0.17	-0.14	13.15	9.09	12.37	8.02	9.42	6.30
**CN**	-0.16	-0.04	10.64	9.36	10.03	7.55	8.95	7.08
**C_2H**	-0.16	-0.01	9.28	8.06	8.51	7.56	8.21	5.65
**Cl**	-0.26	0.06	6.69	9.54	9.47	9.95	9.23	8.36
**NH_2**	-0.45	0.15	6.81	13.68	11.91	16.79	13.59	14.90
**(b) H-bond**								
**BH_2**	0.17	-0.14	1.69	5.24	0.00	2.79	1.66	1.84
**CN**	-0.16	-0.04	3.86	2.64	3.25	0.00	1.95	2.09
**C_2H**	-0.16	-0.01	1.84	1.02	1.20	0.00	1.13	1.48
**Cl**	-0.26	0.06	0.00	2.75	2.14	2.56	1.94	3.85
**NH_2**	-0.45	0.15	0.00	6.80	3.47	9.60	5.62	6.11
**(c) complex**								
**BH_2**	0.17	-0.14	1.74	4.60	0.00	6.83	3.09	11.78
**CN**	-0.16	-0.04	0.35	1.45	0.00	4.01	3.99	8.94
**C_2H**	-0.16	-0.01	0.31	0.71	0.00	1.56	1.67	5.40
**Cl**	-0.26	0.06	0.93	0.00	1.70	1.24	2.19	4.24
**NH_2**	-0.45	0.15	2.66	2.90	8.55	0.28	4.95	0.00

Table S6. Variations of the HOMA geometrical for pyridine (R1) and phenolic (R2) rings in 8-hydroxyquinolines and complex, substituted with the isomer group (Scheme 1) corresponding to the systems with the broken intra-molecular H-bond, formed intramolecular H-bond, and in the bidentate Re(I)(CO)$_3$L complex.

substituent	isomer	No H-bond		H-bond		complex	
		R1	R2	R1	R2	R1	R2
NH_2	2-NH_2	0.850	0.699	0.880	0.727	0.904	0.655
	3- NH_2	0.832	0.745	0.854	0.790	0.864	0.822
	4- NH_2	0.823	0.759	0.859	0.802	0.874	0.782
	5- NH_2	0.787	0.772	0.820	0.809	0.845	0.807
	6- NH_2	0.814	0.783	0.832	0.827	0.850	0.832
	7- NH_2	0.846	0.765	0.865	0.813	0.884	0.801
CN	2-CN	0.813	0.763	0.840	0.800	0.874	0.728
	3-CN	0.825	0.747	0.854	0.782	0.865	0.804
	4-CN	0.823	0.761	0.852	0.797	0.852	0.792
	5-CN	0.817	0.801	0.846	0.841	0.846	0.810
	6-CN	0.814	0.781	0.845	0.818	0.859	0.829
	7-CN	0.789	0.796	0.818	0.835	0.858	0.826
Cl	2-Cl	0.845	0.697	0.871	0.743	0.728	0.874
	3-Cl	0.829	0.753	0.855	0.792	0.804	0.865
	4-Cl	0.806	0.739	0.840	0.789	0.792	0.852
	5-Cl	0.797	0.765	0.832	0.808	0.810	0.846
	6-Cl	0.817	0.782	0.847	0.824	0.829	0.859
	7-Cl	0.821	0.788	0.848	0.829	0.826	0.858
C_2H	2-C_2H	0.819	0.749	0.848	0.788	0.874	0.728
	3-C_2H	0.828	0.741	0.856	0.779	0.865	0.804
	4-C_2H	0.823	0.752	0.852	0.791	0.852	0.792
	5-C_2H	0.806	0.794	0.835	0.835	0.846	0.810
	6-C_2H	0.813	0.778	0.838	0.819	0.859	0.829
	7-C_2H	0.798	0.791	0.821	0.832	0.858	0.826
BH_2	2-BH_2	0.783	0.751	0.814	0.788	0.885	0.801
	3- BH_2	0.820	0.738	0.854	0.772	0.869	0.786
	4-BH_2	0.808	0.692	0.834	0.733	0.842	0.724
	5-BH_2	0.759	0.799	0.786	0.845	0.764	0.847
	6-BH_2	0.799	0.773	0.826	0.810	0.848	0.812
	7-BH_2	0.730	0.801	0.773	0.837	0.723	0.853

Table S7. Different NICS aromaticity indices of ring in 8-hydroxyquinoline (a) no intramolecular H-bond, (b) formed intrtamolecular H-bond and (c) complex with a Re(I) ion (Scheme 1), S - Substituent type, Ring - pyridine (R1) and phenolic (R2) rings in 8-HQs, isomer - substitution in the positions, I – integral NICS (INICS. ppm·Å), I- – integral NICS for the distances below the plane (ppm·Å), I+ – integral NICS for the distances below the plane (ppm·Å), ΔI=I(-) – I(+)(ppm·Å); NICS(0), NICS(1). NICS(-1), and ΔNICS(-1,1)= NICS(-1) - NICS(1) (ppm), MIN1 and MIN2 are minima of the NICS function for distances below and above the ring plane, respectively, and NICS(MIN1) and NICS(MIN2) are the NICS function values in MIN1 and MIN2.

(a) no intramolecular H-bond														
**S**	**Ring**	**isomer**	**I**	**I-**	**I+**	**ΔI**	**NICS(0)**	**NICS(-1)**	**NICS(1)**	**ΔNICS(-1.1)**	**MIN1**	**NICS(MIN1)**	**MIN2**	**NICS(MIN2)**
**BH_2**	**R1**	2-BH_2	-148.8	-74.4	-74.4	0.0	-11.7	-28.2	-28.2	0.0	-1.0	-28.2	1.0	-28.2
		3-BH_2	-138.5	-69.3	-69.3	0.0	-9.1	-25.7	-25.7	0.0	-1.1	-25.7	1.1	-25.7
		4-BH_2	-137.1	-68.6	-68.6	0.0	-8.5	-25.6	-25.6	0.0	-1.1	-25.6	1.1	-25.6
		5-BH_2	-146.9	-73.5	-73.5	0.0	-12.0	-28.3	-28.3	0.0	-1.0	-28.3	1.0	-28.3
		6-BH_2	-143.9	-71.9	-71.9	0.0	-11.4	-27.5	-27.5	0.0	-1.0	-27.5	1.0	-27.5
		7-BH_2	-139.8	-69.9	-69.9	0.0	-10.8	-26.9	-26.9	0.0	-1.0	-26.9	1.0	-26.9
	**R2**	2-BH_2	-137.5	-68.7	-68.7	0.0	-11.1	-25.4	-25.4	0.0	-1.0	-25.4	1.0	-25.4
		3-BH_2	-141.5	-70.7	-70.7	0.0	-12.2	-26.4	-26.4	0.0	-1.0	-26.4	1.0	-26.4
		4-BH_2	-145.2	-72.6	-72.6	0.0	-13.1	-27.3	-27.3	0.0	-1.0	-27.3	1.0	-27.3
		5-BH_2	-127.9	-64.0	-64.0	0.0	-7.4	-22.9	-22.9	0.0	-1.1	-23.1	1.1	-23.1
		6-BH_2	-140.1	-70.0	-70.0	0.0	-10.9	-25.6	-25.6	0.0	-1.0	-25.6	1.0	-25.6
		7-BH_2	-116.9	-58.4	-58.4	0.0	-4.6	-20.6	-20.6	0.0	-1.1	-20.9	1.1	-20.9
**C_2H**	**R1**	2-C_2H	-137.2	-68.6	-68.6	0.0	-9.9	-26.0	-26.0	0.0	-1.0	-26.0	1.0	-26.0
		3-C_2H	-137.1	-68.6	-68.6	0.0	-10.1	-25.9	-25.9	0.0	-1.0	-25.9	1.0	-25.9
		4-C_2H	-137.1	-68.5	-68.5	0.0	-9.5	-25.8	-25.8	0.0	-1.0	-25.8	1.0	-25.8
		5-C_2H	-147.6	-73.8	-73.8	0.0	-12.2	-28.3	-28.3	0.0	-1.0	-28.3	1.0	-28.3
		6-C_2H	-142.4	-71.2	-71.2	0.0	-11.2	-27.3	-27.3	0.0	-1.0	-27.3	1.0	-27.3
		7-C_2H	-143.2	-71.6	-71.6	0.0	-11.4	-27.5	-27.5	0.0	-1.0	-27.5	1.0	-27.5
	**R2**	2-C_2H	-139.5	-69.8	-69.8	0.0	-11.9	-26.1	-26.1	0.0	-1.0	-26.1	1.0	-26.1
		3-C_2H	-140.3	-70.2	-70.2	0.0	-12.1	-26.3	-26.3	0.0	-1.0	-26.3	1.0	-26.3
		4-C_2H	-142.0	-71.0	-71.0	0.0	-12.2	-26.5	-26.5	0.0	-1.0	-26.5	1.0	-26.5
		5-C_2H	-132.4	-66.2	-66.2	0.0	-9.7	-24.1	-24.1	0.0	-1.0	-24.1	1.0	-24.1
		6-C_2H	-134.4	-67.2	-67.2	0.0	-10.8	-24.8	-24.8	0.0	-1.0	-24.8	1.0	-24.8
		7-C_2H	-131.9	-66.0	-66.0	0.0	-9.3	-24.1	-24.1	0.0	-1.0	-24.1	1.0	-24.1
**CN**	**R1**	2-CN	-141.7	-70.9	-70.9	0.0	-11.2	-27.0	-27.0	0.0	-1.0	-27.0	1.0	-27.0
		3-CN	-138.7	-69.4	-69.4	0.0	-10.5	-26.3	-26.3	0.0	-1.0	-26.3	1.0	-26.3
		4-CN	-139.6	-69.8	-69.8	0.0	-10.3	-26.5	-26.5	0.0	-1.0	-26.5	1.0	-26.5
		5-CN	-148.0	-74.0	-74.0	0.0	-12.4	-28.5	-28.5	0.0	-1.0	-28.5	1.0	-28.5
		6-CN	-144.6	-72.3	-72.3	0.0	-11.8	-27.8	-27.8	0.0	-1.0	-27.8	1.0	-27.8
		7-CN	-144.6	-72.3	-72.3	0.0	-11.8	-27.8	-27.8	0.0	-1.0	-27.8	1.0	-27.8
	**R2**	2-CN	-141.0	-70.5	-70.5	0.0	-12.2	-26.4	-26.4	0.0	-1.0	-26.4	1.0	-26.4
		3-CN	-142.4	-71.2	-71.2	0.0	-12.6	-26.8	-26.8	0.0	-1.0	-26.8	1.0	-26.8
		4-CN	-143.3	-71.7	-71.7	0.0	-12.6	-26.9	-26.9	0.0	-1.0	-26.9	1.0	-26.9
		5-CN	-132.0	-66.0	-66.0	0.0	-9.7	-24.1	-24.1	0.0	-1.0	-24.1	1.0	-24.1
		6-CN	-136.0	-68.0	-68.0	0.0	-11.1	-25.1	-25.1	0.0	-1.0	-25.1	1.0	-25.1
		7-CN	-129.7	-64.9	-64.9	0.0	-8.9	-23.6	-23.6	0.0	-1.1	-23.6	1.1	-23.6

S	Ring	isomer	I	I-	I+	ΔI	NICS(0)	NICS(-1)	NICS(1)	ΔNICS(-1.1)	MIN1	NICS(MIN1)	MIN2	NICS(MIN2)
NH_2	R1	2-NH_2	-114.7	-57.6	-57.1	-0.6	-4.0	-21.5	-21.2	-0.3	-1.1	-21.6	1.1	-21.4
		3-NH_2	-129.8	-65.3	-64.5	-0.8	-8.6	-25.0	-24.7	-0.4	-1.0	-25.0	1.0	-24.7
		4-NH_2	-125.8	-63.3	-62.5	-0.8	-7.0	-23.8	-23.4	-0.5	-1.1	-23.9	1.1	-23.4
		5-NH_2	-145.9	-72.4	-73.6	1.2	-11.8	-27.6	-28.3	0.7	-1.0	-27.6	1.0	-28.3
		6-NH_2	-134.9	-67.5	-67.4	-0.1	-9.6	-26.0	-26.0	0.0	-1.0	-26.0	1.0	-26.0
		7-NH_2	-142.8	-71.5	-71.3	-0.2	-11.1	-27.3	-27.1	-0.1	-1.0	-27.3	1.0	-27.1
	R2	2-NH_2	-135.6	-67.8	-67.8	0.0	-11.3	-25.5	-25.5	0.0	-1.0	-25.5	1.0	-25.5
		3-NH_2	-132.9	-66.5	-66.4	-0.1	-10.3	-24.8	-24.8	0.0	-1.0	-24.8	1.0	-24.8
		4-NH_2	-137.0	-68.2	-68.8	0.6	-11.2	-25.3	-25.6	0.4	-1.0	-25.3	1.0	-25.6
		5-NH_2	-131.3	-66.0	-65.3	-0.6	-9.9	-24.3	-23.8	-0.5	-1.0	-24.3	1.0	-23.8
		6-NH_2	-121.1	-60.8	-60.2	-0.6	-7.8	-22.5	-22.2	-0.3	-1.0	-22.5	1.0	-22.2
		7-NH_2	-143.6	-72.1	-71.4	-0.7	-12.9	-26.9	-26.5	-0.4	-1.0	-26.9	1.0	-26.5
Cl	R1	2-Cl	-133.8	-66.9	-66.9	0.0	-10.2	-25.0	-25.0	0.0	-1.0	-25.0	1.0	-25.0
		3-Cl	-138.6	-69.3	-69.3	0.0	-11.0	-26.2	-26.2	0.0	-1.0	-26.2	1.0	-26.2
		4-Cl	-140.1	-70.0	-70.0	0.0	-11.1	-26.3	-26.3	0.0	-1.0	-26.3	1.0	-26.3
		5-Cl	-148.7	-74.4	-74.4	0.0	-12.6	-28.4	-28.4	0.0	-1.0	-28.4	1.0	-28.4
		6-Cl	-143.3	-71.6	-71.6	0.0	-11.6	-27.5	-27.5	0.0	-1.0	-27.5	1.0	-27.5
		7-Cl	-145.4	-72.7	-72.7	0.0	-12.0	-27.8	-27.8	0.0	-1.0	-27.8	1.0	-27.8
	R2	2-Cl	-142.2	-71.1	-71.1	0.0	-12.8	-26.8	-26.8	0.0	-1.0	-26.8	1.0	-26.8
		3-Cl	-141.0	-70.5	-70.5	0.0	-12.5	-26.4	-26.4	0.0	-1.0	-26.4	1.0	-26.4
		4-Cl	-141.8	-70.9	-70.9	0.0	-12.2	-26.3	-26.3	0.0	-1.0	-26.3	1.0	-26.3
		5-Cl	-136.1	-68.1	-68.1	0.0	-11.5	-24.8	-24.8	0.0	-1.0	-24.8	1.0	-24.8
		6-Cl	-134.1	-67.1	-67.1	0.0	-11.6	-24.7	-24.7	0.0	-1.0	-24.7	1.0	-24.7
		7-Cl	-138.2	-69.1	-69.1	0.0	-11.5	-25.3	-25.3	0.0	-1.0	-25.3	1.0	-25.3

(b) formed intrtamolecular H-bond

S	Ring	isomer	I	I-	I+	ΔI	NICS(0)	NICS(-1)	NICS(1)	ΔNICS(-1.1)	MIN1	NICS(MIN1)	MIN2	NICS(MIN2)
BH_2 (b)	R1	2-BH_2	-148.6	-74.3	-74.3	0.0	-11.1	-28.0	-28.0	0.0	-1.0	-28.0	1.0	-28.0
		3-BH_2	-137.1	-68.6	-68.6	0.0	-8.2	-25.3	-25.3	0.0	-1.1	-25.4	1.1	-25.4
		4-BH_2	-137.5	-68.8	-68.8	0.0	-8.1	-25.6	-25.6	0.0	-1.1	-25.6	1.1	-25.6
		5-BH_2	-146.3	-73.1	-73.1	0.0	-11.3	-28.0	-28.0	0.0	-1.0	-28.0	1.0	-28.0
		6-BH_2	-143.1	-71.6	-71.6	0.0	-10.6	-27.2	-27.2	0.0	-1.0	-27.2	1.0	-27.2
		7-BH_2	-141.5	-70.7	-70.7	0.0	-10.6	-27.1	-27.1	0.0	-1.0	-27.1	1.0	-27.1
	R2	2-BH_2	-136.6	-68.3	-68.3	0.0	-9.9	-25.2	-25.2	0.0	-1.0	-25.2	1.0	-25.2
		3-BH_2	-142.5	-71.2	-71.2	0.0	-11.7	-26.7	-26.7	0.0	-1.0	-26.7	1.0	-26.7
		4-BH_2	-145.8	-72.9	-72.9	0.0	-12.5	-27.4	-27.4	0.0	-1.0	-27.4	1.0	-27.4
		5-BH_2	-127.0	-63.5	-63.5	0.0	-6.3	-22.7	-22.7	0.0	-1.1	-22.9	1.1	-22.9
		6-BH_2	-142.1	-71.0	-71.0	0.0	-10.7	-26.0	-26.0	0.0	-1.0	-26.0	1.0	-26.0
		7-BH_2	-123.6	-61.8	-61.8	0.0	-6.0	-22.1	-22.1	0.0	-1.1	-22.3	1.1	-22.3
C_2H	R1	2-C_2H	-136.9	-68.4	-68.4	0.0	-9.3	-25.8	-25.8	0.0	-1.0	-25.8	1.0	-25.8
		3-C_2H	-136.4	-68.2	-68.2	0.0	-9.4	-25.7	-25.7	0.0	-1.0	-25.7	1.0	-25.7
		4-C_2H	-136.9	-68.4	-68.4	0.0	-9.0	-25.7	-25.7	0.0	-1.0	-25.7	1.0	-25.7
		5-C_2H	-147.0	-73.5	-73.5	0.0	-11.6	-28.1	-28.1	0.0	-1.0	-28.1	1.0	-28.1
		6-C_2H	-141.5	-70.7	-70.7	0.0	-10.5	-27.0	-27.0	0.0	-1.0	-27.0	1.0	-27.0
		7-C_2H	-142.5	-71.3	-71.3	0.0	-10.8	-27.2	-27.2	0.0	-1.0	-27.2	1.0	-27.2
	R2	2-C_2H	-139.9	-70.0	-70.0	0.0	-11.2	-26.2	-26.2	0.0	-1.0	-26.2	1.0	-26.2

		3-C_2H	-141.1	-70.6	-70.6	0.0	-11.7	-26.5	-26.5	0.0	-1.0	-26.5	1.0	-26.5
		4-C_2H	-142.2	-71.1	-71.1	0.0	-11.5	-26.5	-26.5	0.0	-1.0	-26.5	1.0	-26.5
		5-C_2H	-132.4	-66.2	-66.2	0.0	-8.9	-24.2	-24.2	0.0	-1.1	-24.2	1.1	-24.2
		6-C_2H	-135.1	-67.6	-67.6	0.0	-10.2	-25.0	-25.0	0.0	-1.0	-25.0	1.0	-25.0
		7-C_2H	-133.2	-66.6	-66.6	0.0	-9.5	-24.4	-24.4	0.0	-1.0	-24.4	1.0	-24.4
CN	R1	2-CN	-141.4	-70.7	-70.7	0.0	-10.6	-26.8	-26.8	0.0	-1.0	-26.8	1.0	-26.8
		3-CN	-137.7	-68.9	-68.9	0.0	-9.7	-25.9	-25.9	0.0	-1.0	-25.9	1.0	-25.9
		4-CN	-139.5	-69.8	-69.8	0.0	-9.9	-26.3	-26.3	0.0	-1.0	-26.3	1.0	-26.3
		5-CN	-147.3	-73.7	-73.7	0.0	-11.8	-28.2	-28.2	0.0	-1.0	-28.2	1.0	-28.2
		6-CN	-143.6	-71.8	-71.8	0.0	-11.0	-27.5	-27.5	0.0	-1.0	-27.5	1.0	-27.5
		7-CN	-144.1	-72.1	-72.1	0.0	-11.2	-27.6	-27.6	0.0	-1.0	-27.6	1.0	-27.6
	R2	2-CN	-141.8	-70.9	-70.9	0.0	-11.6	-26.6	-26.6	0.0	-1.0	-26.6	1.0	-26.6
		3-CN	-143.7	-71.9	-71.9	0.0	-12.3	-27.0	-27.0	0.0	-1.0	-27.0	1.0	-27.0
		4-CN	-144.1	-72.0	-72.0	0.0	-12.0	-27.0	-27.0	0.0	-1.0	-27.0	1.0	-27.0
		5-CN	-131.7	-65.9	-65.9	0.0	-8.9	-24.1	-24.1	0.0	-1.1	-24.1	1.1	-24.1
		6-CN	-137.0	-68.5	-68.5	0.0	-10.7	-25.4	-25.4	0.0	-1.0	-25.4	1.0	-25.4
		7-CN	-131.2	-65.6	-65.6	0.0	-9.1	-24.0	-24.0	0.0	-1.0	-24.0	1.0	-24.0
NH_2	R1	2-NH_2	-113.0	-56.8	-56.2	-0.6	-3.0	-20.9	-20.7	-0.3	-1.1	-21.2	1.1	-20.9
		3-NH_2	-130.1	-65.5	-64.6	-0.8	-8.2	-25.0	-24.6	-0.4	-1.0	-25.0	1.0	-24.6
		4-NH_2	-123.3	-62.0	-61.3	-0.7	-5.7	-23.2	-22.8	-0.4	-1.1	-23.3	1.1	-22.9
		5-NH_2	-145.0	-71.9	-73.1	1.2	-11.1	-27.2	-28.0	0.7	-1.0	-27.2	1.0	-28.0
		6-NH_2	-133.7	-66.9	-66.8	-0.1	-8.7	-25.6	-25.6	0.0	-1.0	-25.6	1.0	-25.6
		7-NH_2	-135.7	-67.9	-67.8	-0.1	-8.9	-25.7	-25.6	0.0	-1.0	-25.7	1.0	-25.6
	R2	2-NH_2	-136.6	-68.3	-68.3	0.0	-10.8	-25.7	-25.7	0.0	-1.0	-25.7	1.0	-25.7
		3-NH_2	-133.0	-66.6	-66.4	-0.1	-9.6	-24.8	-24.7	0.0	-1.0	-24.8	1.0	-24.7
		4-NH_2	-136.4	-68.0	-68.4	0.4	-10.3	-25.2	-25.4	0.2	-1.0	-25.2	1.0	-25.4
		5-NH_2	-133.2	-66.9	-66.3	-0.6	-9.9	-24.7	-24.3	-0.4	-1.0	-24.7	1.0	-24.3
		6-NH_2	-120.1	-60.4	-59.7	-0.7	-6.8	-22.3	-22.0	-0.3	-1.1	-22.4	1.1	-22.1
		7-NH_2	-139.1	-69.9	-69.2	-0.6	-11.5	-26.2	-26.0	-0.3	-1.0	-26.2	1.0	-26.0
Cl	R1	2-Cl	-133.5	-66.7	-66.7	0.0	-9.5	-24.9	-24.9	0.0	-1.0	-24.9	1.0	-24.9
		3-Cl	-138.2	-69.1	-69.1	0.0	-10.4	-26.0	-26.0	0.0	-1.0	-26.0	1.0	-26.0
		4-Cl	-139.1	-69.6	-69.6	0.0	-10.3	-26.0	-26.0	0.0	-1.0	-26.0	1.0	-26.0
		5-Cl	-147.9	-74.0	-74.0	0.0	-11.9	-28.1	-28.1	0.0	-1.0	-28.1	1.0	-28.1
		6-Cl	-142.5	-71.3	-71.3	0.0	-10.8	-27.3	-27.3	0.0	-1.0	-27.3	1.0	-27.3
		7-Cl	-144.2	-72.1	-72.1	0.0	-11.1	-27.5	-27.5	0.0	-1.0	-27.5	1.0	-27.5
	R2	2-Cl	-143.1	-71.6	-71.6	0.0	-12.4	-27.0	-27.0	0.0	-1.0	-27.0	1.0	-27.0
		3-Cl	-141.6	-70.8	-70.8	0.0	-11.8	-26.6	-26.6	0.0	-1.0	-26.6	1.0	-26.6
		4-Cl	-142.0	-71.0	-71.0	0.0	-11.5	-26.4	-26.4	0.0	-1.0	-26.4	1.0	-26.4
		5-Cl	-136.9	-68.5	-68.5	0.0	-11.1	-25.0	-25.0	0.0	-1.0	-25.0	1.0	-25.0
		6-Cl	-134.6	-67.3	-67.3	0.0	-11.0	-24.8	-24.8	0.0	-1.0	-24.8	1.0	-24.8
		7-Cl	-138.9	-69.4	-69.4	0.0	-11.4	-25.5	-25.5	0.0	-1.0	-25.5	1.0	-25.5

(c) coplex

S	Ring	isomer	I	I-	I+	ΔI	NICS(0)	NICS(-1)	NICS(1)	ΔNICS(-1.1)	MIN1	NICS(MIN1)	MIN2	NICS(MIN2)
BH_2 (b)	R1	2-BH_2	-111.0	-54.7	-56.2	-3.6	-19.8	-19.7	-1.1	-20.1	1.1	-20.0	0.0	-3.6
		3-BH_2	-140.3	-69.1	-71.2	-11.1	-26.1	-26.2	-1.0	-26.1	1.0	-26.2	0.0	-11.1

		4-BH_2	-118.7	-58.3	-60.5	-5.4	-21.4	-21.4	-1.1	-21.6	1.1	-21.6	0.0	-5.4
		5-BH_2	-143.5	-70.6	-72.9	-13.0	-27.5	-27.5	-1.0	-27.5	1.0	-27.5	0.0	-13.0
		6-BH_2	-139.8	-68.9	-70.9	-12.0	-26.6	-26.6	-1.0	-26.6	1.0	-26.6	0.0	-12.0
		7-BH_2	-138.9	-68.2	-70.8	-11.7	-26.3	-26.5	-1.0	-26.3	1.0	-26.5	0.0	-11.7
	R2	2-BH_2	-123.9	-61.2	-62.7	-6.9	-23.4	-23.4	-1.1	-23.4	1.0	-23.4	0.0	-6.9
		3-BH_2	-126.2	-62.2	-63.9	-6.7	-23.3	-23.4	-1.1	-23.4	1.1	-23.5	0.0	-6.7
		4-BH_2	-130.5	-64.3	-66.3	-8.0	-24.6	-24.7	-1.0	-24.6	1.0	-24.7	0.0	-8.0
		5-BH_2	-116.5	-57.4	-59.1	-3.2	-21.0	-21.1	-1.1	-21.3	1.1	-21.4	0.0	-3.2
		6-BH_2	-119.6	-59.0	-60.6	-4.1	-21.5	-21.6	-1.1	-21.8	1.1	-21.8	0.0	-4.1
		7-BH_2	-123.9	-60.8	-63.1	-6.3	-23.1	-23.4	-1.1	-23.1	1.1	-23.4	0.0	-6.3
C_2H	R1	2-C_2H	-133.1	-65.7	-67.5	-10.5	-24.9	-24.8	-1.0	-24.9	1.0	-24.8	0.0	-10.5
		3-C_2H	-133.1	-65.5	-67.6	-11.1	-25.0	-25.0	-1.0	-25.0	1.0	-25.0	0.0	-11.1
		4-C_2H	-131.5	-64.6	-66.9	-10.1	-24.4	-24.5	-1.0	-24.4	1.0	-24.5	0.0	-10.1
		5-C_2H	-138.9	-68.4	-70.6	-11.7	-26.2	-26.3	-1.0	-26.2	1.0	-26.3	0.0	-11.7
		6-C_2H	-138.7	-68.3	-70.4	-11.9	-26.3	-26.4	-1.0	-26.3	1.0	-26.4	0.0	-11.9
		7-C_2H	-138.8	-68.7	-70.0	-12.0	-26.5	-26.4	-1.0	-26.5	1.0	-26.4	0.0	-12.0
	R2	2-C_2H	-127.4	-62.9	-64.5	-7.4	-23.8	-23.9	-1.1	-23.8	1.1	-23.9	0.0	-7.4
		3-C_2H	-126.1	-62.1	-63.9	-7.1	-23.6	-23.7	-1.1	-23.6	1.1	-23.7	0.0	-7.1
		4-C_2H	-131.2	-64.7	-66.5	-8.1	-24.5	-24.6	-1.0	-24.5	1.0	-24.6	0.0	-8.1
		5-C_2H	-123.3	-60.7	-62.5	-6.3	-22.6	-22.8	-1.1	-22.7	1.1	-22.8	0.0	-6.3
		6-C_2H	-122.7	-60.3	-62.3	-6.6	-22.5	-22.7	-1.1	-22.6	1.1	-22.7	0.0	-6.6
		7-C_2H	-119.9	-59.7	-60.3	-5.8	-22.0	-21.9	-1.1	-22.1	1.1	-22.0	0.0	-5.8
CN	R1	2-CN	-128.8	-63.5	-65.3	-9.7	-24.0	-23.9	-1.0	-24.0	1.0	-23.9	0.0	-9.7
		3-CN	-133.4	-65.6	-67.8	-11.3	-25.1	-25.1	-1.0	-25.1	1.0	-25.1	0.0	-11.3
		4-CN	-128.7	-63.2	-65.5	-9.6	-23.9	-23.9	-1.0	-23.9	1.0	-23.9	0.0	-9.6
		5-CN	-140.2	-69.0	-71.2	-12.1	-26.7	-26.8	-1.0	-26.7	1.0	-26.8	0.0	-12.1
		6-CN	-140.3	-69.1	-71.3	-12.3	-26.8	-26.9	-1.0	-26.8	1.0	-26.9	0.0	-12.3
		7-CN	-139.4	-69.0	-70.4	-12.2	-26.6	-26.6	-1.0	-26.6	1.0	-26.6	0.0	-12.2
	R2	2-CN	-127.8	-63.1	-64.7	-7.6	-24.0	-24.1	-1.1	-24.0	1.0	-24.1	0.0	-7.6
		3-CN	-127.3	-62.7	-64.5	-7.3	-23.9	-23.9	-1.1	-23.9	1.1	-23.9	0.0	-7.3
		4-CN	-131.4	-64.8	-66.6	-8.4	-24.7	-24.8	-1.0	-24.7	1.0	-24.8	0.0	-8.4
		5-CN	-122.1	-60.2	-62.0	-6.3	-22.5	-22.6	-1.1	-22.6	1.1	-22.6	0.0	-6.3
		6-CN	-121.3	-59.6	-61.6	-6.4	-22.2	-22.4	-1.1	-22.3	1.1	-22.5	0.0	-6.4
		7-CN	-120.4	-59.9	-60.5	-6.1	-22.2	-22.1	-1.1	-22.3	1.1	-22.2	0.0	-6.1
NH_2	R1	2-NH_2	-142.8	-69.9	-72.9	-12.9	-27.2	-27.5	-1.0	-27.2	1.0	-27.5	0.0	-12.9
		3-NH_2	-115.9	-56.4	-59.4	-6.7	-21.6	-22.0	-1.0	-21.6	1.0	-22.0	0.0	-6.7
		4-NH_2	-132.9	-65.1	-67.7	-10.5	-24.6	-25.2	-1.0	-24.6	1.0	-25.2	0.0	-10.5
		5-NH_2	-131.4	-64.7	-66.7	-9.8	-24.7	-24.5	-1.0	-24.7	1.0	-24.5	0.0	-9.8
		6-NH_2	-129.1	-63.4	-65.7	-9.5	-24.2	-24.3	-1.0	-24.2	1.0	-24.3	0.0	-9.5
		7-NH_2	-132.5	-65.2	-67.3	-10.8	-25.3	-25.2	-1.0	-25.3	1.0	-25.2	0.0	-10.8
	R2	2-NH_2	-119.2	-58.5	-60.7	-4.9	-21.8	-21.9	-1.1	-22.0	1.1	-22.1	0.0	-4.9
		3-NH_2	-117.6	-57.8	-59.8	-5.2	-22.1	-22.2	-1.1	-22.2	1.1	-22.3	0.0	-5.2
		4-NH_2	-128.6	-63.9	-64.6	-7.3	-24.2	-23.7	-1.1	-24.3	1.1	-23.8	0.0	-7.3
		5-NH_2	-109.6	-53.7	-55.9	-2.6	-19.6	-20.1	-1.1	-19.9	1.1	-20.3	0.0	-2.6
		6-NH_2	-116.7	-57.1	-59.7	-5.2	-21.6	-22.1	-1.1	-21.7	1.1	-22.1	0.0	-5.2

		7-NH_2	-96.6	-47.3	-49.3	1.0	-17.0	-17.1	-1.2	-17.6	1.2	-17.7	0.0	1.0
**Cl**	**R1**	2-Cl	-136.7	-67.4	-69.3	-11.5	-25.5	-25.4	-1.0	-25.5	1.0	-25.4	0.0	-11.5
		3-Cl	-129.8	-63.8	-66.0	-10.7	-24.2	-24.2	-1.0	-24.2	1.0	-24.2	0.0	-10.7
		4-Cl	-132.0	-64.9	-67.1	-10.5	-24.4	-24.5	-1.0	-24.4	1.0	-24.5	0.0	-10.5
		5-Cl	-137.5	-67.6	-69.9	-11.2	-25.8	-25.9	-1.0	-25.8	1.0	-25.9	0.0	-11.2
		6-Cl	-137.4	-67.6	-69.8	-11.6	-26.0	-26.1	-1.0	-26.0	1.0	-26.1	0.0	-11.6
		7-Cl	-137.4	-68.1	-69.3	-11.9	-26.2	-26.1	-1.0	-26.2	1.0	-26.1	0.0	-11.9
	**R2**	2-Cl	-127.9	-63.1	-64.8	-7.5	-23.8	-23.9	-1.1	-23.8	1.0	-23.9	0.0	-7.5
		3-Cl	-125.4	-61.8	-63.6	-7.0	-23.5	-23.6	-1.1	-23.5	1.1	-23.6	0.0	-7.0
		4-Cl	-131.3	-64.8	-66.6	-8.1	-24.4	-24.6	-1.0	-24.4	1.0	-24.6	0.0	-8.1
		5-Cl	-123.3	-60.7	-62.6	-6.6	-22.5	-22.6	-1.1	-22.5	1.1	-22.6	0.0	-6.6
		6-Cl	-122.4	-60.2	-62.2	-6.9	-22.4	-22.6	-1.1	-22.4	1.0	-22.6	0.0	-6.9
		7-Cl	-117.0	-58.3	-58.7	-5.5	-21.3	-21.1	-1.1	-21.4	1.1	-21.2	0.0	-5.5

(A)

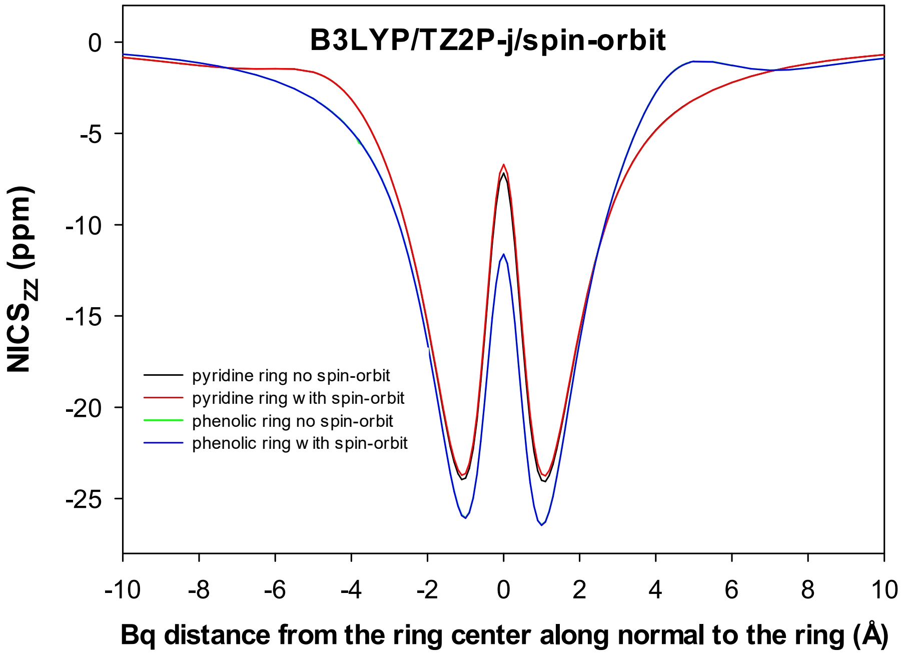

(B)

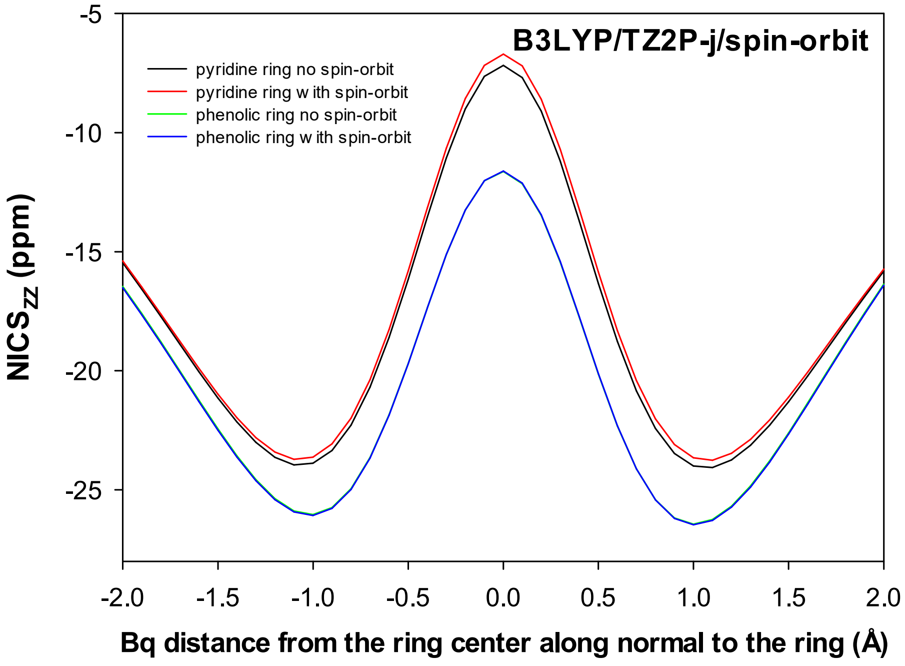

Fig. S2. The $NICS_{ZZ}$ scans for the 8-hydroxyquinoline ligand complexed with the tricarbonyl Rhenium moiety calculated at the B3LYP/TZ2p-j level with and without spin-orbit effect included (see legend). Although, the spin-orbit effect on the aromaticity of the system is colossal (Table S8), in the case when the Rhenium atom is only coordinated with the ligand, the spin-orbit impact on the magnetic aromaticity of the 8-hydroxyquinoline ligand rings is negligible.

Table S8. The ZZ elements of the shielding tensors (ppm) for the probe points calculated for the 8-hydroxyquinoline ligand complexed with tricarbonyl Rhenium moiety calculated at the B3LYP/TZ2p-j level with and without spin-orbit effect included.

TOTAL SHIELDING TENSOR FOR **RE** ATOM IN THE COMPLEX **WITHOUT SPIN-ORBIT CORRECTION**	TOTAL SHIELDING TENSOR FOR **RE** ATOM IN THE COMPLEX **WITH SPIN-ORBIT CORRECTION**
----------------------------------- 1413.567 127.869 -247.218 127.869 1203.615 -136.800 -247.218 -136.800 1663.301	----------------------------------- 4500.747 120.627 -284.076 120.627 4173.389 -129.313 -284.076 -129.313 4720.144

distance from the ring center	pyridine ring		phenolic ring	
	no SO	SO	no SO	SO
-10.0	0.8390	0.8410	0.6670	0.6670
-9.5	0.9410	0.9440	0.7560	0.7580
-9.0	1.0520	1.0550	0.8620	0.8640
-8.5	1.1670	1.1710	0.9870	0.9890
-8.0	1.2800	1.2850	1.1360	1.1390
-7.5	1.3780	1.3840	1.3150	1.3190
-7.0	1.4450	1.4520	1.5320	1.5370
-6.5	1.4680	1.4760	1.7980	1.8050
-6.0	1.4560	1.4670	2.1290	2.1380
-5.5	1.4740	1.4870	2.5490	2.5610
-5.0	1.6480	1.6620	3.0960	3.1130
-4.9	1.7150	1.7300	3.2260	3.2430
-4.8	1.7970	1.8120	3.3630	3.3810
-4.7	1.8950	1.9100	3.5090	3.5280
-4.6	2.0100	2.0250	3.6640	3.6850
-4.5	2.1430	2.1580	3.8300	3.8520
-4.4	2.2970	2.3110	4.0070	4.0300
-4.3	2.4710	2.4860	4.1960	4.2210
-4.2	2.6690	2.6830	4.3990	4.4250
-4.1	2.8900	2.9030	4.6240	4.6410
-4.0	3.1360	3.1490	4.8550	4.8760
-3.9	3.4090	3.4200	5.1010	5.1290
-3.8	3.7080	3.7190	5.5110	5.3660
-3.7	4.0350	4.0450	5.6580	5.6920
-3.6	4.3910	4.4000	5.9710	6.0060
-3.5	4.7780	4.7840	6.3090	6.3450
-3.4	5.1950	5.2000	6.6750	6.7120
-3.3	5.6450	5.6480	7.0700	7.1090
-3.2	6.1300	6.1300	7.4980	7.5380
-3.1	6.6500	6.6480	7.9620	8.0030
-3.0	7.2090	7.2030	8.4650	8.5080

-2.9	7.8080	7.7990	9.0100	9.0550
-2.8	8.4510	8.4370	9.6030	9.6490
-2.7	9.1400	9.1210	10.2460	10.2940
-2.6	9.8790	9.8540	10.9430	10.9930
-2.5	10.6690	10.6380	11.6990	11.7500
-2.4	11.5140	11.4760	12.5170	12.5690
-2.3	12.4160	12.3690	13.4000	13.4530
-2.2	13.3750	13.3190	14.3500	14.4040
-2.1	14.3910	14.3230	15.3670	15.4220
-2.0	15.4590	15.3800	16.4490	16.5040
-1.9	16.5730	16.4800	17.5900	17.6460
-1.8	17.7200	17.6130	18.7800	18.8360
-1.7	18.8830	18.7600	20.0030	20.0580
-1.6	20.0350	19.8950	21.2330	21.2880
-1.5	21.1400	20.9810	22.4360	22.4900
-1.4	22.1500	21.9720	23.5670	23.6190
-1.3	23.0040	22.8070	24.5670	24.6170
-1.2	23.6330	23.4150	25.3660	25.4120
-1.1	23.9540	23.7160	25.8840	25.9260
-1.0	23.8840	23.6260	26.0360	26.0750
-0.9	23.3460	23.0690	25.7450	25.7790
-0.8	22.2860	21.9900	24.9530	24.9830
-0.7	20.6890	20.3740	23.6410	23.6660
-0.6	18.6050	18.2700	21.8490	21.8700
-0.5	16.1580	15.8010	19.6910	19.7070
-0.4	13.5530	13.1730	17.3610	17.3700
-0.3	11.0650	10.6590	15.1160	15.1190
-0.2	8.9990	8.5680	13.2470	13.2440
-0.1	7.6370	7.1810	12.0230	12.0120
0.0	7.1780	6.7010	11.6330	11.6150
0.1	7.6920	7.1990	12.1420	12.1190
0.2	9.1020	8.6010	13.4770	13.4500
0.3	11.2040	10.7020	15.4400	15.4120
0.4	13.7130	13.2190	17.7570	17.7310
0.5	16.3250	15.8450	20.1350	20.1150
0.6	18.7680	18.3090	22.3180	22.3060
0.7	20.8410	20.4070	24.1140	24.1120
0.8	22.4230	22.0180	25.4140	25.4220
0.9	23.4700	23.0960	26.1810	26.1990
1.0	23.9980	23.6560	26.4380	26.4660
1.1	24.0640	23.7550	26.2450	26.2810
1.2	23.7460	23.4680	25.6830	25.7260
1.3	23.1270	22.8800	24.8380	24.8850
1.4	22.2880	22.0710	23.7910	23.8410
1.5	21.3010	21.1110	22.6120	22.6630
1.6	20.2260	20.0610	21.3590	21.4110

1.7	19.1080	18.9670	20.0780	20.1310
1.8	17.9850	17.8640	18.8040	18.8560
1.9	16.8820	16.7800	17.5610	17.6110
2.0	15.8170	15.7330	16.3640	16.4120
2.1	14.8020	14.7330	15.2230	15.2700
2.2	13.8440	13.7890	14.1440	14.1890
2.3	12.9470	12.9030	13.1280	13.1710
2.4	12.1110	12.0760	12.1740	12.2150
2.5	11.3340	11.3090	11.2790	11.3190
2.6	10.6150	10.5980	10.4410	10.4790
2.7	9.9510	9.9400	9.6550	9.6910
2.8	9.3390	9.3330	8.9160	8.9510
2.9	8.7750	8.7730	8.2210	8.2550
3.0	8.2550	8.2570	7.5660	7.5980
3.1	7.7750	7.7810	6.9470	6.9780
3.2	7.3340	7.3420	6.3620	6.3920
3.3	6.9270	6.9380	5.8070	5.8370
3.4	6.5520	6.5640	5.2830	5.3110
3.5	6.2050	6.2180	4.7880	4.8150
3.6	5.8850	5.8990	4.3210	4.3470
3.7	5.5880	5.6030	3.8830	3.9090
3.8	5.3140	5.3290	3.4750	3.5000
3.9	5.0590	5.0740	3.0970	3.1210
4.0	4.8220	4.8380	2.7510	2.7750
4.1	4.6020	4.6180	2.4380	2.4610
4.2	4.3970	4.4120	2.1580	2.1800
4.3	4.2060	4.2200	1.9120	1.9340
4.4	4.0270	4.0410	1.7000	1.7210
4.5	3.8590	3.8730	1.5200	1.5410
4.6	3.7020	3.7150	1.3720	1.3920
4.7	3.5540	3.5670	1.2540	1.2730
4.8	3.4140	3.4270	1.1630	1.1820
4.9	3.2830	3.2950	1.0960	1.1150
5.0	3.1590	3.1700	1.0520	1.0710
5.5	2.6290	2.6370	1.0690	1.0840
6.0	2.2130	2.2180	1.2710	1.2830
6.5	1.8780	1.8820	1.4620	1.4710
7.0	1.6040	1.6060	1.5460	1.5530
7.5	1.3770	1.3790	1.5190	1.5240
8.0	1.1890	1.1900	1.4180	1.4230
8.5	1.0310	1.0320	1.2840	1.2880
9.0	0.8990	0.8990	1.1450	1.1480
9.5	0.7870	0.7870	1.0120	1.0140
10.0	0.6920	0.6920	0.8920	0.8940

Table S9. Cartesian xyz coordinates for optimized molecules for: No H-bond, H-bond and complex with a Re(I) ion using B3LYP/aug-cc-pVTZ empiricaldispersion=GD3.

**No H-bond**			
2-BH2	2-C2H	2-Cl	2-CN
C 2.164854 -0.495513 0.000000	C -0.954281 1.621402 -0.000000	C -0.714816 -1.607950 -0.000000	C -1.004219 1.594689 -0.000000
C 0.000000 0.322844 -0.000000	C -0.000000 -0.465375 0.000000	C -0.000000 0.549897 0.000000	C 0.000000 -0.458278 0.000000
C -0.571659 -0.990530 -0.000000	C 1.324216 0.067874 0.000000	C 1.368807 0.155793 0.000000	C 1.307341 0.119164 0.000000
C 0.330074 -2.076154 0.000000	C 1.441118 1.476916 -0.000000	C 1.630866 -1.235640 0.000000	C 1.383037 1.530297 -0.000000
C 1.675747 -1.833123 0.000000	C 0.319541 2.254271 -0.000000	C 0.599779 -2.130863 -0.000000	C 0.238417 2.276276 -0.000000
C -0.888289 1.449100 -0.000000	C -0.157276 -1.886289 0.000000	C -0.309582 1.941159 0.000000	C -0.117087 -1.883465 0.000000
C -1.974475 -1.155600 -0.000000	C 2.442127 -0.795383 0.000000	C 2.390747 1.130694 0.000000	C 2.450369 -0.709964 0.000000
H -0.058859 -3.086789 0.000000	H 2.425869 1.926482 0.000000	H 2.656499 -1.581611 0.000000	H 2.353596 2.008905 -0.000000
H 2.385272 -2.649204 0.000000	H 0.376953 3.332922 -0.000000	H 0.761526 -3.197520 -0.000000	H 0.260253 3.356113 -0.000000
C -2.787342 -0.052667 -0.000000	C 2.251215 -2.152314 0.000000	C 2.058073 2.460387 0.000000	C 2.298277 -2.071860 0.000000
C -2.249053 1.249472 -0.000000	C 0.953709 -2.699022 0.000000	C 0.710884 2.866628 0.000000	C 1.019174 -2.660181 0.000000
H -2.391670 -2.153561 -0.000000	H 3.438219 -0.373670 0.000000	H 3.425402 0.815688 0.000000	H 3.433511 -0.259590 0.000000
H -3.862440 -0.171539 -0.000000	H 3.100733 -2.821538 0.000000	H 2.832222 3.215344 0.000000	H 3.167727 -2.714875 0.000000
H -2.914711 2.104184 -0.000000	H 0.825942 -3.774914 0.000000	H 0.470128 3.922922 0.000000	H 0.926507 -3.739395 0.000000
N 1.325737 0.545620 0.000000	N -1.105945 0.310385 -0.000000	N -1.015830 -0.352124 -0.000000	N -1.126953 0.284747 -0.000000
O -0.321563 2.678109 -0.000000	O -1.425243 -2.366196 0.000000	O -1.622077 2.281262 0.000000	O -1.369058 -2.395783 0.000000
H -1.009766 3.351416 -0.000000	H -1.405847 -3.328570 0.000000	H -1.709166 3.239832 0.000000	H -1.329293 -3.357738 0.000000
H 4.460788 -1.100041 0.000000	C -2.133203 2.433218 -0.000000	Cl -2.044572 -2.763560 -0.000000	C -2.218590 2.369934 -0.000000
H 4.066360 0.928745 0.000000	C -3.096881 3.149499 -0.000000		N -3.158781 3.034278 -0.000000
B 3.683647 -0.194878 0.000000	H -3.960017 3.767373 -0.000000		
2-NH2	3-BH2	3-C2H	3-Cl
C 2.123657 0.229653 -0.004668	C 2.225639 0.041510 0.000000	C 1.922608 0.429225 0.000000	C 1.842630 0.465801 0.000000
C -0.171887 0.293113 -0.001198	C 0.000000 0.559490 -0.000000	C 0.000000 -0.813103 0.000000	C 0.000000 -0.898049 0.000000
C -0.282038 -1.126068 0.000940	C -0.381734 -0.816455 -0.000000	C -0.823592 0.351502 0.000000	C -0.898511 0.209695 -0.000000
C 0.935749 -1.852857 0.000784	C 0.660194 -1.763762 0.000000	C -0.172196 1.601317 0.000000	C -0.329419 1.503770 -0.000000
C 2.128243 -1.197474 -0.005627	C 1.987430 -1.367569 0.000000	C 1.204178 1.662016 0.000000	C 1.030278 1.623646 -0.000000
C -1.375845 1.055562 -0.000227	H 3.251809 0.395503 0.000000	H 3.006584 0.456112 0.000000	H 2.921338 0.571265 0.000000
C -1.548122 -1.750927 0.002999	C -1.030876 1.545119 -0.000000	C -0.631587 -2.091656 0.000000	C -0.546423 -2.215855 0.000000
H 0.903797 -2.935125 0.001865	C -1.749184 -1.182576 -0.000000	C -2.233172 0.228353 0.000000	C -2.296072 -0.004931 -0.000000
H 3.065833 -1.737049 -0.018253	H 0.410511 -2.818502 0.000000	H -0.758488 2.510530 0.000000	H -0.965271 2.377860 -0.000000
C -2.685800 -0.985687 0.002869	C -2.710359 -0.208150 -0.000000	C -2.803810 -1.016474 0.000000	C -2.782871 -1.285217 -0.000000
C -2.597301 0.418710 0.001491	C -2.351916 1.153976 -0.000000	C -2.006243 -2.176942 0.000000	C -1.912146 -2.391794 0.000000
H -1.605295 -2.831507 0.003967	H -2.016449 -2.230711 -0.000000	H -2.843639 1.121190 0.000000	H -2.964224 0.845392 -0.000000
H -3.660990 -1.452656 0.003851	H -3.758587 -0.473647 -0.000000	H -3.880122 -1.121711 0.000000	H -3.850077 -1.459807 -0.000000
H -3.504152 1.012389 0.001597	H -3.128457 1.909812 -0.000000	H -2.479136 -3.151718 0.000000	H -2.320719 -3.394903 0.000000
N 1.019400 0.939812 0.002556	N 1.297212 0.962992 0.000000	N 1.355336 -0.746601 0.000000	N 1.346883 -0.744076 0.000000
O -1.250411 2.411839 -0.000764	O -0.644826 2.845130 -0.000000	O 0.177138 -3.180843 0.000000	O 0.333343 -3.247744 0.000000
H -2.125816 2.811269 -0.003078	H -1.421616 3.413508 -0.000000	H -0.358928 -3.980275 0.000000	H -0.147443 -4.081714 0.000000
N 3.319153 0.914568 -0.058913	H 4.268257 -2.010373 0.000000	C 1.909400 2.896561 0.000000	Cl 1.799216 3.193529 -0.000000
H 3.255422 1.898733 0.144192	H 2.896178 -3.549113 0.000000	C 2.525045 3.928633 0.000000	
H 4.144689 0.454424 0.282302	B 3.136265 -2.381592 0.000000	H 3.065499 4.842236 0.000000	

3-CN	3-NH2	4-BH2	4-C2H
C 1.943444 0.413404 0.000000	C -1.751288 -1.270398 -0.006281	C 2.279183 0.883496 -0.000000	C -2.083180 -1.307197 -0.000000
C -0.000000 -0.797999 0.000000	C 0.389398 -0.459247 -0.001043	C 0.000000 0.653857 -0.000000	C 0.170326 -0.924108 0.000000
C -0.810538 0.377149 0.000000	C -0.070431 0.890933 -0.002438	C 0.073684 -0.776108 0.000000	C -0.000000 0.493224 0.000000
C -0.142874 1.617416 0.000000	C -1.464622 1.104487 -0.003253	C 1.381396 -1.373356 0.000000	C -1.343836 0.980861 -0.000000
C 1.232844 1.647311 0.000000	C -2.326602 0.032647 -0.005566	C 2.466070 -0.510968 0.000000	C -2.377928 0.067921 -0.000000
H 3.027326 0.426042 0.000000	H -2.410932 -2.135014 -0.011733	H 3.135814 1.549054 -0.000000	H -2.895625 -2.025924 -0.000000
C -0.648156 -2.068818 0.000000	C 1.792901 -0.705920 0.002235	C -1.282417 1.284956 -0.000000	C 1.500260 -1.446939 0.000000
C -2.221078 0.274756 0.000000	C 0.866266 1.952258 -0.000509	C -1.125013 -1.524645 0.000000	C 1.126706 1.344289 0.000000
H -0.713381 2.536371 0.000000	H -1.845727 2.118954 -0.005707	H 3.472269 -0.908818 0.000000	H -3.404752 0.403780 -0.000000
C -2.806751 -0.962943 0.000000	C 2.208407 1.676400 0.002191	C -2.337970 -0.884910 0.000000	C 2.385786 0.803523 0.000000
C -2.024056 -2.133267 0.000000	C 2.678105 0.349243 0.003620	C -2.423842 0.518083 -0.000000	C 2.576842 -0.590194 0.000000
H -2.819404 1.175504 0.000000	H 0.510573 2.973983 -0.002073	H -1.081289 -2.602075 0.000000	H 0.982120 2.414555 0.000000
H -3.884103 -1.054871 0.000000	H 2.927207 2.484851 0.003010	H -3.251712 -1.463872 0.000000	H 3.252321 1.450635 0.000000
H -2.510626 -3.101149 0.000000	H 3.743759 0.154355 0.005583	H -3.394228 0.999908 -0.000000	H 3.581727 -0.995204 0.000000
N 1.355949 -0.751984 0.000000	N -0.468673 -1.509269 -0.001554	N 1.095897 1.449446 -0.000000	N -0.865889 -1.796815 -0.000000
O 0.146411 -3.165636 0.000000	O 2.194041 -2.004090 0.003790	O -1.303460 2.641348 -0.000000	O 1.630354 -2.797425 0.000000
H -0.395722 -3.961171 0.000000	H 3.155784 -2.039266 0.004133	H -2.215958 2.947612 -0.000000	H 2.564373 -3.029917 0.000000
C 1.950189 2.880372 0.000000	N -3.707939 0.168576 -0.064374	H 2.795979 -3.254493 0.000000	C -1.613847 2.377981 -0.000000
N 2.542117 3.869137 0.000000	H -4.245631 -0.615207 0.267642	H 0.799917 -3.709683 0.000000	C -1.842862 3.557434 -0.000000
	H -4.083891 1.052493 0.236580	B 1.661813 -2.891393 0.000000	H -2.051382 4.598404 -0.000000

4-Cl	4-CN	4-NH2	5-BH2
C -1.792838 -1.648581 -0.000000	C -2.094900 -1.286483 -0.000000	C -1.313431 -2.047524 -0.018902	C 2.084000 1.643007 0.000000
C 0.375180 -0.932581 0.000000	C 0.164328 -0.907911 0.000000	C 0.419164 -0.573039 -0.000429	C -0.000000 0.705145 0.000000
C -0.000000 0.449402 0.000000	C -0.000000 0.510740 0.000000	C -0.449857 0.558647 -0.008638	C 0.510117 -0.625459 0.000000
C -1.395825 0.701692 -0.000000	C -1.343157 0.987444 -0.000000	C -1.856817 0.296100 0.000869	C 1.918822 -0.745243 0.000000
C -2.292371 -0.330364 -0.000000	C -2.387178 0.090775 -0.000000	C -2.269062 -1.022558 -0.011821	C 2.703091 0.377582 0.000000
H -2.491567 -2.478192 -0.000000	H -2.907196 -2.004487 -0.000000	H -1.649312 -3.079579 -0.027123	H 2.688629 2.543660 0.000000
C 1.767924 -1.252859 0.000000	C 1.492357 -1.435841 0.000000	C 1.828823 -0.336779 0.011271	C -1.419253 0.895105 0.000000
C 0.992107 1.455335 0.000000	C 1.125563 1.361816 0.000000	C 0.091135 1.865396 -0.027484	C -0.377649 -1.761949 0.000000
H -3.355786 -0.144635 -0.000000	H -3.411577 0.433322 -0.000000	H -3.322957 -1.269725 -0.010664	H 2.366372 -1.727408 0.000000
C 2.315609 1.101955 0.000000	C 2.382071 0.814568 0.000000	C 1.449645 2.049258 -0.019813	H 3.782052 0.304920 0.000000
C 2.707801 -0.248338 0.000000	C 2.569654 -0.579494 0.000000	C 2.321818 0.948205 0.004671	C -1.743697 -1.490764 0.000000
H 0.698694 2.493336 0.000000	H 0.987348 2.433482 0.000000	H -0.554795 2.730641 -0.070718	C -2.264951 -0.195121 0.000000
H 3.077931 1.868895 0.000000	H 3.250484 1.458802 0.000000	H 1.862128 3.048673 -0.038897	H -2.436119 -2.322297 0.000000
H 3.760934 -0.502496 0.000000	H 3.573945 -0.985329 0.000000	H 3.393297 1.109538 0.011652	H -3.337455 -0.039499 0.000000
N -0.518006 -1.949941 -0.000000	N -0.876928 -1.772954 -0.000000	N -0.013727 -1.857524 -0.006307	N 0.783235 1.810310 0.000000
O 2.093619 -2.569182 0.000000	O 1.616109 -2.784398 0.000000	O 2.633865 -1.429967 0.026810	O -1.868298 2.164995 0.000000
H 3.051558 -2.663478 0.000000	H 2.548302 -3.024915 0.000000	H 3.554069 -1.148015 0.029685	H -2.831671 2.169409 0.000000
Cl -1.995894 2.344450 -0.000000	C -1.604947 2.392580 -0.000000	N -2.769090 1.329913 -0.025444	H -0.770210 -4.071802 0.000000
	N -1.807775 3.526547 -0.000000	H -3.720704 1.093713 0.198326	H 1.209434 -3.555133 0.000000
		H -2.481448 2.221522 0.337166	B 0.065963 -3.221558 0.000000

5-C2H	5-Cl	5-CN	5-NH2
C -2.746338 0.538059 -0.000000	C -2.728556 0.872839 0.000000	C -2.741705 0.515103 0.000000	C 2.628537 0.505719 0.021147
C -0.776485 -0.625706 -0.000000	C -0.926616 -0.539894 0.000000	C -0.757619 -0.620979 -0.000000	C 0.342540 0.684242 0.013950
C -0.000000 0.569462 0.000000	C 0.000000 0.550014 0.000000	C 0.000000 0.584026 0.000000	C 0.174889 -0.736797 -0.005751

C -0.708080 1.791352 0.000000	C -0.545680 1.853565 0.000000	C -0.720927 1.798760 0.000000	C 1.353289 -1.514068 -0.045835
C -2.076631 1.779579 -0.000000	C -1.903785 2.017082 0.000000	C -2.088743 1.766067 0.000000	C 2.578428 -0.901694 -0.030604
H -3.830881 0.509593 -0.000000	H -3.807616 0.984521 0.000000	H -3.825442 0.472215 0.000000	H 3.588275 1.011651 0.042625
C -0.101033 -1.884400 -0.000000	C -0.425993 -1.877420 0.000000	C -0.062201 -1.871031 -0.000000	C -0.813104 1.518236 -0.002111
C 1.428860 0.499987 0.000000	C 1.388897 0.265010 0.000000	C 1.425995 0.521243 0.000000	C -1.133203 -1.307951 0.003654
H -0.154882 2.720213 0.000000	H 0.117785 2.705576 0.000000	H -0.183536 2.737482 0.000000	H 1.290735 -2.591342 -0.112061
H -2.644959 2.699293 0.000000	H -2.347721 3.002706 0.000000	H -2.669613 2.677626 0.000000	H 3.493505 -1.476226 -0.066479
C 2.031395 -0.742953 0.000000	C 1.840852 -1.025390 0.000000	C 2.055911 -0.706271 -0.000000	C -2.215626 -0.459734 -0.011680
C 1.275655 -1.922971 -0.000000	C 0.930839 -2.097650 0.000000	C 1.316778 -1.894655 -0.000000	C -2.056752 0.938617 -0.026292
H 3.110189 -0.806905 0.000000	H 2.902231 -1.224469 0.000000	H 3.135487 -0.751959 -0.000000	H -3.217888 -0.869473 -0.007944
H 1.783914 -2.879542 -0.000000	H 1.311991 -3.111280 0.000000	H 1.836418 -2.844523 -0.000000	H -2.939584 1.566664 -0.040724
N -2.131582 -0.620374 -0.000000	N -2.267981 -0.354520 0.000000	N -2.111457 -0.635155 -0.000000	N 1.564057 1.271665 0.035370
O -0.869881 -2.998530 -0.000000	O -1.339874 -2.878874 0.000000	O -0.815051 -2.990249 -0.000000	O -0.613678 2.867176 0.006263
H -0.307019 -3.779612 -0.000000	H -0.887867 -3.728694 0.000000	H -0.247394 -3.768335 -0.000000	H -1.465900 3.313339 -0.004399
C 2.214305 1.685288 0.000000	Cl 2.560371 1.575020 0.000000	C 2.192007 1.722482 0.000000	N -1.290636 -2.703970 -0.039693
C 2.869629 2.693871 0.000000		N 2.791101 2.708159 0.000000	H -0.666279 -3.220401 0.561085
H 3.456103 3.578414 0.000000			H -2.241379 -3.004916 0.109193
6-BH2	6-C2H	6-Cl	6-CN
C 1.738896 2.197682 0.000000	C 1.515713 -2.733061 -0.000000	C -2.629855 1.852687 -0.000000	C 1.523553 -2.705327 -0.000000
C -0.000000 0.713408 0.000000	C -0.000000 -1.021366 -0.000000	C -1.098167 0.155936 -0.000000	C -0.000000 -1.001606 -0.000000
C 0.885833 -0.406273 0.000000	C 1.027478 -0.032016 -0.000000	C -0.000000 1.064856 -0.000000	C 1.024271 -0.009636 -0.000000
C 2.273151 -0.131574 0.000000	C 2.363582 -0.496238 -0.000000	C -0.310962 2.444509 -0.000000	C 2.362968 -0.465787 -0.000000
C 2.703993 1.166261 0.000000	C 2.611186 -1.841302 -0.000000	C -1.620218 2.840667 -0.000000	C 2.615780 -1.809476 -0.000000
H 2.062058 3.233428 0.000000	H 1.694221 -3.803172 -0.000000	H -3.673160 2.150540 -0.000000	H 1.706123 -3.774549 -0.000000
C -1.408403 0.464655 0.000000	C -1.359422 -0.580273 0.000000	C -0.814489 -1.244352 -0.000000	C -1.364462 -0.571653 0.000000
C 0.367637 -1.717552 0.000000	C 0.702470 1.340277 0.000000	C 1.331787 0.589577 0.000000	C 0.693065 1.361504 -0.000000
H 2.974845 -0.956004 0.000000	H 3.173124 0.222441 -0.000000	H 0.493448 3.168624 -0.000000	H 3.168869 0.256693 -0.000000
H 3.756475 1.412937 0.000000	H 3.620558 -2.228410 -0.000000	H -1.891618 3.886947 -0.000000	H 3.626488 -2.192610 -0.000000
C -0.999112 -1.949998 0.000000	C -0.618109 1.737830 0.000000	C 1.547069 -0.759508 0.000000	C -0.630788 1.738452 0.000000
C -1.873610 -0.826405 0.000000	C -1.648864 0.761769 0.000000	C 0.488492 -1.686762 0.000000	C -1.663650 0.768989 0.000000
H 1.056141 -2.553138 0.000000	H 1.492202 2.078301 0.000000	H 2.158622 1.284239 0.000000	H 1.475889 2.106461 -0.000000
H -2.943772 -1.001647 0.000000	H -2.679752 1.091700 0.000000	H 0.709213 -2.745234 0.000000	H -2.694870 1.096381 0.000000
N 0.442192 1.993229 0.000000	N 0.260548 -2.350072 -0.000000	N -2.389502 0.563347 -0.000000	N 0.266616 -2.328462 -0.000000
O -2.222169 1.551081 0.000000	O -2.317829 -1.539342 0.000000	O -1.873215 -2.088404 -0.000000	O -2.311382 -1.537363 0.000000
H -3.140530 1.262399 0.000000	H -3.187764 -1.127150 0.000000	H -1.564090 -3.000172 -0.000000	H -3.187838 -1.138605 0.000000
H -2.717401 -3.587796 0.000000	C -0.966343 3.119357 0.000000	Cl 3.183404 -1.383929 0.000000	C -0.982824 3.124015 0.000000
H -0.785268 -4.305879 0.000000	C -1.270791 4.281955 0.000000		N -1.276770 4.238217 0.000000
B -1.542169 -3.385357 0.000000	H -1.535207 5.309946 0.000000		
6-NH2	7-BH2	7-C2H	7-Cl
C 2.864811 0.277568 0.005858	C -1.246677 2.469072 0.000000	C -0.516243 -2.990991 0.000000	C 0.438080 3.093483 -0.000000
C 0.589636 0.506685 0.000648	C -0.000000 0.551398 0.000000	C 0.000000 -0.763093 0.000000	C -0.000000 0.849401 -0.000000
C 0.376728 -0.904313 -0.003168	C -1.157533 -0.275817 -0.000000	C -1.373730 -0.383721 -0.000000	C 1.386853 0.519379 0.000000
C 1.536283 -1.715309 -0.002109	C -2.410086 0.376715 -0.000000	C -2.326058 -1.426370 -0.000000	C 2.302009 1.595447 0.000000
C 2.773909 -1.131037 0.002244	C -2.458844 1.744568 0.000000	C -1.903706 -2.728241 -0.000000	C 1.834103 2.881197 0.000000
H 3.837903 0.756758 0.009431	H -1.266810 3.553558 0.000000	H -0.163284 -4.016836 0.000000	H 0.048586 4.106038 -0.000000
C -0.564737 1.349221 -0.000916	C 1.287773 -0.077570 0.000000	C 0.991817 0.263155 0.000000	C -0.963272 -0.207720 -0.000000

C -0.928005 -1.439344 -0.003470	C -1.028130 -1.689653 -0.000000	C -1.735522 0.985953 -0.000000	C 1.795443 -0.834603 0.000000
H 1.428811 -2.792740 -0.005277	H -3.316137 -0.215551 -0.000000	H -3.380284 -1.180032 -0.000000	H 3.364202 1.386183 0.000000
H 3.675327 -1.728533 0.002646	H -3.401991 2.273405 0.000000	H -2.607521 -3.548868 -0.000000	H 2.507799 3.726589 0.000000
C -2.022194 -0.601399 -0.005257	C 0.210774 -2.253448 -0.000000	C -0.768699 1.948214 -0.000000	C 0.860394 -1.832371 0.000000
C -1.823610 0.802192 -0.005837	C 1.412544 -1.476668 -0.000000	C 0.608528 1.602013 0.000000	C -0.508561 -1.511358 -0.000000
H -1.060811 -2.514031 -0.006955	H -1.921431 -2.300206 -0.000000	H -2.782691 1.256745 -0.000000	H 2.850187 -1.073388 0.000000
H -2.688363 1.456613 -0.019970	H 0.311719 -3.330651 -0.000000	H -1.032024 2.996290 -0.000000	H 1.151562 -2.872435 0.000000
N 1.818921 1.071020 0.005286	N -0.062919 1.904792 0.000000	N 0.404467 -2.056793 0.000000	N -0.449519 2.127479 -0.000000
O -0.350366 2.688960 0.000802	O 2.343978 0.745738 0.000000	O 2.282984 -0.113352 0.000000	O -2.266592 0.127550 -0.000000
H -1.194564 3.150924 -0.009603	H 3.150171 0.211220 0.000000	H 2.840298 0.677941 0.000000	H -2.800667 -0.678010 -0.000000
N -3.323044 -1.093309 -0.065486	H 3.792762 -1.540449 0.000000	C 1.620586 2.599072 0.000000	Cl -1.688976 -2.813226 -0.000000
H -3.441388 -2.054480 0.209230	H 2.845330 -3.349978 -0.000000	C 2.524257 3.394506 0.000000	
H -4.042049 -0.495754 0.307520	B 2.767214 -2.162474 -0.000000	H 3.302995 4.116146 0.000000	
7-CN	7-NH2		
C -0.549559 -2.968508 0.000000	C -2.579919 -1.151290 0.015752		
C 0.000000 -0.750406 0.000000	C -0.407281 -0.433350 0.005513		
C -1.365665 -0.347303 -0.000000	C -0.803734 0.939235 -0.003525		
C -2.335004 -1.374865 -0.000000	C -2.190234 1.208104 0.003628		
C -1.932596 -2.682625 -0.000000	C -3.081280 0.169650 0.014320		
H -0.212704 -3.999427 0.000000	H -3.272332 -1.986927 0.021024		
C 1.012415 0.259028 0.000000	C 0.980677 -0.738366 -0.009534		
C -1.709460 1.028275 -0.000000	C 0.177116 1.955300 -0.026362		
H -3.385047 -1.112200 -0.000000	H -2.529504 2.236471 -0.000357		
H -2.649422 -3.491793 -0.000000	H -4.148582 0.341504 0.020520		
C -0.730941 1.976507 -0.000000	C 1.506663 1.624804 -0.010746		
C 0.637502 1.599194 -0.000000	C 1.921994 0.278097 0.010272		
H -2.752441 1.313566 -0.000000	H -0.132024 2.991838 -0.042435		
H -0.975576 3.028800 -0.000000	H 2.261432 2.401505 -0.006213		
N 0.385197 -2.048382 0.000000	N -1.303826 -1.452993 0.011875		
O 2.291199 -0.144781 0.000000	O 1.368638 -2.036970 -0.046444		
H 2.887958 0.616292 0.000000	H 2.305376 -2.047529 -0.291989		
C 1.667998 2.580326 0.000000	N 3.292025 -0.103872 -0.005918		
N 2.556161 3.317706 0.000000	H 3.909840 0.660909 -0.234724		
	H 3.595296 -0.517051 0.868121		
**H-bond**			
2-BH2	2-C2H	2-Cl	2-CN
C 2.079984 -0.708428 -0.000000	C -0.829917 1.666757 0.000000	C -0.625073 -1.627840 0.000000	C -0.888846 1.642327 0.000000
C -0.000000 0.308499 0.000000	C 0.000000 -0.474842 -0.000000	C -0.000000 0.560466 0.000000	C 0.000000 -0.465508 -0.000000
C -0.705948 -0.931987 -0.000000	C 1.353827 -0.039385 -0.000000	C 1.382968 0.240555 0.000000	C 1.339290 0.021735 -0.000000
C 0.086886 -2.099119 -0.000000	C 1.558227 1.359155 0.000000	C 1.706932 -1.136893 0.000000	C 1.496958 1.425372 -0.000000
C 1.452526 -1.986713 -0.000000	C 0.485421 2.206916 0.000000	C 0.715053 -2.079174 0.000000	C 0.395955 2.238840 0.000000
C -0.731612 1.539337 0.000000	C -0.291043 -1.871119 -0.000000	C -0.411396 1.921493 0.000000	C -0.241465 -1.871888 -0.000000
C -2.116894 -0.918937 -0.000000	C 2.389150 -0.999050 -0.000000	C 2.334234 1.283877 0.000000	C 2.408220 -0.899073 -0.000000
H -0.394176 -3.069175 -0.000000	H 2.567840 1.749643 0.000000	H 2.746131 -1.439692 0.000000	H 2.492579 1.849392 -0.000000
H 2.076823 -2.869411 -0.000000	H 0.613710 3.279234 0.000000	H 0.927641 -3.136732 0.000000	H 0.484467 3.315075 0.000000
C -2.784216 0.282865 0.000000	C 2.073453 -2.336376 -0.000000	C 1.906129 2.589466 0.000000	C 2.139721 -2.246763 -0.000000

C -2.104345 1.518049 0.000000<br>H -2.659330 -1.854494 -0.000000<br>H -3.866034 0.290875 0.000000<br>H -2.649716 2.450730 0.000000<br>N 1.338390 0.406565 0.000000<br>O -0.042477 2.693772 0.000000<br>H 0.899906 2.442709 0.000000<br>H 4.304254 -1.534248 -0.000000<br>H 4.107745 0.522974 0.000000<br>B 3.618665 -0.559498 -0.000000	C 0.737092 -2.782919 -0.000000<br>H 3.419763 -0.671202 -0.000000<br>H 2.865277 -3.073257 -0.000000<br>H 0.505595 -3.838389 -0.000000<br>N -1.058046 0.364564 0.000000<br>O -1.579867 -2.266277 -0.000000<br>H -2.114832 -1.453091 -0.000000<br>C -1.953981 2.549576 0.000000<br>C -2.874671 3.320295 0.000000<br>H -3.697438 3.991271 0.000000	C 0.536961 2.917831 0.000000<br>H 3.388669 1.043902 0.000000<br>H 2.631422 3.391734 0.000000<br>H 0.214707 3.949235 0.000000<br>N -0.979843 -0.381769 0.000000<br>O -1.729073 2.212392 0.000000<br>H -2.205113 1.365225 0.000000<br>Cl -1.899225 -2.836418 0.000000	C 0.821564 -2.743041 -0.000000<br>H 3.426193 -0.534675 -0.000000<br>H 2.957682 -2.954407 -0.000000<br>H 0.630314 -3.806428 -0.000000<br>N -1.083428 0.338301 0.000000<br>O -1.510317 -2.320992 0.000000<br>H -2.089088 -1.539901 0.000000<br>C -2.054562 2.484802 0.000000<br>N -2.962377 3.192851 0.000000
2-NH2	3-BH2	3-C2H	3-Cl
C 2.106936 0.177183 -0.004141<br>C -0.190420 0.274672 -0.000849<br>C -0.337841 -1.133958 0.000827<br>C 0.865540 -1.883785 -0.000495<br>C 2.071687 -1.249862 -0.006285<br>C -1.351234 1.094070 -0.000484<br>C -1.630649 -1.701946 0.003460<br>H 0.816534 -2.965395 -0.000131<br>H 2.997517 -1.808933 -0.018609<br>C -2.733761 -0.882465 0.003961<br>C -2.600632 0.519400 0.001985<br>H -1.738291 -2.778480 0.004394<br>H -3.725913 -1.312755 0.005508<br>H -3.472092 1.158800 0.001795<br>N 1.013125 0.906698 0.002736<br>O -1.198030 2.439712 -0.002572<br>H -0.238764 2.597767 -0.007246<br>N 3.318115 0.830167 -0.055984<br>H 3.294297 1.813683 0.155851<br>H 4.134523 0.339719 0.263878	C 2.201825 -0.076201 0.000000<br>C 0.000000 0.541121 -0.000000<br>C -0.457719 -0.805248 -0.000000<br>C 0.536676 -1.801272 0.000000<br>C 1.884426 -1.469047 0.000000<br>H 3.243798 0.225001 0.000000<br>C -0.944499 1.603709 -0.000000<br>C -1.848482 -1.067042 -0.000000<br>H 0.239762 -2.843612 0.000000<br>C -2.733566 -0.018271 -0.000000<br>C -2.290743 1.319838 -0.000000<br>H -2.196657 -2.090976 -0.000000<br>H -3.797596 -0.212046 -0.000000<br>H -2.999237 2.136038 -0.000000<br>N 1.314404 0.886781 0.000000<br>O -0.496302 2.875806 -0.000000<br>H 0.475624 2.821528 -0.000000<br>H 4.129580 -2.224355 0.000000<br>H 2.682533 -3.693072 0.000000<br>B 2.980858 -2.539588 0.000000	C 1.885648 0.495146 0.000000<br>C 0.000000 -0.805474 0.000000<br>C -0.868336 0.319418 0.000000<br>C -0.258940 1.589546 0.000000<br>C 1.117523 1.696471 0.000000<br>H 2.967101 0.560456 0.000000<br>C -0.546356 -2.118936 0.000000<br>C -2.267465 0.112657 0.000000<br>H -0.872335 2.480688 0.000000<br>C -2.761781 -1.168263 0.000000<br>C -1.910507 -2.291207 0.000000<br>H -2.931821 0.965937 0.000000<br>H -3.831312 -1.329570 0.000000<br>H -2.315142 -3.293156 0.000000<br>N 1.352771 -0.698978 0.000000<br>O 0.292665 -3.175642 0.000000<br>H 1.193781 -2.809366 0.000000<br>C 1.777610 2.956037 0.000000<br>C 2.352885 4.010937 0.000000<br>H 2.857329 4.945006 0.000000	C 1.808195 0.519431 0.000000<br>C 0.000000 -0.892092 0.000000<br>C -0.935294 0.178308 0.000000<br>C -0.402812 1.486949 0.000000<br>C 0.955457 1.647114 0.000000<br>H 2.882144 0.657338 0.000000<br>C -0.467176 -2.237017 0.000000<br>C -2.318469 -0.111130 0.000000<br>H -1.060953 2.344619 0.000000<br>C -2.734140 -1.420203 0.000000<br>C -1.818249 -2.490495 0.000000<br>H -3.033301 0.700152 0.000000<br>H -3.792223 -1.644755 0.000000<br>H -2.162365 -3.514699 0.000000<br>N 1.342326 -0.704678 0.000000<br>O 0.432490 -3.242176 0.000000<br>H 1.311631 -2.826812 0.000000<br>Cl 1.674929 3.239360 0.000000
3-CN	3-NH2	4-BH2	4-C2H
C 1.907819 0.479442 0.000000<br>C 0.000000 -0.790222 0.000000<br>C -0.855172 0.346960 0.000000<br>C -0.228551 1.607663 0.000000<br>C 1.148270 1.682934 0.000000<br>H 2.989769 0.530185 0.000000<br>C -0.565672 -2.095579 0.000000<br>C -2.256553 0.163121 0.000000<br>H -0.826097 2.509321 0.000000<br>C -2.768601 -1.110766 0.000000<br>C -1.933058 -2.244831 0.000000<br>H -2.907747 1.026211 0.000000<br>H -3.840051 -1.257390 0.000000<br>H -2.352583 -3.240631 0.000000	C -1.743317 -1.244783 -0.006231<br>C 0.398848 -0.432397 -0.001185<br>C -0.043788 0.917639 -0.002746<br>C -1.436963 1.134332 -0.003245<br>C -2.304261 0.063382 -0.005121<br>H -2.405355 -2.105919 -0.012655<br>C 1.791302 -0.726235 0.002302<br>C 0.922704 1.951051 -0.000617<br>H -1.818006 2.148785 -0.005526<br>C 2.259042 1.632841 0.002565<br>C 2.708366 0.297205 0.004111<br>H 0.599220 2.983245 -0.002237<br>H 2.995239 2.425735 0.003610<br>H 3.763390 0.064630 0.006328	C 2.286144 0.802429 0.000000<br>C -0.000000 0.631269 0.000000<br>C 0.021810 -0.795537 -0.000000<br>C 1.310863 -1.428557 -0.000000<br>C 2.422367 -0.597771 -0.000000<br>H 3.161738 1.441205 0.000000<br>C -1.241530 1.334321 0.000000<br>C -1.213664 -1.479679 -0.000000<br>H 3.415170 -1.027283 -0.000000<br>C -2.392864 -0.772330 -0.000000<br>C -2.422500 0.634184 0.000000<br>H -1.228736 -2.558075 -0.000000<br>H -3.332295 -1.309004 -0.000000<br>H -3.359214 1.172784 0.000000	C -2.000734 -1.394976 -0.000000<br>C 0.230312 -0.889066 -0.000000<br>C -0.000000 0.513972 0.000000<br>C -1.366227 0.931756 -0.000000<br>C -2.354605 -0.034629 -0.000000<br>H -2.776111 -2.152159 -0.000000<br>C 1.567788 -1.383172 0.000000<br>C 1.103358 1.393683 0.000000<br>H -3.396168 0.251225 -0.000000<br>C 2.378478 0.881806 0.000000<br>C 2.623760 -0.504552 0.000000<br>H 0.932740 2.460085 0.000000<br>H 3.223237 1.557366 0.000000<br>H 3.632776 -0.891030 0.000000

N 1.354252 -0.704525 0.000000	N -0.457303 -1.482368 -0.001751	N 1.115078 1.398061 0.000000	N -0.756193 -1.817447 -0.000000
O 0.252278 -3.166475 0.000000	O 2.183486 -2.019281 0.003738	O -1.226581 2.683040 0.000000	O 1.765308 -2.717565 -0.000000
H 1.162126 -2.823659 0.000000	H 1.368113 -2.549207 0.000984	H -0.289125 2.944312 0.000000	H 0.880082 -3.122410 -0.000000
C 1.822186 2.940388 0.000000	N -3.685637 0.210035 -0.065226	H 2.661662 -3.359189 -0.000000	C -1.706027 2.312920 0.000000
N 2.378943 3.949255 0.000000	H -4.229780 -0.564497 0.277922	H 0.648386 -3.740691 -0.000000	C -1.989533 3.480367 0.000000
	H -4.051727 1.099592 0.231506	B 1.541151 -2.956955 -0.000000	H -2.245098 4.510925 0.000000
4-Cl	4-CN	4-NH2	5-BH2
C -1.706696 -1.711795 -0.000000	C -2.016479 -1.370719 0.000000	C -1.280795 -2.041509 -0.011922	C 2.090158 1.599628 0.000000
C 0.425793 -0.888552 0.000000	C 0.221556 -0.874912 0.000000	C 0.426743 -0.533771 -0.000445	C -0.000000 0.671648 0.000000
C 0.000000 0.472008 -0.000000	C -0.000000 0.531123 -0.000000	C -0.442928 0.586460 -0.007448	C 0.484217 -0.660184 -0.000000
C -1.404910 0.659896 -0.000000	C -1.364091 0.940892 0.000000	C -1.846081 0.304975 -0.001275	C 1.891171 -0.793726 0.000000
C -2.255485 -0.414452 -0.000000	C -2.365074 -0.007200 0.000000	C -2.241551 -1.023486 -0.008883	C 2.687126 0.324365 0.000000
H -2.367409 -2.570803 -0.000000	H -2.793600 -2.125332 0.000000	H -1.604148 -3.076544 -0.015772	H 2.703856 2.492867 0.000000
C 1.820334 -1.185626 0.000000	C 1.556607 -1.375491 -0.000000	C 1.837344 -0.325175 0.008231	C -1.407970 0.920976 -0.000000
C 0.969026 1.498926 -0.000000	C 1.103328 1.408855 -0.000000	C 0.113815 1.886372 -0.019549	C -0.443255 -1.763167 -0.000000
H -3.325619 -0.273989 -0.000000	H -3.403935 0.287703 0.000000	H -3.292397 -1.281733 -0.009181	H 2.331536 -1.779555 0.000000
C 2.302843 1.170723 0.000000	C 2.375431 0.888686 -0.000000	C 1.478662 2.050298 -0.012052	H 3.764856 0.239105 0.000000
C 2.741274 -0.166416 0.000000	C 2.614482 -0.498376 -0.000000	C 2.352408 0.948690 0.005180	C -1.802437 -1.440249 -0.000000
H 0.653788 2.530647 -0.000000	H 0.940207 2.476942 -0.000000	H -0.520153 2.761143 -0.052626	C -2.295790 -0.131970 -0.000000
H 3.043116 1.959237 0.000000	H 3.223322 1.559957 -0.000000	H 1.896466 3.047831 -0.024660	H -2.517286 -2.252911 -0.000000
H 3.794723 -0.406685 0.000000	H 3.622277 -0.887720 -0.000000	H 3.424064 1.087133 0.010774	H -3.357522 0.069562 -0.000000
N -0.416021 -1.949380 0.000000	N -0.772608 -1.793106 0.000000	N 0.017776 -1.827708 -0.002944	N 0.787827 1.773129 0.000000
O 2.205859 -2.477367 0.000000	O 1.753272 -2.708103 -0.000000	O 2.640602 -1.408432 0.017950	O -1.828220 2.191551 0.000000
H 1.387447 -3.004350 0.000000	H 0.871792 -3.119394 0.000000	H 2.040277 -2.177236 0.014719	H -1.020240 2.740401 0.000000
Cl -2.074932 2.271899 -0.000000	C -1.694603 2.331424 0.000000	N -2.767543 1.322351 -0.030403	H -0.898241 -4.062263 -0.000000
	N -1.952133 3.454103 0.000000	H -3.721888 1.090555 0.183047	H 1.094404 -3.591868 -0.000000
		H -2.484371 2.236680 0.272511	B -0.041944 -3.232714 -0.000000
5-C2H	5-Cl	5-CN	5-NH2
C -2.753139 0.410652 0.000000	C 2.752064 0.745922 -0.000000	C -2.747007 0.389925 0.000000	C 2.642560 -0.294662 0.017434
C -0.721380 -0.645253 0.000000	C 0.875504 -0.569561 0.000000	C -0.703127 -0.638432 0.000000	C 0.504118 0.538062 0.013018
C -0.000000 0.576292 -0.000000	C 0.000000 0.553721 -0.000000	C 0.000000 0.591209 -0.000000	C -0.078673 -0.763007 -0.004414
C -0.768512 1.760355 -0.000000	C 0.609589 1.827965 -0.000000	C -0.780445 1.768038 0.000000	C 0.820254 -1.850482 -0.041336
C -2.137452 1.679118 0.000000	C 1.976727 1.923729 -0.000000	C -2.147562 1.666721 0.000000	C 2.173394 -1.622506 -0.027712
H -3.834028 0.329475 0.000000	H 3.834290 0.805857 -0.000000	H -3.826498 0.295157 0.000000	H 3.707876 -0.093849 0.034496
C -0.022849 -1.887765 0.000000	C 0.348632 -1.893313 0.000000	C 0.013974 -1.872701 0.000000	C -0.321948 1.694210 -0.001960
C 1.428974 0.542843 -0.000000	C -1.394534 0.305251 0.000000	C 1.426248 0.563116 -0.000000	C -1.498476 -0.892814 0.003296
H -0.264880 2.717197 -0.000000	H -0.009581 2.713048 -0.000000	H -0.292396 2.733472 -0.000000	H 0.446071 -2.863173 -0.102118
H -2.749122 2.570206 0.000000	H 2.466900 2.886864 -0.000000	H -2.770846 2.549473 0.000000	H 2.878462 -2.440941 -0.060556
C 2.062673 -0.688883 -0.000000	C -1.879423 -0.976848 0.000000	C 2.085666 -0.653456 -0.000000	C -2.259255 0.256436 -0.011941
C 1.352054 -1.898077 -0.000000	C -1.010696 -2.084232 0.000000	C 1.390851 -1.869495 -0.000000	C -1.683801 1.541874 -0.025704
H 3.143161 -0.717353 -0.000000	H -2.947092 -1.140479 0.000000	H 3.166349 -0.664752 -0.000000	H -3.339215 0.174949 -0.009051
H 1.874767 -2.843576 -0.000000	H -1.407620 -3.088890 0.000000	H 1.923502 -2.809010 -0.000000	H -2.314562 2.419316 -0.039373
N -2.074852 -0.714291 0.000000	N 2.225400 -0.456856 -0.000000	N -2.054252 -0.726302 0.000000	N 1.843895 0.747847 0.030528
O -0.733560 -3.030292 0.000000	O 1.204386 -2.934541 0.000000	O -0.679760 -3.020080 0.000000	O 0.256601 2.920559 0.006642
H -1.670763 -2.764231 0.000000	H 2.098438 -2.549480 0.000000	H -1.622000 -2.769966 0.000000	H 1.215796 2.763339 0.020984
C 2.179832 1.749665 -0.000000	Cl -2.524435 1.650094 -0.000000	C 2.157838 1.784693 -0.000000	N -2.085019 -2.170641 -0.042748

C 2.801825 2.779288 -0.000000		N 2.723017 2.790382 -0.000000	H -1.664200 -2.850769 0.572159
H 3.361153 3.681235 -0.000000			H -3.084204 -2.156446 0.091788
6-BH2	6-C2H	6-Cl	6-CN
C 1.724031 2.181571 0.000000	C 1.480247 -2.729201 -0.000000	C -2.670898 1.758469 -0.000000	C 1.490611 -2.700745 0.000000
C -0.000000 0.676964 0.000000	C -0.000000 -0.984462 -0.000000	C -1.064819 0.129118 -0.000000	C -0.000000 -0.965707 0.000000
C 0.881297 -0.438914 0.000000	C 1.031972 -0.008243 -0.000000	C 0.000000 1.068053 -0.000000	C 1.027420 0.014296 -0.000000
C 2.265775 -0.153625 0.000000	C 2.358971 -0.496269 -0.000000	C -0.366888 2.433016 -0.000000	C 2.358016 -0.463834 0.000000
C 2.687912 1.149745 0.000000	C 2.583204 -1.848200 -0.000000	C -1.693689 2.776895 -0.000000	C 2.589452 -1.813927 0.000000
H 2.041824 3.218041 0.000000	H 1.641745 -3.800997 -0.000000	H -3.723758 2.015350 -0.000000	H 1.658253 -3.771487 0.000000
C -1.409651 0.463762 -0.000000	C -1.362185 -0.564338 0.000000	C -0.770526 -1.266120 0.000000	C -1.367012 -0.558097 0.000000
C 0.338934 -1.740373 -0.000000	C 0.694235 1.360477 -0.000000	C 1.337068 0.610011 0.000000	C 0.682213 1.381503 -0.000000
H 2.976330 -0.970422 0.000000	H 3.183558 0.205016 -0.000000	H 0.404766 3.192027 -0.000000	H 3.177903 0.242661 -0.000000
H 3.739117 1.400706 0.000000	H 3.586708 -2.249623 -0.000000	H -2.002552 3.812667 -0.000000	H 3.594810 -2.210173 0.000000
C -1.035375 -1.944145 -0.000000	C -0.634463 1.744078 0.000000	C 1.568266 -0.740631 0.000000	C -0.649860 1.743245 -0.000000
C -1.904163 -0.815081 -0.000000	C -1.669765 0.771363 0.000000	C 0.533802 -1.695725 0.000000	C -1.685684 0.776214 -0.000000
H 1.009788 -2.589952 0.000000	H 1.475048 2.107590 -0.000000	H 2.156700 1.312887 0.000000	H 1.455382 2.136169 -0.000000
H -2.975616 -0.960949 -0.000000	H -2.703913 1.081634 0.000000	H 0.757842 -2.751170 0.000000	H -2.720918 1.082219 -0.000000
N 0.428252 1.962482 0.000000	N 0.231783 -2.318466 -0.000000	N -2.372667 0.478674 -0.000000	N 0.239854 -2.298181 0.000000
O -2.227474 1.538470 -0.000000	O -2.330065 -1.503233 0.000000	O -1.790637 -2.144580 0.000000	O -2.326074 -1.501135 0.000000
H -1.651367 2.322072 -0.000000	H -1.873482 -2.362718 0.000000	H -2.605865 -1.611999 -0.000000	H -1.867282 -2.359705 0.000000
H -2.773958 -3.563555 -0.000000	C -0.984017 3.125976 0.000000	Cl 3.218397 -1.329550 0.000000	C -1.002807 3.129751 -0.000000
H -0.850381 -4.304228 -0.000000	C -1.287712 4.288542 0.000000		N -1.288948 4.245781 -0.000000
B -1.597255 -3.375255 -0.000000	H -1.554550 5.315886 0.000000		
6-NH2	7-BH2	7-C2H	7-Cl
C 2.847562 0.217265 0.004613	C -1.190004 2.466998 0.000000	C -0.440382 -2.983907 -0.000000	C 0.339579 3.079508 0.000000
C 0.572980 0.466376 -0.000217	C -0.000000 0.510325 0.000000	C 0.000000 -0.737040 0.000000	C 0.000000 0.815699 0.000000
C 0.329876 -0.933839 -0.002479	C -1.167710 -0.291836 0.000000	C -1.376202 -0.391252 -0.000000	C 1.391066 0.532179 0.000000
C 1.479414 -1.758321 -0.000940	C -2.402024 0.394818 0.000000	C -2.295257 -1.462112 -0.000000	C 2.261014 1.643796 0.000000
C 2.726857 -1.188000 0.002390	C -2.414766 1.765865 0.000000	C -1.832431 -2.753267 -0.000000	C 1.740680 2.912518 0.000000
H 3.826327 0.682238 0.007233	H -1.182446 3.550643 0.000000	H -0.058021 -3.998148 -0.000000	H -0.088187 4.075451 0.000000
C -0.539756 1.359460 -0.001304	C 1.291099 -0.108825 0.000000	C 0.993901 0.283343 0.000000	C -0.952088 -0.245548 0.000000
C -0.994460 -1.414886 -0.002702	C -1.035950 -1.703375 0.000000	C -1.739894 0.976817 -0.000000	C 1.815804 -0.816321 0.000000
H 1.362762 -2.834718 -0.002981	H -3.325366 -0.170079 0.000000	H -3.357176 -1.251651 -0.000000	H 3.331283 1.481220 0.000000
H 3.618463 -1.799541 0.003082	H -3.344771 2.316811 0.000000	H -2.512905 -3.592925 -0.000000	H 2.382141 3.782227 0.000000
C -2.055824 -0.528062 -0.005655	C 0.210515 -2.262024 0.000000	C -0.769417 1.939430 -0.000000	C 0.888172 -1.824385 0.000000
C -1.818352 0.871565 -0.006360	C 1.420695 -1.498254 0.000000	C 0.617847 1.618675 0.000000	C -0.494094 -1.544759 0.000000
H -1.173650 -2.482575 -0.006431	H -1.925402 -2.319199 0.000000	H -2.786284 1.249994 -0.000000	H 2.872578 -1.045194 0.000000
H -2.647650 1.566063 -0.017525	H 0.307883 -3.339806 0.000000	H -1.040940 2.985547 -0.000000	H 1.198285 -2.859190 0.000000
N 1.805501 1.020467 0.003584	N -0.022829 1.864533 0.000000	N 0.448501 -2.016417 0.000000	N -0.505501 2.073382 0.000000
O -0.299196 2.686621 0.001838	O 2.355006 0.702943 0.000000	O 2.286254 -0.079552 0.000000	O -2.260764 0.055942 0.000000
H 0.670580 2.778914 0.003238	H 1.999447 1.611606 0.000000	H 2.289915 -1.053950 0.000000	H -2.308126 1.029158 0.000000
N -3.369515 -0.975079 -0.062939	H 3.798116 -1.584017 0.000000	C 1.592096 2.651933 0.000000	Cl -1.629656 -2.867118 0.000000
H -3.530246 -1.928522 0.215324	H 2.811857 -3.391276 0.000000	C 2.401697 3.539916 0.000000	
H -4.074698 -0.341899 0.274761	B 2.781860 -2.198422 0.000000	H 3.124119 4.317256 0.000000	
7-CN	7-NH2		
C -0.475861 -2.965403 0.000000	C -2.557998 -1.122190 0.004415		

C -0.000000 -0.728264 0.000000	C -0.386811 -0.390241 -0.004867		
C -1.367621 -0.356030 0.000000	C -0.778826 0.979759 -0.001183		
C -2.305740 -1.411645 0.000000	C -2.162062 1.242874 0.002535		
C -1.863992 -2.709431 0.000000	C -3.053939 0.198367 0.004727		
H -0.111100 -3.985798 0.000000	H -3.248707 -1.958225 0.008345		
C 1.013811 0.274897 -0.000000	C 0.981286 -0.732656 -0.006586		
C -1.711158 1.018481 0.000000	C 0.230589 1.968404 0.000953		
H -3.363811 -1.183647 0.000000	H -2.508423 2.268966 0.004299		
H -2.558717 -3.537263 0.000000	H -4.120830 0.370038 0.007421		
C -0.727745 1.966479 -0.000000	C 1.552035 1.605062 -0.004202		
C 0.649467 1.611386 -0.000000	C 1.958128 0.247574 -0.006499		
H -2.752919 1.307555 0.000000	H -0.047660 3.013745 0.003889		
H -0.980158 3.017106 -0.000000	H 2.320756 2.367860 -0.012449		
N 0.428802 -2.012743 0.000000	N -1.277423 -1.417935 -0.000380		
O 2.296838 -0.101214 -0.000000	O 1.347751 -2.043541 -0.000495		
H 2.295681 -1.076111 -0.000000	H 0.516615 -2.548806 0.006992		
C 1.644105 2.631542 -0.000000	N 3.299617 -0.103023 -0.063903		
N 2.423302 3.480715 -0.000000	H 3.951317 0.558876 0.322238		
	H 3.505163 -1.059134 0.177449		
**complex**			
2-BH2	2-C2H	2-Cl	2-CN
C 1.880142 0.546363 -1.183797	C 1.888155 0.876736 -1.086814	Re -0.702925 -0.572368 -0.633481	C 1.863759 0.888696 -1.108239
C 2.899930 1.204522 -1.859809	C 2.886868 1.652549 -1.669830	C -1.315958 4.841726 0.576906	C 2.846394 1.672177 -1.708078
C 4.203967 1.235997 -1.337376	C 4.199531 1.633357 -1.182793	H -0.258131 5.103908 0.520318	C 4.162115 1.680598 -1.229054
C 4.528311 0.622243 -0.148888	C 4.556353 0.851809 -0.107345	H -1.777831 5.465116 1.338944	C 4.537884 0.919750 -0.145247
C 3.525537 -0.064620 0.569424	C 3.579976 0.049815 0.516922	H -1.771964 5.087772 -0.383436	C 3.578249 0.111020 0.496057
C 2.210116 -0.094925 0.045936	C 2.246584 0.056502 0.026761	O 0.287571 -2.817219 -2.499664	C 2.241984 0.089722 0.014210
N 1.190362 -0.752519 0.678538	N 1.243313 -0.708189 0.589008	N -1.024383 2.409846 0.143722	N 1.254487 -0.682998 0.593159
C 1.412097 -1.403670 1.814274	C 1.558529 -1.451428 1.627017	N 1.291465 -0.585156 0.514986	C 1.587818 -1.406725 1.639266
C 2.699567 -1.399615 2.401875	C 2.850911 -1.523527 2.184669	N -1.306558 1.184351 0.648099	C 2.884534 -1.450546 2.189750
Re -0.732409 -0.678791 -0.411174	Re -0.703978 -0.541390 -0.626298	C -1.759657 -1.893474 0.285408	Re -0.702530 -0.563999 -0.612101
C -0.586738 4.901351 -0.321218	C -0.896577 4.919295 0.512600	C -2.137130 2.783964 1.976614	C -0.975563 4.908135 0.453010
H 0.495454 5.002105 -0.418746	H 0.180916 5.090786 0.489388	H -2.639485 3.267350 2.795814	H 0.098890 5.096412 0.420702
H -0.948835 5.724228 0.290673	H -1.328917 5.588500 1.252796	O -2.413734 -2.694218 0.802538	H -1.414071 5.580534 1.186655
H -1.021876 5.002975 -1.316721	H -1.298298 5.191259 -0.464842	C 1.743558 1.064028 -1.161914	H -1.387481 5.160152 -0.525533
O -0.122933 -3.352745 -1.817644	O 0.154054 -2.884001 -2.437108	O -3.105424 -0.155199 -2.506130	O 0.181905 -2.917442 -2.395963
N -0.630218 2.416166 -0.241735	N -0.797555 2.466455 0.112008	C 1.703047 -1.308642 1.533022	N -0.839742 2.451884 0.085470
N -1.061138 1.368890 0.502041	N -1.198773 1.275238 0.617857	C 2.653127 1.926853 -1.768081	N -1.218792 1.261533 0.610106
C -1.999312 -1.608124 0.712122	C -1.898284 -1.757663 0.268506	H 2.310886 2.524494 -2.601802	C -1.871800 -1.786648 0.306754
C -1.646721 3.303771 1.465631	C -1.934399 2.955002 1.902698	C 3.552239 0.368322 0.375191	C -1.973414 2.946960 1.876378
H -2.063821 4.007508 2.164134	H -2.420771 3.488808 2.699945	C 3.015023 -1.276367 2.046932	H -2.463436 3.483917 2.669262
O -2.814207 -2.157504 1.324458	O -2.634380 -2.494203 0.770604	H 3.265346 -1.909541 2.883009	O -2.592973 -2.527804 0.823502
O -3.103713 -0.261999 -2.356233	O -2.999676 0.053949 -2.582874	C 3.928965 -0.446059 1.465708	O -3.019237 -0.032333 -2.562241
H 2.674471 1.684491 -2.802386	H 2.624001 2.266785 -2.520130	H 4.944740 -0.405855 1.837022	H 2.568603 2.270353 -2.565057
H 2.856443 -1.927597 3.332899	H 3.019157 -2.165399 3.034545	C 4.435965 1.255726 -0.271151	H 3.068157 -2.077864 3.047275
C 3.735431 -0.743823 1.791694	C 3.850410 -0.780181 1.627029	H 5.460145 1.325950 0.067266	C 3.868627 -0.699162 1.615724

H 4.723576 -0.744805 2.234458	H 4.853176 -0.821163 2.032085	C -2.522918 0.298761 2.630650	H 4.874350 -0.718587 2.015016
H 5.537468 0.652013 0.237943	H 5.571110 0.839634 0.264841	H -3.335761 -0.234259 2.136730	H 5.554936 0.928863 0.220708
C -2.350205 1.084413 2.614662	C -2.550230 0.519169 2.566250	H -2.905516 0.706986 3.564127	C -2.546185 0.511032 2.577105
H -3.229562 0.566046 2.231798	H -3.388532 0.050556 2.050077	H -1.749960 -0.431114 2.865872	H -3.379988 0.022058 2.072665
H -2.668302 1.728005 3.432556	H -2.927455 0.969382 3.482447	C -1.996471 1.400424 1.774563	H -2.925020 0.967793 3.489388
H -1.675754 0.330244 3.016426	H -1.849908 -0.270299 2.834202	C -2.212930 -0.319257 -1.795340	H -1.831719 -0.263420 2.851465
C -1.694512 1.902359 1.553973	C -1.904642 1.562196 1.718308	O 0.489697 0.976097 -1.539427	C -1.922498 1.552391 1.710918
C -2.235596 -0.418552 -1.619020	C -2.148123 -0.176080 -1.840831	C -0.078144 -1.978657 -1.799839	C -2.159645 -0.238517 -1.822462
O 0.630362 0.499139 -1.607179	O 0.644454 0.890133 -1.505673	C 2.207212 0.266780 -0.070709	O 0.617494 0.876519 -1.519446
C -0.347165 -2.355033 -1.289269	C -0.162287 -2.009249 -1.757824	C 3.978458 2.014210 -1.324772	C -0.144277 -2.038537 -1.726768
H 4.972251 1.756549 -1.894074	H 4.946063 2.247322 -1.669207	H 4.653778 2.693561 -1.828049	H 4.895778 2.299589 -1.728504
C -0.957338 3.599613 0.303322	C -1.215446 3.504323 0.856275	C -1.502767 3.401747 0.913800	C -1.269676 3.493149 0.818042
H -0.096424 2.175415 -1.073429	H -0.212284 2.419503 -0.717540	H -0.465618 2.420211 -0.705142	H -0.258818 2.402801 -0.747004
B 0.192992 -2.188003 2.409013	C 0.474930 -2.313327 2.301216	Cl 0.591205 -2.389793 2.323074	C 0.522237 -2.276364 2.331990
H 0.000333 -3.312332 2.079279	C -0.365915 -2.982138 2.824377		N -0.280408 -2.931416 2.853782
H -0.463897 -1.712001 3.277812	H -1.112472 -3.575953 3.288874		
2-NH2	3-BH2	3-C2H	3-Cl
C 1.809682 0.596729 -1.235720	C 1.496624 0.907521 -1.414604	C 0.905533 1.483747 -1.474538	Re -0.833971 -0.794292 -0.509757
C 2.784582 1.270275 -1.964560	C 2.299088 1.743305 -2.187794	C 1.390158 2.593359 -2.159177	C -2.556821 4.258701 1.123033
C 4.111658 1.331869 -1.518397	C 3.638066 1.986876 -1.844676	C 2.709502 3.032690 -1.973920	H -1.678874 4.810058 0.782290
C 4.493823 0.728003 -0.342520	C 4.217546 1.417322 -0.734189	C 3.580306 2.399060 -1.115094	H -2.935703 4.747092 2.017735
C 3.534830 0.032809 0.423150	C 3.446963 0.559833 0.082140	C 3.137885 1.267885 -0.398511	H -3.320107 4.334844 0.346977
C 2.195114 -0.037767 -0.021517	C 2.092458 0.306821 -0.265578	C 1.803728 0.814915 -0.585014	O 0.114261 -2.703903 -2.737240
N 1.206494 -0.711222 0.674725	N 1.291164 -0.523027 0.476898	N 1.311714 -0.278299 0.070108	N -1.732765 1.998284 0.491887
C 1.530922 -1.312640 1.814498	C 1.791442 -1.098073 1.552351	C 2.088312 -0.938850 0.909287	N 1.260142 -0.264647 0.041432
C 2.860250 -1.272344 2.328788	C 3.127491 -0.911183 1.999912	C 3.416591 -0.542668 1.148042	N -1.498019 0.759669 0.988407
Re -0.767298 -0.674927 -0.443823	C 3.934656 -0.076281 1.238928	C 3.945932 0.542954 0.505784	C -1.040761 -2.324763 0.631506
C -0.745162 4.899057 -0.210175	Re -0.736360 -0.793592 -0.405693	Re -0.776557 -0.788946 -0.519813	C -2.320414 2.096712 2.585387
H 0.326811 5.018538 -0.375893	C -1.675311 4.693702 0.018286	C -2.505869 4.232242 1.201845	H -2.676780 2.442046 3.539750
H -1.075417 5.695631 0.452495	H -0.661097 5.015914 -0.223536	H -1.621948 4.788179 0.884813	O -1.118306 -3.254944 1.317099
H -1.246978 5.027853 -1.170541	H -2.093776 5.402237 0.729247	H -2.894977 4.701966 2.102136	C 0.828304 1.461609 -1.536312
O -0.102033 -3.303920 -1.911453	H -2.267741 4.742901 -0.896770	H -3.258734 4.327943 0.417815	O -3.736142 -1.169927 -1.495162
N -0.746968 2.412010 -0.211023	O 0.181010 -3.227863 -2.059992	O 0.195114 -2.651926 -2.776492	C 2.049681 -0.904210 0.884783
N -1.112557 1.336117 0.524810	N -1.219030 2.249654 -0.063649	N -1.680223 1.983690 0.531604	C 1.300446 2.557209 -2.251555
C -1.941239 -1.673032 0.695262	N -1.323394 1.109502 0.660550	N -1.455583 0.733669 1.003610	H 0.631498 3.052828 -2.941488
C -1.669202 3.227298 1.584601	C -1.441650 -2.018928 0.893572	C -1.002680 -2.343576 0.584532	C 3.075121 1.276098 -0.485016
H -2.053973 3.901561 2.329260	C -2.125295 2.847379 1.822911	C -2.294864 2.037744 2.618961	C 3.379676 -0.498981 1.097370
O -2.642387 -2.298422 1.380697	H -2.564402 3.424951 2.617075	H -2.662652 2.363057 3.576021	C 3.897157 0.573703 0.424441
O -3.170637 -0.287033 -2.338113	O -1.825414 -2.773393 1.684036	O -1.091874 -3.288417 1.248315	H 4.921252 0.881997 0.582344
H 2.493580 1.740701 -2.894103	O -3.439179 -0.861472 -1.898404	O -3.666697 -1.133450 -1.550895	C 3.504579 2.392426 -1.232117
H 3.069212 -1.771745 3.264544	H 1.868944 2.197430 -3.070123	H 0.731718 3.106228 -2.846607	H 4.518561 2.750598 -1.124321
C 3.834803 -0.619339 1.645189	H 4.962639 0.090796 1.537606	H 4.968770 0.844411 0.683756	C -1.779881 -0.372935 3.186236
H 4.847315 -0.592862 2.027218	H 5.249896 1.610695 -0.478675	H 4.593832 2.751520 -0.986241	H -2.475721 -1.156487 2.886295
H 5.517294 0.773583 0.003651	C -2.197528 0.485686 2.905138	C -1.769407 -0.446285 3.172000	H -2.026259 -0.072434 4.202636
C -2.242528 0.952548 2.706778	H -2.974518 -0.214011 2.597128	H -2.463549 -1.220794 2.845787	H -0.781663 -0.808749 3.193143
H -3.121554 0.406299 2.364225	H -2.548583 1.013480 3.789635	H -2.028158 -0.167515 4.191513	C -1.860554 0.805681 2.275874

H -2.535003 1.561151 3.560322	H -1.323008 -0.100911 3.183533	H -0.772637 -0.885334 3.182228	C -2.655430 -1.035685 -1.120686
H -1.510325 0.219607 3.044383	C -1.884548 1.462964 1.822794	C -1.834760 0.752398 2.286962	O -0.403183 1.010150 -1.625359
C -1.686093 1.822916 1.630940	C -2.435188 -0.840497 -1.334147	C -2.590580 -1.010965 -1.159537	C -0.235180 -1.990583 -1.903752
C -2.280902 -0.433737 -1.622055	O 0.232240 0.666453 -1.681841	O -0.325994 1.038304 -1.589536	C 1.740109 0.815234 -0.644038
O 0.545351 0.544667 -1.619396	C -0.157746 -2.318706 -1.439412	C -0.163101 -1.956072 -1.932037	C 2.620674 3.004367 -2.093284
C -0.346502 -2.321120 -1.362810	H 4.224970 2.639582 -2.477426	H 3.049535 3.897161 -2.529177	H 2.950701 3.857329 -2.671887
H 4.842608 1.861941 -2.114457	C -1.686600 3.321141 0.599481	C -2.181634 2.802645 1.471804	C -2.224538 2.836375 1.420239
C -1.057232 3.571798 0.392267	H -0.771405 2.158762 -0.971298	H -1.404974 2.150424 -0.432121	H -1.470590 2.144458 -0.478796
H -0.233879 2.204258 -1.064874	H 1.129427 -1.751922 2.103130	H 1.677350 -1.804950 1.405451	Cl 4.344236 -1.403604 2.234845
N 0.576444 -1.958687 2.547339	B 3.644852 -1.609774 3.263596	C 4.252670 -1.366039 2.145354	H 1.647969 -1.760314 1.405274
H -0.290419 -2.198805 2.098350	H 2.928404 -2.317934 3.897719	C 4.901445 -2.004953 2.919243	
H 0.891005 -2.598023 3.254978	H 4.769773 -1.439707 3.614111	H 5.477471 -2.572224 3.606353	

3-CN	3-NH2	4-BH2	4-C2H
C 0.944771 1.440833 -1.488077	C 1.393354 1.008639 -1.450947	C 1.537854 0.571803 -1.433339	C 1.255648 0.551748 -1.602309
C 1.452164 2.530788 -2.187633	C 2.137806 1.899916 -2.213409	C 2.408255 1.251283 -2.275630	C 2.055330 1.217364 -2.524225
C 2.775337 2.955504 -1.995724	C 3.476477 2.170509 -1.886634	C 3.754171 1.411083 -1.922645	C 3.428177 1.378495 -2.296299
C 3.627824 2.326208 -1.115599	C 4.106809 1.580199 -0.814214	C 4.273510 0.915035 -0.745340	C 4.047436 0.895419 -1.164470
C 3.161981 1.214716 -0.383234	C 3.393586 0.665910 -0.007505	C 3.441807 0.215000 0.149870	C 3.282034 0.210986 -0.200231
C 1.823822 0.776543 -0.576303	C 2.040218 0.388887 -0.336983	C 2.069653 0.048085 -0.210162	C 1.884998 0.037783 -0.423153
N 1.309591 -0.297469 0.093357	N 1.294060 -0.487454 0.394716	N 1.181882 -0.619666 0.581985	N 1.069578 -0.618549 0.454572
C 2.067731 -0.952763 0.953280	C 1.832765 -1.093063 1.435287	C 1.597766 -1.131821 1.731864	C 1.591657 -1.115788 1.562700
C 3.398655 -0.570309 1.199572	C 3.172718 -0.882493 1.839424	C 2.923411 -1.007787 2.160165	C 2.951177 -0.995194 1.878278
C 3.949960 0.495846 0.543334	C 3.939934 -0.001264 1.107739	C 3.876546 -0.345158 1.399571	C 3.781895 -0.341082 1.005913
Re -0.778932 -0.790565 -0.510439	Re -0.752193 -0.792562 -0.426468	Re -0.847369 -0.798021 -0.287881	Re -1.035140 -0.798496 -0.244587
C -2.457517 4.284918 1.098527	C -1.778745 4.660363 0.173718	C -1.277476 4.762587 -0.517726	C -1.439921 4.766468 -0.469409
H -1.562574 4.822769 0.781217	H -0.774166 5.003982 -0.078415	H -0.233752 4.958684 -0.768713	H -0.419322 4.956263 -0.805939
H -2.849960 4.776599 1.985549	H -2.193610 5.342201 0.912426	H -1.636950 5.584274 0.097242	H -1.744527 5.593699 0.167366
H -3.200336 4.376219 0.304454	H -2.389395 4.726194 -0.728229	H -1.849091 4.760387 -1.447208	H -2.085442 4.761212 -1.349156
O 0.191629 -2.708349 -2.721220	O 0.178018 -3.160404 -2.167570	O -0.153739 -3.485188 -1.633723	O -0.470020 -3.495462 -1.627362
N -1.655701 2.013234 0.479304	N -1.287199 2.227791 0.013522	N -1.051945 2.294389 -0.308925	N -1.215604 2.297600 -0.265762
N -1.453545 0.769290 0.976840	N -1.358666 1.066686 0.706711	N -1.273668 1.260161 0.537712	N -1.370461 1.269586 0.602930
C -1.038618 -2.321112 0.619859	C -1.367236 -2.069157 0.867509	C -1.667053 -1.794636 1.135126	C -1.737033 -1.780879 1.247898
C -2.292415 2.114558 2.558267	C -2.165373 2.756855 1.933687	C -1.928127 3.186624 1.472668	C -1.926216 3.206592 1.579769
H -2.666160 2.462635 3.504955	H -2.597537 3.304626 2.752433	H -2.323110 3.890060 2.184175	H -2.252845 3.916896 2.318597
O -1.148137 -3.252110 1.299959	O -1.685955 -2.855271 1.658177	O -2.116182 -2.413748 2.005044	O -2.114202 -2.391971 2.157131
O -3.662061 -1.114877 -1.567374	O -3.504269 -0.909350 -1.824666	O -3.538632 -0.794127 -1.809286	O -3.844819 -0.787826 -1.527549
H 0.808458 3.039662 -2.891784	H 1.672412 2.371218 -3.067956	H 2.030177 1.643201 -3.209746	H 1.597403 1.599723 -3.426043
H 4.974850 0.786623 0.727178	H 4.970676 0.184817 1.384899	H 5.315575 1.056158 -0.507375	H 5.106161 1.029630 -1.010352
H 4.644646 2.667186 -0.981910	H 5.139381 1.803271 -0.584167	C -2.236460 0.983602 2.815716	C -2.134508 1.013112 2.957221
C -1.807425 -0.365646 3.163019	C -2.174781 0.364641 2.949681	H -3.076194 0.332251 2.573025	H -2.988801 0.357802 2.787360
H -2.508575 -1.136586 2.843409	H -2.929468 -0.353958 2.630163	H -2.541822 1.640684 3.627579	H -2.376320 1.676814 3.784964
H -2.073477 -0.064419 4.174232	H -2.536713 0.859213 3.848931	H -1.426726 0.350600 3.176138	H -1.297072 0.384942 3.258214
H -0.816986 -0.817950 3.192562	H -1.278186 -0.197544 3.208114	C -1.817807 1.795704 1.636916	C -1.812764 1.815533 1.741867
C -1.846493 0.817104 2.255286	C -1.902227 1.377432 1.889268	C -2.541478 -0.798272 -1.234108	C -2.801009 -0.794847 -1.040749
C -2.588716 -0.999771 -1.166285	C -2.481349 -0.868933 -1.296274	O 0.259214 0.406146 -1.695542	O -0.040403 0.390863 -1.752118
O -0.291455 1.010121 -1.608644	O 0.127692 0.725440 -1.688304	C -0.407582 -2.482089 -1.129213	C -0.676637 -2.488160 -1.108990

C -0.166190 -1.992060 -1.893856	C -0.165262 -2.274409 -1.516136	H 4.408059 1.941334 -2.603042	H 4.019893 1.898169 -3.038461
H 3.133354 3.804765 -2.563020	H 4.028463 2.866363 -2.505457	C -1.427616 3.473373 0.215141	C -1.535156 3.482596 0.281825
C -2.156043 2.856324 1.398505	C -1.758183 3.271895 0.715618	H -0.599579 2.058449 -1.187808	H -0.845716 2.054188 -1.180116
H -1.367612 2.158208 -0.484188	H -0.855578 2.165920 -0.905602	H 0.866211 -1.661263 2.324393	H 0.922787 -1.639012 2.230340
H 1.639423 -1.803789 1.460730	H 1.204291 -1.782915 1.982441	H 3.201151 -1.447818 3.107775	H 3.329555 -1.419408 2.795265
C 4.212281 -1.386240 2.221271	N 3.639331 -1.523712 2.981597	B 5.318829 -0.266702 1.950092	C 5.276885 -0.214300 1.353091
N 4.825142 -2.000838 2.990862	H 3.174318 -2.382004 3.229385	H 5.546172 -0.768607 3.005289	C 6.436958 -0.115920 1.622492
	H 4.639902 -1.591317 3.070227	H 6.202985 0.271304 1.368295	H 7.466948 -0.028572 1.861684
4-Cl	4-CN	4-NH2	5-BH2
Re -1.088842 -0.802421 -0.253375	C 1.267122 0.554280 -1.595271	C 1.496106 0.552144 -1.455004	C 1.714742 0.278510 -1.219864
C -1.549769 4.759524 -0.441873	C 2.070221 1.221182 -2.513278	C 2.361722 1.217847 -2.316151	C 2.749538 0.818735 -1.984147
H -0.534630 4.961148 -0.787902	C 3.441892 1.383512 -2.279203	C 3.711935 1.380075 -1.985216	C 4.051028 0.831419 -1.495789
H -1.855687 5.580129 0.202797	C 4.056616 0.900403 -1.144919	C 4.234877 0.889680 -0.808174	C 4.429781 0.326414 -0.243807
H -2.204375 4.752970 -1.314872	C 3.287627 0.214693 -0.184448	C 3.398243 0.199199 0.094734	C 3.377376 -0.238834 0.560931
O -0.512151 -3.485880 -1.657487	C 1.891757 0.040264 -0.413632	C 2.029781 0.035535 -0.231195	C 2.048165 -0.259253 0.072374
N -1.299461 2.291842 -0.254813	N 1.073107 -0.617345 0.460119	N 1.138178 -0.614311 0.581270	N 1.003130 -0.781648 0.781562
N 1.021234 -0.605961 0.424876	C 1.590769 -1.114698 1.570267	C 1.586350 -1.108297 1.725610	C 1.235546 -1.294883 1.979508
N -1.435272 1.257463 0.609499	C 2.948765 -0.992966 1.891911	C 2.911788 -1.003397 2.136530	C 2.516424 -1.315844 2.544044
C -1.765551 -1.800031 1.240641	C 3.782693 -0.337566 1.023586	C 3.844763 -0.360451 1.335556	C 3.575966 -0.795010 1.844090
C -1.999493 3.183402 1.603223	Re -1.028329 -0.798935 -0.248426	Re -0.898661 -0.797231 -0.283966	Re -0.940579 -0.731203 -0.300079
H -2.325233 3.886295 2.349493	C -1.437503 4.765760 -0.471950	C -1.252762 4.770981 -0.529467	C -0.640004 4.838166 -0.700654
O -2.127260 -2.419908 2.150208	H -0.415613 4.956729 -0.803865	H -0.207474 4.952481 -0.785265	H 0.435581 4.897539 -0.875162
C 1.174451 0.577729 -1.627045	H -1.745719 5.592341 0.163931	H -1.599285 5.600328 0.082809	H -0.938689 5.717017 -0.134211
O -3.911758 -0.811831 -1.506954	H -2.079128 4.760372 -1.354541	H -1.827631 4.772082 -1.456997	H -1.138079 4.875568 -1.670797
C 1.559660 -1.104366 1.524627	O -0.454498 -3.494582 -1.630186	O -0.232112 -3.492966 -1.619593	O -0.487343 -3.534133 -1.502294
C 1.957970 1.256310 -2.553376	N -1.211696 2.296997 -0.268682	N -1.064166 2.300198 -0.311932	N -0.744420 2.368370 -0.420958
H 1.486959 1.639298 -3.448161	N -1.369392 1.268349 0.598748	N -1.294795 1.273555 0.539942	N -1.151283 1.395643 0.431205
C 3.218592 0.248768 -0.248113	C -1.735853 -1.782828 1.240402	C -1.720383 -1.779503 1.144770	C -2.036971 -1.554580 1.042731
C 2.921176 -0.972334 1.826734	C -1.931330 3.204272 1.574197	C -1.911654 3.213386 1.472867	C -1.618384 3.418524 1.272824
C 3.736358 -0.305235 0.949562	H -2.261906 3.913847 2.311968	H -2.290720 3.925546 2.184457	H -1.968561 4.187437 1.938484
C 3.967210 0.946071 -1.216299	O -2.116440 -2.394792 2.147621	O -2.172743 -2.390818 2.020022	O -2.659591 -2.075088 1.868813
H 5.026141 1.089706 -1.072432	O -3.832326 -0.790264 -1.543775	O -3.596713 -0.775545 -1.788357	O -3.431476 -0.416299 -2.099788
C -2.172137 0.980228 2.970093	H 1.615911 1.603602 -3.416898	H 1.971273 1.601677 -3.248679	H 2.516589 1.215017 -2.963107
H -3.021780 0.317607 2.805352	H 5.114519 1.035551 -0.986053	H 5.279881 1.052008 -0.587137	C -2.293180 1.315465 2.640510
H -2.411688 1.636844 3.804121	C -2.143577 1.009823 2.949500	C -2.241261 1.017611 2.825399	H -3.195148 0.773813 2.355448
H -1.325561 0.358544 3.258729	H -2.996476 0.353783 2.775506	H -3.085844 0.370467 2.588531	H -2.559182 2.030657 3.416261
C -1.870900 1.792617 1.756106	H -2.389683 1.672829 3.776535	H -2.538906 1.680480 3.635586	H -1.599487 0.592417 3.067334
C -2.862894 -0.811449 -1.031088	H -1.306870 0.382295 3.253840	H -1.434264 0.380376 3.184743	C -1.695809 2.030317 1.475928
O -0.121479 0.405087 -1.764288	C -1.817248 1.813233 1.736024	C -1.821083 1.820899 1.641017	C -2.509254 -0.536574 -1.420711
C -0.723070 -2.483590 -1.131192	C -2.790668 -0.796547 -1.052374	C -2.594245 -0.786223 -1.218965	O 0.473121 0.256188 -1.612939
C 1.821041 0.063240 -0.457484	O -0.028098 0.392226 -1.750890	O 0.213447 0.396142 -1.705342	C -0.652539 -2.488285 -1.050556
C 3.331503 1.429502 -2.338819	C -0.664377 -2.487769 -1.112173	C -0.477291 -2.484624 -1.117972	H 4.826679 1.256142 -2.120405
H 3.910369 1.959108 -3.084087	H 4.036375 1.904171 -3.018457	H 4.359801 1.905626 -2.674445	C -1.002182 3.601680 0.048538
C -1.624717 3.470549 0.302894	C -1.534810 3.481377 0.278145	C -1.416971 3.487250 0.210222	H -0.263219 2.063825 -1.259257
H -0.936819 2.057227 -1.174355	H -0.837540 2.054453 -1.181529	H -0.621174 2.051456 -1.192642	H 0.388389 -1.709338 2.506476
H 0.902902 -1.637850 2.196160	H 0.919465 -1.638940 2.234657	H 0.868357 -1.625620 2.346096	H 2.652213 -1.747985 3.524932

H 3.313210 -1.398034 2.737273	H 3.323502 -1.417326 2.810325	H 3.205237 -1.438989 3.081698	H 4.570079 -0.807622 2.264856
Cl 5.429320 -0.147659 1.325336	C 5.276012 -0.209533 1.377432	N 5.145692 -0.233712 1.730963	B 5.888015 0.404636 0.170079
	N 6.400849 -0.113093 1.643965	H 5.466999 -0.789335 2.503679	H 6.291056 -0.000616 1.216636
		H 5.844424 0.006471 1.052121	H 6.674027 0.895515 -0.580622
5-C2H	5-Cl	5-CN	5-NH2
C 1.519559 0.252273 -1.276579	Re -1.185710 -0.721299 -0.290985	C 1.528270 0.253737 -1.270225	C 1.672618 0.307362 -1.211512
C 2.543993 0.760169 -2.065788	C -0.773723 4.831586 -0.714971	C 2.554851 0.762554 -2.056045	C 2.709740 0.832857 -1.965812
C 3.872678 0.754391 -1.619551	H 0.305361 4.869706 -0.873121	C 3.882133 0.757469 -1.605644	C 4.031518 0.830034 -1.491966
C 4.212414 0.249140 -0.387849	H -1.064916 5.720637 -0.160578	C 4.218327 0.252016 -0.373054	C 4.376576 0.334102 -0.250997
C 3.223139 -0.287834 0.466572	H -1.255627 4.869943 -1.693216	C 3.226719 -0.285878 0.478079	C 3.341330 -0.185528 0.573375
C 1.876982 -0.280092 0.002761	O -0.772649 -3.532371 -1.488141	C 1.882019 -0.278835 0.010050	C 2.006979 -0.212748 0.072123
N 0.851774 -0.783225 0.753268	N -0.929572 2.366853 -0.417250	N 0.854784 -0.782876 0.757168	N 0.973306 -0.732323 0.802202
C 1.112327 -1.290200 1.946831	N 0.773729 -0.791763 0.766001	C 1.111915 -1.290091 1.951370	C 1.208599 -1.205797 2.014633
C 2.409967 -1.330496 2.477959	N -1.369425 1.410231 0.435423	C 2.407908 -1.329742 2.486552	C 2.485018 -1.183928 2.594925
C 3.458459 -0.836340 1.746507	C -2.263047 -1.529586 1.076893	C 3.458373 -0.834671 1.758560	C 3.541563 -0.677440 1.880298
Re -1.118799 -0.723637 -0.283494	C -1.809437 3.447431 1.254715	Re -1.112566 -0.724189 -0.285750	Re -0.961641 -0.744950 -0.299106
C -0.738808 4.830753 -0.717526	H -2.154990 4.228579 1.908531	C -0.734756 4.830589 -0.716710	C -0.687884 4.810293 -0.722550
H 0.338383 4.874049 -0.886854	O -2.872491 -2.041242 1.918886	H 0.342933 4.874628 -0.882644	H 0.396146 4.875377 -0.830088
H -1.028676 5.718958 -0.161086	C 1.467571 0.242528 -1.255705	H -1.026933 5.718420 -0.160881	H -1.027697 5.693867 -0.187217
H -1.230971 4.865646 -1.690781	O -3.704342 -0.398745 -2.049252	H -1.223886 4.865500 -1.691491	H -1.123641 4.832240 -1.722736
O -0.704157 -3.533940 -1.481909	C 1.019422 -1.301276 1.961633	O -0.692377 -3.533819 -1.483809	O -0.406584 -3.540215 -1.473733
N -0.879326 2.365606 -0.415631	C 2.502609 0.746154 -2.033737	N -0.874649 2.365251 -0.416090	N -0.793169 2.344043 -0.419092
N -1.305593 1.407752 0.442541	H 2.274193 1.143988 -3.012584	N -1.302995 1.406834 0.440417	N -1.248460 1.380967 0.416247
C -2.177926 -1.535753 1.096285	C 3.150355 -0.307861 0.504348	C -2.175498 -1.537448 1.090426	C -2.030965 -1.591305 1.052440
C -1.747240 3.443644 1.264201	C 2.311290 -1.348575 2.506085	C -1.748514 3.442165 1.261376	C -1.780513 3.407902 1.203448
H -2.089902 4.223787 1.920734	C 3.369719 -0.858870 1.786041	H -2.093730 4.221866 1.917094	H -2.177258 4.182563 1.835698
O -2.776095 -2.049499 1.945064	C 4.151054 0.225085 -0.339224	O -2.775998 -2.051863 1.937150	O -2.634686 -2.124059 1.886562
O -3.657022 -0.415505 -2.015987	C -2.548183 1.367822 2.626425	O -3.645539 -0.417083 -2.026091	O -3.455864 -0.534395 -2.108918
H 2.303509 1.155791 -3.042639	H -3.445626 0.826614 2.326719	H 2.317180 1.158354 -3.033512	H 2.490482 1.226866 -2.948819
C -2.461442 1.361906 2.645646	H -2.829225 2.095370 3.385241	C -2.465718 1.359502 2.639869	C -2.528071 1.312999 2.548838
H -3.359231 0.815927 2.355793	H -1.869700 0.646944 3.080767	H -3.362246 0.813050 2.347018	H -3.390509 0.739993 2.208061
H -2.738246 2.088885 3.406562	C -1.918058 2.062779 1.466958	H -2.745371 2.086046 3.400160	H -2.871389 2.035197 3.286998
H -1.774731 0.644900 3.093709	C -2.770865 -0.521093 -1.385891	H -1.779959 0.642781 3.089842	H -1.850392 0.617488 3.042300
C -1.846783 2.058705 1.479005	O 0.207727 0.252336 -1.629415	C -1.847847 2.057089 1.475398	C -1.862717 2.021646 1.417522
C -2.716149 -0.532495 -1.362176	C -0.922938 -2.483053 -1.038423	C -2.706647 -0.533696 -1.369372	C -2.530660 -0.614372 -1.426837
O 0.255890 0.255424 -1.637265	C 1.809116 -0.292945 0.026672	O 0.265738 0.256207 -1.634871	O 0.409809 0.285483 -1.609880
C -0.855007 -2.484886 -1.031762	C 3.826568 0.733307 -1.573812	C -0.845306 -2.485016 -1.033782	C -0.610288 -2.495521 -1.032311
H 4.648035 1.153326 -2.257986	H 4.610447 1.129052 -2.203770	H 4.659234 1.157114 -2.241510	H 4.810123 1.227063 -2.132544
C -1.121681 3.606173 0.041108	C -1.170479 3.608130 0.038325	C -1.119226 3.605508 0.040306	C -1.088098 3.579759 0.017560
H -0.404132 2.034589 -1.250200	H -0.447429 2.034352 -1.247231	H -0.396629 2.034819 -1.249276	H -0.266737 2.008720 -1.224209
H 0.274380 -1.687016 2.501369	H 0.173834 -1.694504 2.507054	H 0.272486 -1.687629 2.503143	H 0.364608 -1.617464 2.549050
H 2.564610 -1.757803 3.457852	H 2.453682 -1.777671 3.487053	H 2.559749 -1.757284 3.466781	H 2.615891 -1.564337 3.597306
H 4.466286 -0.862943 2.133917	Cl 5.825290 0.236992 0.187729	H 4.464997 -0.860764 2.149120	H 4.525673 -0.639315 2.326236
C 5.685964 0.267377 0.059274	H 4.373346 -0.890886 2.183806	C 5.690455 0.271038 0.078695	N 5.698614 0.393395 0.236180
C 6.829400 0.281529 0.406230		N 6.799331 0.285367 0.418973	H 6.027225 -0.464346 0.656143
H 7.844618 0.294094 0.714280			H 6.358649 0.698352 -0.463294

6-BH2	6-C2H	6-Cl	6-CN
C 1.878073 0.175425 -0.912209	C 1.796683 -0.016342 -0.710891	Re -1.185566 -0.586381 -0.443873	C 1.797373 -0.006973 -0.722464
C 3.041297 0.655423 -1.497872	C 3.046566 0.362657 -1.188193	C 0.127708 4.838833 -0.213118	C 3.042464 0.377558 -1.207800
C 4.309979 0.531652 -0.873889	C 4.196692 0.086236 -0.437020	H 1.207938 4.704752 -0.136084	C 4.198190 0.110343 -0.461908
C 4.403505 -0.089959 0.368575	C 4.169967 -0.555743 0.777376	H -0.143817 5.724942 0.355767	C 4.181701 -0.527592 0.754799
C 3.256179 -0.595985 1.009305	C 2.920372 -0.958500 1.294018	H -0.111164 5.023605 -1.261665	C 2.937247 -0.935743 1.279553
C 1.997980 -0.461230 0.362773	C 1.741082 -0.690135 0.551632	O -0.925468 -3.347382 -1.791110	C 1.752453 -0.676721 0.542634
N 0.841576 -0.931959 0.920482	N 0.501451 -1.055565 0.991557	N -0.454391 2.420437 -0.205980	N 0.517335 -1.047754 0.990491
C 0.896117 -1.529229 2.099876	C 0.390864 -1.681239 2.152511	N 0.439817 -1.048826 1.005892	C 0.416605 -1.670050 2.154155
C 2.096906 -1.700449 2.808609	C 1.504752 -1.982865 2.952165	N -1.213514 1.496380 0.431213	C 1.536501 -1.962511 2.948807
C 3.268591 -1.239640 2.267629	C 2.759049 -1.627446 2.528647	C -2.660029 -1.287066 0.564947	C 2.786431 -1.601348 2.517311
Re -0.938681 -0.661094 -0.387164	Re -1.122934 -0.576100 -0.453791	C -1.522982 3.520627 1.338167	Re -1.117541 -0.582617 -0.447693
C -0.143555 4.863495 -0.408745	C 0.216645 4.841158 -0.189744	H -1.889469 4.303913 1.977855	C 0.192174 4.843130 -0.209379
H 0.947164 4.834242 -0.424423	H 1.296283 4.701396 -0.114526	O -3.518945 -1.741329 1.195746	H 1.272989 4.709837 -0.139516
H -0.449770 5.739858 0.157661	H -0.050120 5.724928 0.385001	C 1.731553 -0.014216 -0.702012	H -0.076588 5.727327 0.363778
H -0.488033 4.987586 -1.436663	H -0.022237 5.033739 -1.236882	O -3.147190 0.262639 -2.673700	H -0.053445 5.030726 -1.255864
O -0.534856 -3.422726 -1.697052	O -0.877294 -3.329699 -1.818774	C 0.331205 -1.667637 2.170705	O -0.863372 -3.339445 -1.804573
N -0.482325 2.402709 -0.281810	N -0.377085 2.425593 -0.197452	C 2.980024 0.367757 -1.180638	N -0.387648 2.424179 -0.205634
N -1.093279 1.435285 0.443456	N -1.140099 1.501166 0.434534	H 3.054417 0.860504 -2.138292	N -1.141907 1.497564 0.433615
C -2.245478 -1.467267 0.765296	C -2.599885 -1.276085 0.551871	C 2.857972 -0.938195 1.309813	C -2.584998 -1.287613 0.568314
C -1.524828 3.452385 1.314827	C -1.439037 3.521071 1.354606	C 1.445818 -1.958809 2.973219	C -1.447534 3.518874 1.348387
H -1.912916 4.220435 1.960275	H -1.801197 4.302028 1.999584	C 2.698768 -1.600053 2.548530	H -1.810717 4.299954 1.992641
O -2.997215 -1.980785 1.482157	O -3.460435 -1.730214 1.180537	C 4.106076 -0.532717 0.791697	O -3.439520 -1.744513 1.203164
O -3.159205 -0.064557 -2.449936	O -3.082367 0.296503 -2.676429	C -2.854394 1.508262 2.301916	O -3.093904 0.271044 -2.662684
H 2.967909 1.130919 -2.467111	H 3.122506 0.861119 -2.142763	H -3.729555 1.144970 1.763183	H 3.110391 0.873157 -2.164462
C -2.569420 1.358288 2.445018	C -2.779292 1.509046 2.306737	H -3.190619 2.221549 3.051778	C -2.771021 1.502470 2.314605
H -3.448559 0.898119 1.993804	H -3.656655 1.153399 1.766488	H -2.411943 0.656377 2.816652	H -3.649213 1.139926 1.780318
H -2.907363 2.061655 3.203466	H -3.111432 2.219159 3.061416	C -1.880467 2.162534 1.381167	H -3.103187 2.213258 3.068637
H -2.006400 0.568117 2.940100	H -2.340505 0.651788 2.815656	C -2.423162 -0.055333 -1.836563	H -2.324566 0.649507 2.824070
C -1.742871 2.068959 1.427013	C -1.803019 2.164467 1.389296	O 0.612638 0.223895 -1.343947	C -1.803495 2.160328 1.389673
C -2.337615 -0.288288 -1.674627	C -2.359157 -0.030263 -1.841975	C -1.019811 -2.317491 -1.284976	C -2.364343 -0.048681 -1.831036
O 0.683223 0.292090 -1.453935	O 0.678375 0.231224 -1.350302	C 1.678068 -0.680234 0.564721	O 0.674232 0.231955 -1.356668
C -0.682105 -2.391895 -1.205164	C -0.966241 -2.302605 -1.306023	C 4.130796 0.101655 -0.426732	C -0.955481 -2.311119 -1.294859
C -0.712354 3.632134 0.209368	C -0.523092 3.654644 0.326607	C -0.606774 3.652075 0.310128	C -0.537919 3.654169 0.315006
H 0.092053 2.078856 -1.055615	H 0.225555 2.076908 -0.936452	H 0.150578 2.069969 -0.942229	H 0.213009 2.076420 -0.946682
H -0.037300 -1.896515 2.501732	H -0.604945 -1.963912 2.461508	H -0.663508 -1.953133 2.480635	H -0.575880 -1.957377 2.469488
H 2.074834 -2.198506 3.766790	H 1.353965 -2.497318 3.889991	H 1.296670 -2.468014 3.914167	H 1.393731 -2.474589 3.889185
H 4.207986 -1.364018 2.790663	H 3.630848 -1.855553 3.127874	H 3.571119 -1.820148 3.149946	H 3.662742 -1.822374 3.112597
H 5.365665 -0.191314 0.852664	H 5.078852 -0.752086 1.325107	H 5.015406 -0.721198 1.341448	H 5.094637 -0.716818 1.298281
B 5.580028 1.073046 -1.554437	C 5.553767 0.529818 -1.014238	Cl 5.674559 0.610117 -1.086016	C 5.549559 0.559728 -1.047958
H 5.517879 1.611528 -2.615592	C 6.606822 0.874027 -1.462145		N 6.567473 0.898227 -1.489397
H 6.638020 0.958541 -1.017735	H 7.541794 1.179638 -1.859826		
6-NH2	7-BH2	7-C2H	7-Cl
C 1.858091 0.110544 -0.872623	C 1.926820 0.327863 -0.878099	C 1.952969 0.019076 -0.541687	Re -1.029379 -0.503457 -0.506646
C 3.036213 0.548428 -1.451798	C 3.058826 0.973946 -1.432097	C 3.239412 0.468575 -0.836997	C 0.430752 4.753961 0.694233
C 4.281745 0.399102 -0.798869	C 4.313044 0.787247 -0.776185	C 4.366740 0.011064 -0.138439	H 1.505289 4.565524 0.687846

C 4.353348 -0.189642 0.454627	C 4.478956 0.046960 0.361201	C 4.252452 -0.899359 0.884022	H 0.223643 5.513013 1.444938
C 3.181596 -0.648946 1.081108	C 3.354720 -0.583155 0.949908	C 2.977502 -1.383311 1.247735	H 0.159321 5.159900 -0.281657
C 1.932227 -0.505413 0.419157	C 2.089353 -0.440176 0.331909	C 1.841492 -0.918138 0.533918	O -0.837796 -2.980684 -2.331917
N 0.759005 -0.937261 0.959235	N 0.960715 -1.010207 0.844869	N 0.573706 -1.335916 0.832785	N -0.209615 2.388118 0.273998
C 0.770382 -1.512576 2.153148	C 1.047100 -1.724789 1.956985	C 0.400238 -2.203818 1.816640	N 0.490424 -1.323693 0.896469
C 1.956590 -1.689409 2.881179	C 2.262387 -1.919328 2.629252	C 1.468248 -2.715114 2.570024	N -1.000942 1.387515 0.731969
C 3.149676 -1.265660 2.353833	C 3.408774 -1.354869 2.130918	C 2.747396 -2.309254 2.287524	C -2.590401 -1.287009 0.287981
Re -0.987625 -0.649095 -0.389512	Re -0.852473 -0.678410 -0.395003	Re -0.980042 -0.494732 -0.519840	C -1.272358 3.229288 1.977272
C -0.013097 4.848390 -0.416396	C -0.332338 4.874760 -0.049718	C 0.423152 4.743524 0.824127	H -1.627595 3.897918 2.741355
H 1.076008 4.780755 -0.402715	H 0.756548 4.918178 -0.105582	H 1.500055 4.571436 0.795065	O -3.504694 -1.794470 0.786835
H -0.303162 5.736856 0.139667	H -0.671146 5.690630 0.584381	H 0.217164 5.475420 1.601629	C 1.910958 -0.032954 -0.497669
H -0.325814 4.981352 -1.453382	H -0.723507 5.040846 -1.054559	H 0.130301 5.175855 -0.134076	O -2.824991 0.853485 -2.622732
O -0.621978 -3.434186 -1.658644	O -0.411010 -3.315487 -1.930854	O -0.782003 -2.910203 -2.425425	C 0.288747 -2.157401 1.904225
N -0.443758 2.401731 -0.294456	N -0.532758 2.396075 -0.091240	N -0.189674 2.382775 0.339168	C 3.208401 0.388363 -0.786330
N -1.103904 1.458643 0.418664	N -1.043141 1.343457 0.594907	N -0.959052 1.356569 0.777462	C 2.886502 -1.392585 1.351143
C -2.337477 -1.409800 0.742789	C -2.125459 -1.605747 0.702971	C -2.516676 -1.326090 0.273745	C 1.337165 -2.659926 2.690320
C -1.483642 3.491699 1.276633	C -1.526612 3.265363 1.639158	C -1.237027 3.154017 2.084552	C 2.626465 -2.281711 2.415717
H -1.858615 4.274441 1.912142	H -1.913530 3.960958 2.362648	H -1.589589 3.792914 2.874864	C 4.174033 -0.938836 0.992855
O -3.118862 -1.897194 1.446403	O -2.855404 -2.191292 1.385560	O -3.415450 -1.862573 0.770431	C -2.668266 1.115563 2.557870
O -3.149533 -0.023782 -2.505258	O -3.119694 0.030593 -2.370993	O -2.828694 0.901136 -2.563702	H -3.559551 0.908144 1.965372
H 2.991659 1.009241 -2.430354	C -2.383927 1.039042 2.668292	C -2.592827 1.002486 2.619538	H -2.971270 1.679043 3.438150
C -2.622570 1.437007 2.388896	H -3.284914 0.593318 2.246341	H -3.490401 0.800525 2.034698	H -2.265983 0.158566 2.887284
H -3.503458 0.998706 1.919720	H -2.677661 1.658344 3.513491	H -2.889792 1.533423 3.521831	C -1.664557 1.895141 1.777271
H -2.957885 2.155020 3.134766	H -1.761779 0.226518 3.040920	H -2.171559 0.041713 2.912176	C -2.162560 0.346878 -1.828841
H -2.093627 0.634683 2.901660	C -1.661866 1.867211 1.659707	C -1.613095 1.821116 1.848513	O 0.847850 0.360231 -1.148670
C -1.751937 2.116655 1.386645	C -2.281779 -0.235169 -1.627657	C -2.146397 0.379756 -1.796654	C -0.906423 -2.057247 -1.647459
C -2.349967 -0.258685 -1.709249	O 0.732509 0.398490 -1.387030	O 0.874013 0.416631 -1.163296	C 1.768797 -0.933920 0.604952
O 0.678982 0.251638 -1.440149	C -0.571992 -2.331830 -1.354655	C -0.852973 -2.009873 -1.711074	C 4.317778 -0.063002 -0.055768
C -0.755524 -2.394405 -1.180679	C -0.796847 3.571579 0.504651	C -0.322697 3.482793 1.099657	C -0.338020 3.513479 0.997293
C -0.640673 3.639770 0.189666	H 0.017140 2.178029 -0.915086	H 0.418675 2.185032 -0.449402	H 0.407904 2.156741 -0.498121
H 0.138761 2.054780 -1.052042	H 0.132807 -2.165944 2.326011	H -0.614687 -2.516342 2.015484	H -0.733697 -2.448799 2.096520
H -0.177884 -1.852402 2.542407	H 2.274765 -2.515317 3.530287	H 1.263399 -3.423898 3.358903	H 1.109520 -3.340337 3.497716
H 1.911291 -2.165258 3.850193	H 4.357703 -1.493635 2.632393	H 3.587073 -2.691860 2.853249	H 3.451441 -2.658275 3.006567
H 4.073034 -1.401218 2.902242	H 5.450586 -0.064387 0.822656	H 5.129245 -1.246006 1.412277	H 5.037235 -1.281185 1.545722
H 5.305647 -0.299630 0.956508	H 5.174939 1.271346 -1.217621	H 5.336602 0.392754 -0.424097	Cl 3.452089 1.520765 -2.093594
N 5.419830 0.900427 -1.415813	B 2.973971 1.859370 -2.669639	C 3.421365 1.505867 -1.960615	H 5.297552 0.295420 -0.337720
H 6.300555 0.537542 -1.091645	H 1.938547 2.104174 -3.202487	C 3.562556 2.310779 -2.832514	
H 5.378425 0.998327 -2.416370	H 3.975275 2.343710 -3.102826	H 3.687914 3.025433 -3.606643	
**7-CN**	**7-NH2**	**-H**	
C 1.949234 0.038221 -0.559864	C 1.930497 0.180997 -0.808865	Re -0.719899 -0.779293 -0.307505	
C 3.227466 0.499134 -0.872713	C 3.097975 0.681184 -1.396487	C -0.812747 4.788804 -0.694896	
C 4.365659 0.069572 -0.174027	C 4.331639 0.535714 -0.728223	H 0.263681 4.926137 -0.810392	
C 4.270687 -0.823193 0.865833	C 4.431221 -0.089237 0.491999	H -1.205516 5.643667 -0.149367	
C 3.004885 -1.317132 1.247622	C 3.277270 -0.610426 1.113330	H -1.257524 4.789934 -1.691331	
C 1.857802 -0.880307 0.533603	C 2.028435 -0.466342 0.439972	O 0.008090 -3.528519 -1.497186	
N 0.597826 -1.309195 0.849288	N 0.860044 -0.959326 0.966435	N -0.751304 2.318566 -0.410929	
		N 1.195790 -0.666033 0.819061	

C 0.442843 -2.160947 1.850195	C 0.897677 -1.577795 2.135231	N -1.135140 1.319941 0.419639	
C 1.522826 -2.644049 2.605116	C 2.082929 -1.751499 2.866095	C -1.761020 -1.697624 1.018018	
C 2.794437 -2.226623 2.305932	C 3.265326 -1.272132 2.354488	C -1.795765 3.301552 1.226318	
Re -0.976435 -0.513805 -0.507316	Re -0.887360 -0.668645 -0.379731	H -2.239192 4.042882 1.867365	
C 0.366098 4.766523 0.729727	C -0.015851 4.843872 -0.132718	O -2.348031 -2.271742 1.835846	
H 1.444972 4.608170 0.696043	H 1.071622 4.767281 -0.175057	C 1.858309 0.446914 -1.175841	
H 0.155806 5.509903 1.495086	H -0.272051 5.689219 0.501736	O -3.193430 -0.691113 -2.155727	
H 0.060807 5.177129 -0.234131	H -0.373301 5.058017 -1.141431	C 1.455643 -1.155012 2.020490	
O -0.759516 -2.961319 -2.369465	O -0.439457 -3.387099 -1.760831	C 2.866544 1.055137 -1.914730	
N -0.218547 2.389306 0.292790	N -0.405039 2.387593 -0.134983	H 2.629679 1.435887 -2.898789	
N -0.971076 1.361316 0.755475	N -1.060963 1.400381 0.519004	C 3.521222 0.052269 0.629669	
C -2.496248 -1.350558 0.312556	C -2.213652 -1.512855 0.720839	C 2.729919 -1.078150 2.605393	
C -1.263862 3.178828 2.031210	C -1.485522 3.381702 1.471699	H 2.882310 -1.497101 3.589370	
H -1.619381 3.827535 2.812150	H -1.882222 4.125819 2.139627	C 3.754353 -0.482204 1.916364	
O -3.384285 -1.889581 0.825536	O -2.981641 -2.051221 1.401689	H 4.745735 -0.417910 2.346169	
O -2.857804 0.819429 -2.563105	O -3.063059 -0.017757 -2.473623	C 4.508188 0.680456 -0.159609	
C -2.587053 1.019845 2.615544	C -2.609392 1.257383 2.461275	H 5.516764 0.770482 0.219002	
H -3.485922 0.795247 2.041039	H -3.467025 0.815727 1.953850	C -2.393030 1.152359 2.559900	
H -2.884757 1.563428 3.510030	H -2.980846 1.935961 3.226680	H -3.220478 0.528883 2.221159	
H -2.150964 0.070306 2.922774	H -2.071646 0.448817 2.954848	H -2.774984 1.845259 3.307052	
C -1.623752 1.836932 1.822494	C -1.734286 1.999169 1.508131	H -1.668244 0.497424 3.041797	
C -2.163260 0.321403 -1.791544	C -2.258195 -0.262720 -1.685631	C -1.783796 1.911250 1.429635	
O 0.860746 0.409907 -1.180862	O 0.750738 0.318715 -1.403104	C -2.276383 -0.726142 -1.459387	
C -0.837495 -2.048932 -1.671326	C -0.604666 -2.372265 -1.240457	O 0.614754 0.337805 -1.593848	
C -0.360914 3.501364 1.033830	C -0.628710 3.596676 0.406713	C -0.259889 -2.501349 -1.050216	
H 0.386855 2.185051 -0.496388	H 0.190149 2.087735 -0.903445	C 2.201501 -0.063562 0.115470	
H -0.566405 -2.483119 2.062107	H -0.040333 -1.961748 2.510527	C 4.169445 1.163391 -1.403239	
H 1.332979 -3.340749 3.408382	H 2.046122 -2.266187 3.814729	H 4.926672 1.639815 -2.012284	
H 3.643082 -2.587560 2.872514	H 4.193669 -1.401542 2.896494	C -1.124892 3.528824 0.037842	
H 5.155702 -1.148383 1.394021	H 5.390804 -0.188665 0.980201	H -0.206205 2.034374 -1.220751	
H 5.328319 0.458690 -0.473684	H 5.222197 0.933787 -1.199631	H 0.637626 -1.633015 2.539724	
C 3.387695 1.517757 -2.016537	N 2.988264 1.359198 -2.602592		
N 3.508386 2.285031 -2.878120	H 3.813917 1.380465 -3.178144		
	H 2.145998 1.139110 -3.113565		
